# Gel-Based Ionic Circuits

**DOI:** 10.1021/acs.chemrev.5c00245

**Published:** 2025-09-02

**Authors:** Hyunjae Yoo, Yun Hyeok Lee, Min-Gyu Lee, Jeong-Yun Sun

**Affiliations:** † Department of Materials Science and Engineering, Seoul National University, Seoul 08826, Republic of Korea; ‡ Research Institute of Advanced Materials (RIAM), Seoul National University, Seoul 08826, Republic of Korea

## Abstract

Ionic circuits have emerged as a promising candidate to bridge the gap between biological and artificial systems by applying the mechanically compliant and adaptive nature of gels as ionic conductors. Gel-based ionic circuits exploit the intrinsic characteristics of ions, such as their mass, diversity, and local accumulation, to achieve selectivity, hysteresis, and chemical-electric signal transduction. Their dynamic and nonlinear behaviors not only emulate traditional solid-state electronic systems but also exhibit unique functionalities and operating mechanisms extending beyond established electronic paradigms. In this review, we categorize gel-based ionic circuits into four major functional classes: passive circuit elements, active circuit elements, power sources, and noncircuit elements. We comprehensively discuss the fundamental operating principles, materials strategies, and current challenges, eventually highlighting opportunities for future advancement in ionic devices.

## Graphical abstract

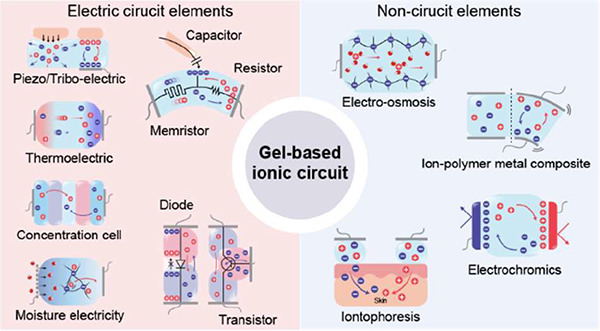

## 1 Introduction

Ions, along with electrons, are fundamental charge carriers in nature. Unlike electrons, ions exhibit several distinct physical and chemical properties that have led nature to rely on the language of “ions”. First, ions exist in a diverse range of types, each with distinctive conductivities, effective charges, and electrochemical properties. Second, ions have thousands of times greater mass and momentum than electrons, making their transport dynamics fundamentally different. Third, while electrons disappear upon combination with holes, anions and cations can accumulate locally, creating spatial charge distributions. These properties lead to key phenomena such as selectivity, hysteresis, and chemical-electric signal transduction, which, in turn, enable dynamic, nonlinear behaviors critical for energy conversion, signal transmission, and physiological processes (Figure 1a).^1^

**Figure 1:**
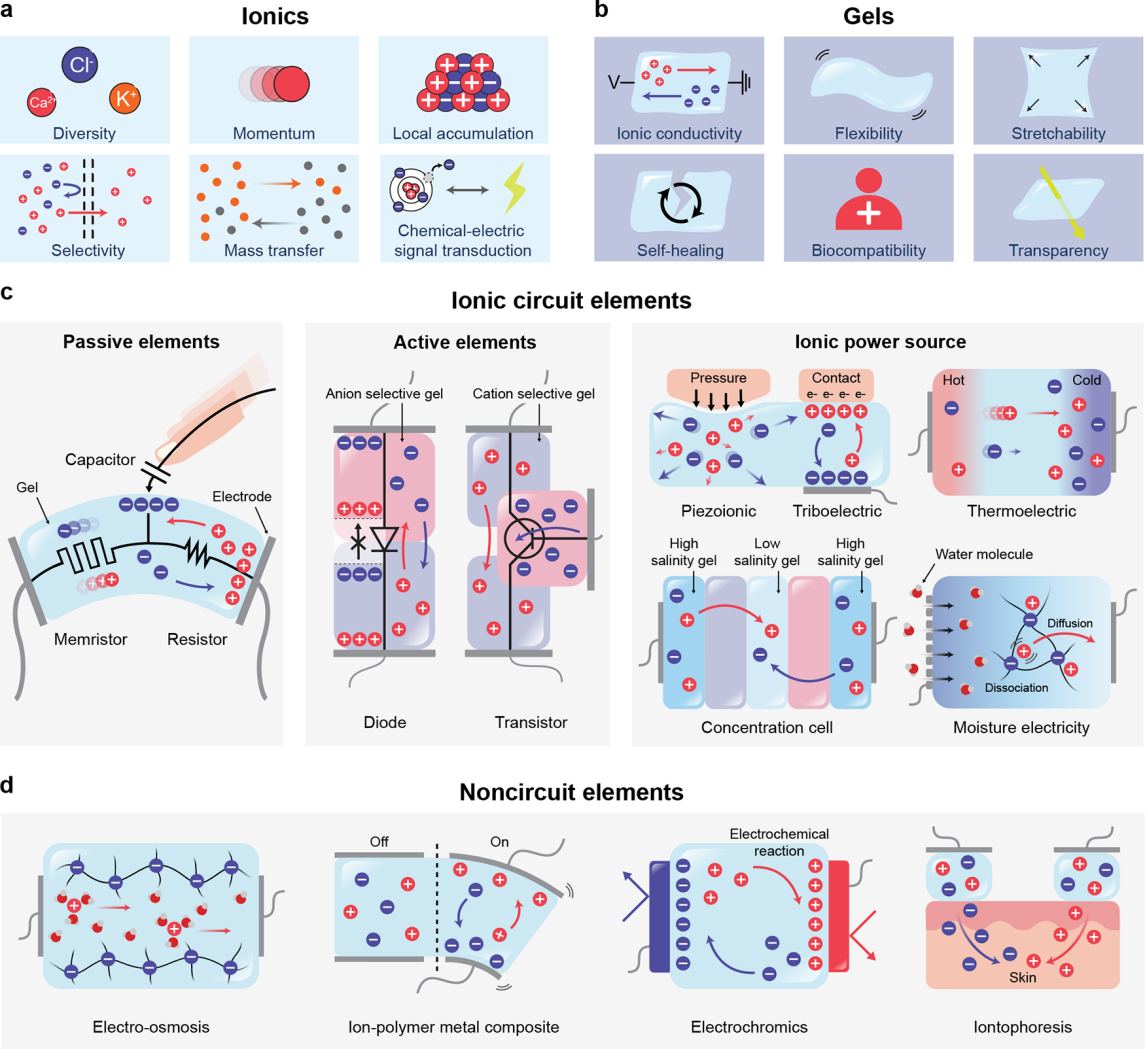
Gel-based ionic circuits. (a) Unique characteristics of ions compared to electrons. (b) Key advantages of gels as ionic conductors. (c,d) Schematic illustrations of representative gel-based (c) ionic circuit elements and (d) noncircuit elements.

Nature exploits ionic mechanisms across a wide range of biological systems. In neural networks, a variety of ions such as Na^+^, K^+^, Ca^2+^, and neurotransmitters facilitate both signal transmission and synaptic plasticity, reinforcing neuronal connections through bidirectional communication between pre- and post-synaptic neurons.^2,3^ Beyond the nervous system, biological actuators such as muscles and chromatophores rely on finely tuned ionic fluxes to drive mechanical responses.^4^ Meanwhile, sensory and bioelectrical structures such as the cochlea, ampullae of Lorenzini, and electrocytes of electric eel utilize ion transport for signal detection and energy conversion.^5–7^ Inspired by nature-designed ionic systems, artificial ionic systems have emerged as a complementary approach to human-made electronics, offering enhanced flexibility, stretchability, biocompatibility, and dynamic signal modulation.^8,9^ These systems share the fundamentally same “circuit” structure as solid-state electronics, yet the unique properties of ions allow ionic systems not only to function analogously to electronic circuit elements but also to enable exclusive applications beyond conventional circuits. Advances in biomimetic sensors, neuromorphic circuits, artificial neurons, and ionotronic devices demonstrate how ionic systems can achieve functionalities that electronic systems struggle to replicate.^10^ However, like traditional electronic systems that rely on metals and silicon semiconductors, artificial ionic systems require specialized conductors to support ionic conduction while maintaining the structural integrity.

The ionic conductors often contain solvents and are designed to be tough enough to ensure stable operation. Based on their conductor type, ionic systems can be broadly categorized as either liquid-type, or solid-type.^11^ Liquid-type ionic systems, widely used for their fluidic properties, require external vessels such as poly­(dimethylsiloxane) (PDMS), borosilicate glass, or polyurethane to maintain their shape. Gels, in contrast, composed of solvent and cross-linked polymer chains organized into a network structure, serve as self-standing solid state ionic conductors.^12^ Their high solvent content allows efficient ionic conduction, while their elasticity, mechanical resilience, self-healing property and biocompatibility make them attractive for flexible, stretchable and biointegrated applications (Figure 1b).^13,14^ Building on these advantages, gel-based ionic systems have emerged as a promising platform for integrating the conductivity of ionic solutions with the mechanical resistance of solid materials.

In this review, we explore both early developments and recent advances in gel-based ionic systems, categorizing them into four main groups: passive circuit elements, active circuit elements, power sources, and noncircuit elements (Figure 1c). Noncircuit elements refer to devices that do not conform to traditional circuit frameworks, enabling entirely new functionalities. (Figure 1d) We emphasize the distinctive attributes of gel-based ionic systems, including conductivity, operating mechanisms, and applications. Gel-based ionic systems are defined as those that rely on ionic conduction within gels, including materials such as hydrogels, ionogels, organogels, polyelectrolyte gels, and gel-like polymers with high solvent content within polymer networks. This definition excludes systems primarily based on electrochemical reactions, such as batteries, and solvent-free polymer networks, such as ionoelastomers.

## 2 Passive Ionic Circuit Elements

### 2.1 Ionic Resistor

#### 2.1.1 Ionic Conduction in Various Gel Electrolytes

When the attractive forces between the solvent and the solute ions exceed the interionic forces, the ions become solvated, meaning they are surrounded by solvent molecules.^15^ These solvated ions enable the conduction of electricity through their mobility, a process referred to as ionic conduction.^16^ In an aqueous solution, ionic conductivity (σ) is defined as shown in Equation 1, where *L* is the distance between the electrodes, *A* is the electrode area, and *R* is the measured resistance (Figure 2a). The total ionic conductivity is the sum of the contributions from the individual ionic conductivities of each ion species where *z*_*i*_ is the charge of ion species, *u*_*i*_ is the ion mobility, *c*_*i*_ is the ion concentration, and *F* is the Faraday constant.

1 $$\sigma =\frac{L}{R\cdot A}=\underset{i}{\sum }F\left|z_{i}\right|c_{i}u_{i}$$

**Figure 2:**
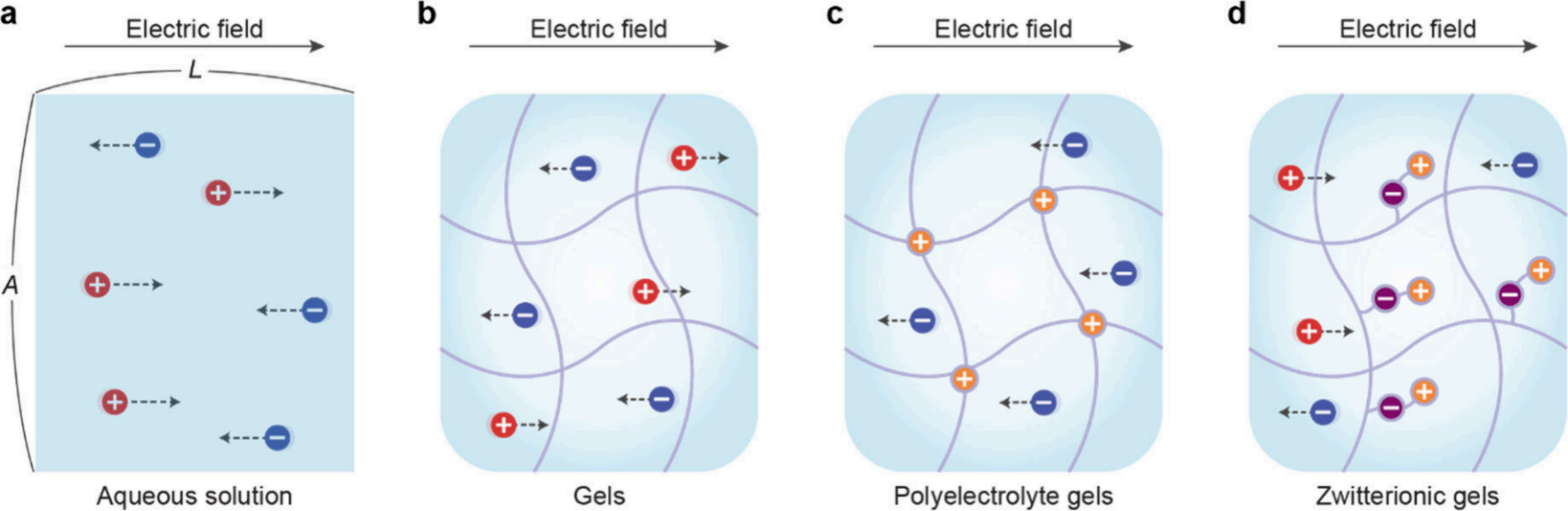
Ionic conduction in various matrices under external electrical fields. (a) Ion migration and ionic conductivity in aqueous solutions. (b) Ionic conduction in gels composed of polymer networks. (c) Selective ionic conduction in polyelectrolyte gels that have fixed charges in their polymer networks. (d) Ionic conduction in zwitterionic gels, where internal ion pairs facilitate charge transport while maintaining charge neutrality.

The ionic conductivity increases with higher charge, greater mobility, and higher ion concentration (up to a certain limit where ion–ion interactions do not hinder the mobility). Compared to electronic conduction, which is primarily governed by the movement of electrons, ionic conductivity is influenced by the type of ions, ion–ion interactions, and ion–solvent interactions. These interactions affect the mobility of ions, thereby influencing the overall ionic conductivity. Therefore, the mobility, *u*_*i*_, which represents the drift velocity (*v*_*d*_) of an ion under an electric field (*E*), can be further expressed using the Einstein relation, as follows:

2 $$u_{i}=\frac{v_{d}}{E}=\frac{\left|z_{i}\right|e}{6\pi \eta r}$$

The intrinsic viscosity of the solvent influences the ion mobility. However, strong ion–solvent interactions can also affect the overall viscosity (η) of the solution. Additionally, ion mobility is not solely dependent on the intrinsic size of the ion but also on the solvated radius (*r*), which is determined by the ion–solvent interactions in the electrolyte. In short, ionic conductivity is comprehensively governed by a combination of ion–ion and ion–solvent interactions, necessitating a comprehensive consideration of these effects.

Meanwhile, solid-like gels, which consist of a polymer network containing a large amount of solvent, facilitate ionic conduction by acting as a liquid electrolyte (Figure 2b).^17^ This similarity allows for predictable ion transport behavior, making neutral gels suitable as stable ionic conductors. Additionally, the unique properties of gels, such as softness, stretchability, biocompatibility, and transparency, significantly expand the versatility of ionic conductor applications.^18–22^ Furthermore, by tailoring the gel composition and structure, customized ionic conductors can be designed to meet specific application requirements.^23–25^ Hydrogels, composed mainly of water, offer excellent biocompatibility and high ionic conductivity, making them ideal for biomedical applications such as biosensors, drug delivery systems, and tissue engineering.^26–35^ Organogels, which incorporate organic solvents, provide enhanced chemical stability and lower volatility, making them suitable for applications in soft robotics and flexible electronics operating in nonaqueous environments.^36,37^ Ionogels, which contain ionic liquids (ILs) as the solvent, exhibit outstanding thermal stability and nonvolatility, making them highly promising for applications in energy storage systems and high-performance sensors.^38–43^

Certain features of the polymer network endow bulk gels with unique properties. Polyelectrolyte gels possess fixed charges along their backbone chain and mobile counterions (Figure 2c). Under an electric field, the migration of fixed charges is restricted, whereas mobile counterions migrate relatively freely. Additionally, the fixed charges in the polymer chain inhibit the movement of ions with the same charge while allowing for the transport of oppositely charged ions. This property enables the formation of cation- and anion-selective gels, also termed p-type and n-type polyelectrolyte gels. This selective ionic conduction through ionic interactions functions similarly to the rectifying behavior of a diode. Using polyelectrolyte gels, researchers have successfully developed active circuit elements such as ionic diodes and ionic transistors. A more detailed explanation about the electrical characteristics of polyelectrolyte gel will be discussed in Section 3.1. These properties of ion-selective polyelectrolyte gels are also utilized in various applications, including saltwater desalination,^44,45^ stimuli-responsive drug delivery devices,^27,46,47^ and ion-selective biosensors.^48–50^ Furthermore, many polyelectrolyte materials are hygroscopic and become stretchable and soft when sufficiently hydrated. Because of their highly polar networks, polyelectrolyte gels strongly interact with solvents, influencing the ion solubility and mobility. They also interact with additives, which can alter the mechanical and functional properties of the gels.^51–53^ Moreover, some polyelectrolyte gels exhibit self-healing properties, making them promising for various advanced applications.^54–57^

Zwitterionic gels, composed of monomers bearing both cationic and anionic groups, exhibit unique ionic conduction governed by internal ion pairs while maintaining overall charge neutrality (Figure 2d).^58^ This mechanism enables efficient ion transport with reduced ionic resistance and stable conductivity, even in high-salinity or complex ionic environments. Their balanced charge distribution minimizes ion aggregation, promoting smooth and selective ion migration.^59–61^ Additionally, zwitterionic gels demonstrate excellent hydration capacity, mechanical flexibility, and resistance to dehydration, which contribute to their long-term stability and durability.^62–64^ These properties make zwitterion-based gels promising candidates for applications in ion-selective membranes, solid-state electrolytes, and bioelectronic devices requiring controlled and stable ionic transport.

In addition to the intrinsic characteristics of ions and solvents, the polymer network plays a crucial role in determining the overall ionic conductivity of gels.^65,66^ Factors such as the degree of cross-linking, segmental mobility, and the polarity of the network influence the diffusion of ions by affecting ion–solvent interactions and the available free volume within the gel matrix.^15,67,68^ For instance, loosely cross-linked networks may offer lower tortuosity for ion migration, while polar functional groups on the polymer chains enhance solvation and dissociation of ions, leading to improved conductivity. Moreover, the mechanical elasticity of the polymer network has a significant impact on the functional reliability of gel-based ionic conductors, especially under deformation.^69–71^ Elastic and resilient networks maintain structural integrity and continuous ion pathways during stretching or bending, which are essential for applications requiring conformability and mechanical robustness. Recent strategies, such as the use of dual-network gels or zwitterionic systems further enable functionalities like strain-stiffening or self-healing, which contribute to long-term durability and stable ionic performance under repeated mechanical stress.^63,72,73^ This comprehensive understanding of ion transport and gel composition forms the basis for designing gel-based ionic resistors with tailored electrical and mechanical properties.

#### 2.1.2 Ionic Resistors with Advantages of Gels

By comprehensively considering the interactions among ions, the polymer network, and the solvent, ionic conductors can be strategically designed to achieve the desired properties. Tailoring the gel matrix, which serves as the medium for ionic conduction, allows us to develop ionic conductors that remain stretchable, soft, transparent, and self-healing even in ambiguous or dynamic environments. Furthermore, with the aid of advanced fabrication techniques such as three-dimensional (3D) printing, highly intricate and diverse ionic conductor structures can be realized with excellent processability.^74,75^

When a gel-based ionic conductor is connected to an electronic power source via a metal electrode, electrons attract ions, forming an electric double layer (EDL) at the interface between the gel and the metal electrode.^13^ The reactance of the EDL capacitor induces a phase shift between voltage and current, which can hinder accurate position detection in capacitive touch sensing systems. Kim et al. developed a highly stretchable and transparent ionic touch panel that also addresses this phenomenon (Figure 3a).^76^ A 2 M lithium chloride (LiCl) dissolved acrylamide (AAm) hydrogel touch strip is connected to Pt electrodes. Each Pt electrode is then connected to a current meter, which is subsequently linked to a single alternating current (AC) voltage supplier. When a finger touches the one-dimensional (1D) hydrogel strip, the hydrogel is divided into two resistors at the touching point. The resistance of each divided section is proportional to the distance from the touching point to the end of the hydrogel strip. Accordingly, the corresponding current flows from the voltage source to the grounded finger through each divided hydrogel strip. The reactance induced by the EDL capacitor at the interface between the hydrogel strip and the Pt electrode is significantly smaller than the resistance, which is modulated by the salt concentration. Consequently, since the phase angle is close to 0°, the impedance can be approximated by the resistance value. Therefore, based on this ionic mechanism, the touching position can be accurately determined from the ratio of the two divided resistances. Additionally, the soft and flexible nature of the hydrogel allows for seamless integration into wearable devices with simple encapsulation, ensuring conformability even under 1000% areal strain. Furthermore, as a transparent touch panel with ∼98% transmittance, it can be compatibly integrated with display devices. Beyond touch sensing, hydrogel-based ionic conductors have also been developed for wearable strain sensors, demonstrating their versatility across different applications.

**Figure 3:**
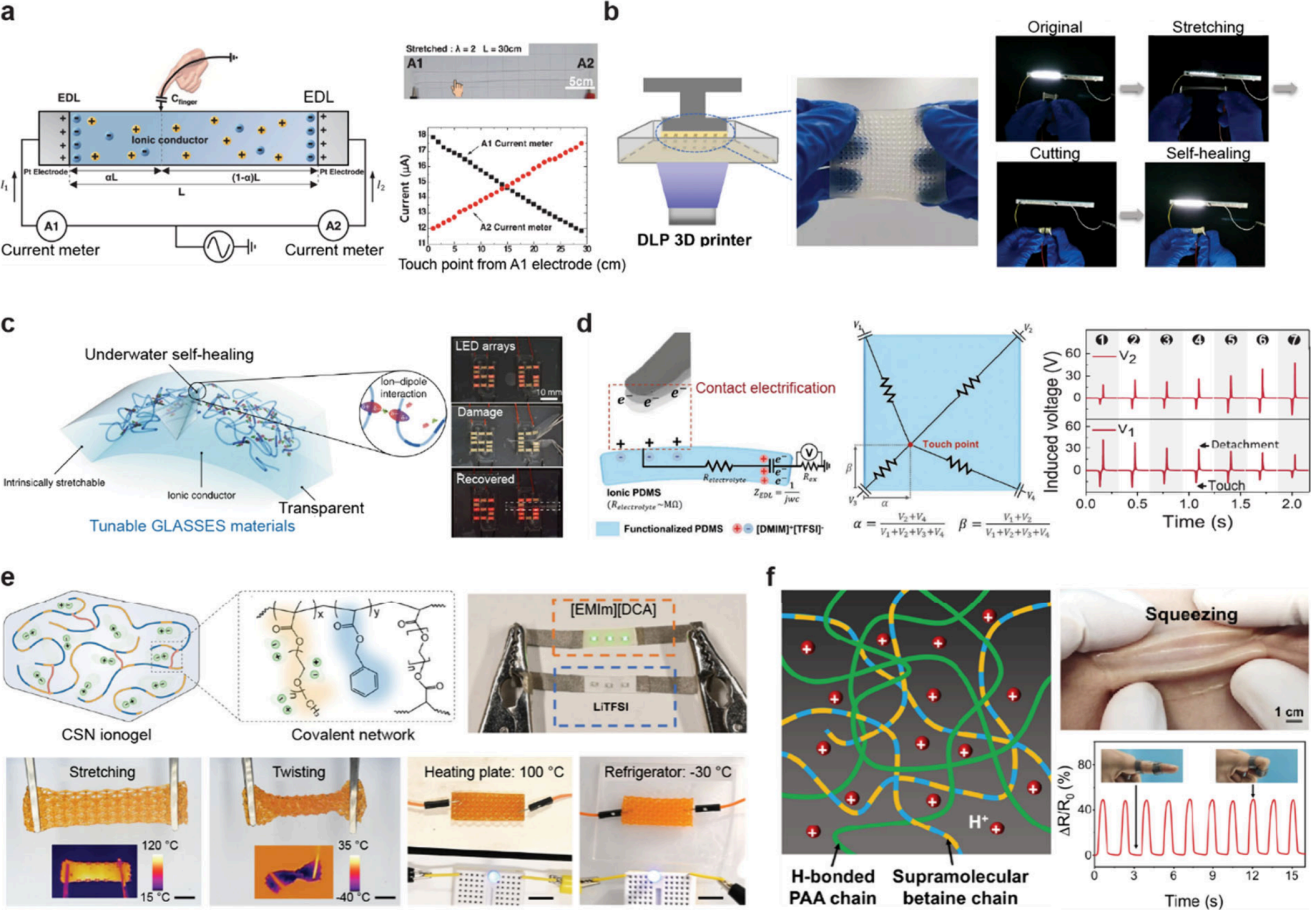
Various ionic conductors developed through the application of the diverse properties of gels. (a) A transparent and stretchable ionic hydrogel touch panel that senses position by measuring the current differences across each resistive part. Reproduced with permission from ref 76. Copyright 2016, The American Association for the Advancement of Science. (b) A hydrogel-based self-healing wearable strain sensor fabricated through 3D printing. Reproduced with permission from ref 77. Copyright 2023 Springer Nature under CC BY 4.0 http://creativecommons.org/licenses/by/4.0/. (c) A stretchable ionogel-based ionic skin with a self-healing property in underwater environments. Reproduced with permission from ref 73. Copyright 2019 Springer Nature. (d) An ionogel touch panel with triboresistive sensing for grid-free touch recognition. Reproduced with permission from ref 78. Copyright 2022 John Wiley and Sons. (e) Highly conductive and stretchable nanostructured ionogel fabricated through 3D printing with excellent ionic conductivity over a wide temperature range. Reproduced from ref 79. Copyright 2024 Springer Nature under CC BY-NC-ND 4.0 https://creativecommons.org/licenses/by-nc-nd/4.0/. (f) A zwitterionic hydrogel-based strain sensor with skin-like properties, featuring high stretchability (∼1600%) and strain-stiffening (∼24-fold modulus enhancement). Reproduced with permission from ref 72. Copyright 2021 Springer Nature under CC BY 4.0 https://creativecommons.org/licenses/by-nc-nd/4.0/.

A stretchable and self-healable hydrogel with a delicately designed structure, easily fabricated via 3D printing, is highly attractive as an ionic conductor for use in wearable devices. The processability and outstanding mechanical properties of hydrogels drive their widespread development in this field. Xiong et al. developed a material utilizing host–guest chemistry for self-assembly and photopolymerization to integrate hydrogels into wearable devices (Figure 3b).^77^ This material exhibits a high fatigue resistance and exceptional stretchability, allowing it to withstand frequent and ambiguous deformations encountered in daily life. By using the reliable resistance changes induced by the elongation of soft materials, an ionic conductor-based sensor was attached to the body to detect real-time biosignals from subtle muscle movements, such as swallowing, wrist bending, and pressing. Furthermore, by employing a photopolymerization-based 3D printing technique, the material can be synthesized into high-resolution and complex structures, enabling conformal application to various body parts. Additionally, the developed material exhibits self-healing properties, allowing it to be restored and reused as a sensor after damage with simple post-treatment.

Hydrogels exhibit significant variations in electrical and mechanical properties depending on their water content. In contrast to hydrogel based ionic conductors, the ionogel remains hydrophobic, preventing ion leakage and swelling. As a result, ionogels can serve as reliable and stable ionic conductors over extended periods, even under ambient conditions and in aqueous environments. Using these advantages of ionogels, a highly stretchable, transparent, and submersible ionic conductor was developed by combining a fluoroelastomer matrix with a fluorine-rich IL (Figure 3c).^73^ This unique composition enables tunable ionic conductivity (up to 10^–3^ S cm^–1^) through ion–dipole interactions, ensuring a stable electrical performance even in wet, acidic, and alkaline environments. The synergistic molecular design allows for autonomous electro-mechanical self-healing, where ion–dipole interactions restore conductivity and mechanical integrity upon damage. The material exhibits extreme stretchability (up to 2000% strain), high optical transparency (>98%), and environmental resilience, making it ideal for long-term underwater applications. This gel-based ionic conductor was successfully implemented in wearable biosensors, demonstrating real-time detection of touch, pressure, strain, and humidity. By integrating optoelectronic signal transmission, the material also supports underwater communication systems, mimicking bioluminescent jellyfish. This self-healing, transparent, and submersible ionic skin presents a groundbreaking approach for aquatic robotics.

By effectively mixing the IL and elastomer to precisely control conductivity, this system overcomes the structural limitations of conventional triboelectric nanogenerators (TENGs). Unlike conventional TENGs that rely on stacked bilayers, a new approach was attempted to achieve homogeneous monolayer integration, enabling efficient triboelectric energy harvesting within a single-layer structure.^78^ A monolayered ionic PDMS-based triboresistive touch sensor was developed, offering grid-free touch recognition without the need for external power sources (Figure 3d). This innovative approach eliminates the need for separate charge-generating and charge-collecting layers, simplifying the structure while maintaining high performance. By tuning of ionic conduction, a novel triboresistive sensing mechanism was introduced, enabling highly sensitive and precise touch-point detection through touch-induced electrical field variations. The ionically conductive PDMS exhibits high transparency (96.5%), extreme stretchability (539.1%), and resilience (99%), ensuring skin-conformal adhesion and mechanical durability for wearable applications. The self-powered mechanism allows continuous operation without batteries, while the triboresistive sensing method enables multidimensional detection, including touch position, orientation, and grip force. This design also facilitates interaction with robotic systems, musical instruments, and human-machine interfaces, offering a simplified, adaptable, and power-efficient alternative to conventional touch-sensing technologies.

Hydrogel-based ionic conductors have a limited operational temperature range due to dehydration issues. By utilization of the low vapor pressure of ILs, ionogels were developed, enabling the implementation of more stable ionic conductors that operate reliably over a wider temperature range. He et al. reported a highly conductive and stretchable nanostructured ionogel, employing a photopolymerization-induced microphase separation strategy to form interconnected ionic nanochannels within a cross-linked polymeric framework (Figure 3e).^79^ This design enables high ionic conductivity (>3 S m^–1^), extreme stretchability (>1500%), low hysteresis, and broad thermal stability (−72 to 250 °C). The 3D printability of this ionogel marks a key advancement, overcoming previous limitations in resolution and mechanical integrity associated with digital light processing (DLP) 3D printing. This bicontinuous nanostructure allows fabrication of complex microarchitectures with resolutions down to 5 μm, while retaining mechanical flexibility and conductivity. The ionogel was successfully integrated into capacitive sensors for real-time physiological monitoring (e.g., breathing, swallowing, and pulse detection). It was also implemented in robotic grippers that operate across extreme temperature ranges (−30 to 150 °C), detecting pressure and object interactions with high spatial resolution. Additionally, sensor arrays were fabricated for high-resolution pressure mapping.

Implementing a skin-like ionic conductor has long been a desirable goal in the field of stretchable electronics. However, achieving both excellent mechanical and electrical properties while simultaneously incorporating the strain-stiffening behavior of natural skin within a single material has been challenging. Recently, this issue has been addressed by utilizing a zwitterionic network, enabling the development of a skin-like ionic conductor with these combined properties (Figure 3f).^72^ The entropy-driven dual-network design mimics natural skin, achieving a balance between elasticity, self-healing, and strain-stiffening, which are traditionally conflicting properties in stretchable ionic conductors. This system retains only equilibrium moisture, ensuring stable ionic conductivity while maintaining excellent moisture-preserving and antifreezing properties. The weakly bound zwitterionic chains provide initial softness, while sequential fragmentation of these chains under stretching results in a 24-fold modulus increase (strain-stiffening effect), enabling mechanical compliance similar to that of natural skin. The material was successfully implemented in wearable iontronic sensors, detecting strain, temperature, and pressure changes in real time. Furthermore, it was integrated into capacitive sensors for human–machine interfacing, exhibiting high sensitivity and repeatability for physiological monitoring. These devices are designed by carefully tuning ion mobility, solvent composition, and polymer network parameters described in Section 2.1.1. In particular, achieving stable ionic conduction under mechanical deformation requires balancing the free volume for ion migration and solvent retention within the gel.

#### 2.1.3 Gel-Based Ionic Resistors with Unique Characteristics of Ions

To further expand their functionality, recent developments have focused on enabling dynamic and responsive behaviors in gel-based systems under external stimuli. Distinct ionic conductors have been extensively studied by exploiting the unique characteristics of ions (properties not typically observed in conventional electronic systems) such as stimuli-responsive behavior, mobility differences, and ion selectivity. These intrinsic ion-driven functionalities open new possibilities for ionic conductors with extraordinary performance. By exploiting these unique properties and behaviors, numerous ionic conductors with remarkable functionalities have been developed. Many of these functionalities stem directly from the unique transport mechanisms discussed in Section 2.1.1. Differences in ion mass, mobility, and solvation result in dynamic responses to external stimuli, such as temperature, light, or chemical inputs, enabling programmable ionic behavior not achievable in conventional electronic systems.

Inspired by biological sensing processes in nature, where ions function as signal carriers, research has been conducted to mimic natural sensing systems through stimuli-responsive changes in ionic conductivity. These ionic systems also exhibit characteristics of synaptic plasticity that are observed in biological systems. A stimuli-responsive ionic hydrogel with thermally tunable ionic conductivity was developed to emulate biological synaptic functions (Figure 4a).^80^ This system utilizes azobenzene-functionalized imidazole (AZIM) salts, whose ionic conductivity is dynamically modulated by near-infrared light-induced thermal disassembly. By integrating Fe_3_O_4_ nanoparticles, which convert near-infrared light into localized heat, the hydrogel enables noncontact, reversible control of ionic conduction by disrupting AZIM ion aggregation and increasing free ion concentration. Because the charge carriers in this system are ions, similar to those in biological synapses, the hydrogel can efficiently mimic synaptic behaviors, including excitatory post-synaptic potential, paired-pulse facilitation (PPF), and spike-rate-dependent plasticity. This tunable ionic conductivity enables applications in artificial synapses, neuromorphic computing, and bioelectronic interfaces. A proof-of-concept robotic hand demonstrated adaptive learning and memory functions, processing optical stimuli to autonomously regulate movement.^81^

**Figure 4:**
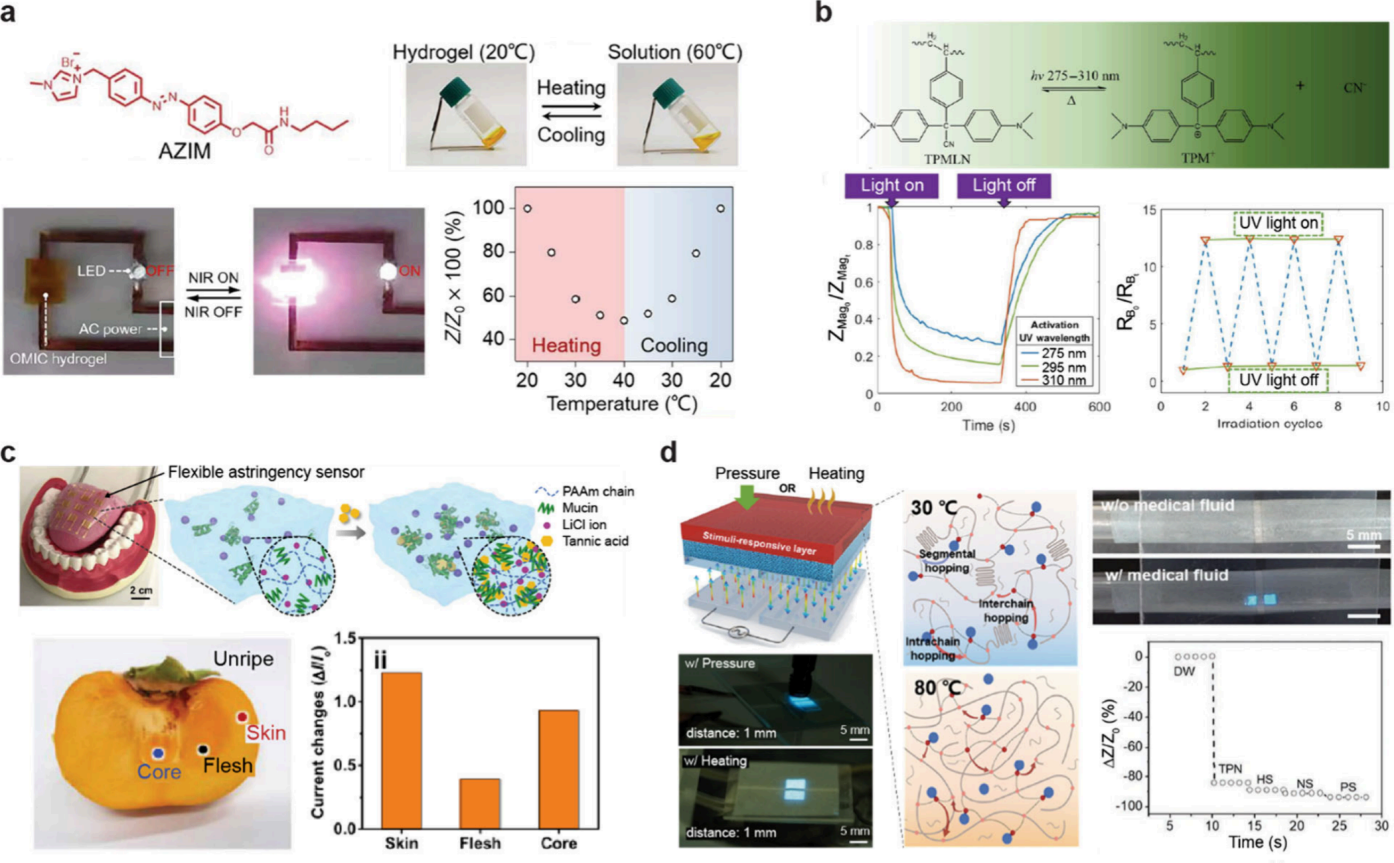
An ionic conductor harnessing the diverse properties and selectivity of ionic materials. (a) Ionic conductivity modulation through photothermally responsive AZIM ions for mimicking synaptic functions. Reproduced with permission from ref 80. Copyright 2023 The American Association for the Advancement of Science under CC BY 4.0 http://creativecommons.org/licenses/by/4.0/. (b) Optoionic hydrogels with UV-light-regulated ionic conductivity for ionic-based logic processing and image sensing. Reproduced with permission from ref 82. Copyright 2024 The American Association for the Advancement of Science under CC BY 4.0 http://creativecommons.org/licenses/by/4.0/. (c) An astringency sensing device that detects changes in ion conductivity induced by the degree of hydrophobic nanochannel formation. Reproduced with permission from ref 83. Copyright 2020 The American Association for the Advancement of Science under CC BY 4.0 http://creativecommons.org/licenses/by/4.0/. (d) Temperature-interactive display utilizing ionic conductivity changes driven by differences in ion diffusion rates due to the crystallization of the matrix at different temperatures. Reproduced with permission from ref 84. Copyright 2022 John Wiley and Sons.

Furthermore, a hydrogel-based artificial retina was fabricated by using a light-responsive ionic system to detect light intensity and reconstruct images, showcasing its potential for bioinspired vision systems. With photoactivated ion transport, this system bridges the gap between biological ion conduction and artificial computing. Chen et al. reported a UV-regulated optoionic hydrogel to achieve reprogrammable iontronics by actively modulating ionic conductivity through photoionization reactions (Figure 4b).^82^ This system incorporates triphenylmethane leuconitrile molecules, which undergo UV-induced photocleavage, generating cyanide anions with high mobility, while the counter cations remain attached to the polymer network. This mechanism allows for precise spatial and temporal control over ion transport, enabling a 10-fold increase in the local conductivity upon UV irradiation. The ionic conductor mimics biological processes and ensures efficient signal transmission in soft hydrated environments. This ionic system was successfully integrated into reprogrammable iontronic logic gates, where UV light was used to switch conductivity states, demonstrating AND, NOR, and NOT logic gate functions.

Human taste sensing also relies on ionic signals. Inspired by this, an artificial tongue was developed by utilizing the influence of the polymer network in hydrogels on the ion mobility to detect specific tastes. Yeom et al. proposed a soft and ion-conducting hydrogel-based artificial tongue to mimic human astringency perception through chemiresistive ionic conductivity changes (Figure 4c).^83^ The hydrogel consists of a pAAm network infused with mucin proteins and LiCl electrolytes, simulating the salivary environment of the human tongue. When exposed to astringent compounds such as tannic acid, the tannic acid molecules bind with mucin, forming hydrophobic aggregates that transform the microporous hydrogel into a micro/nanoporous structure, significantly enhancing ionic conductivity. This mechanism closely replicates the biological interaction between salivary proteins and astringents, leading to a rapid and sensitive response. The artificial tongue demonstrated a wide detection range (0.0005 to 1 wt % tannic acid), high sensitivity, and a fast response time (∼10 s). Additionally, the hydrogel-based sensor was used to monitor fruit ripening by detecting impedance changes associated with the polyphenol content. This study highlights the potential of chemiresistive ionic hydrogels in bioinspired sensory applications, particularly in artificial taste systems and food quality monitoring.

Similarly, research has been conducted on pressure- and thermal-sensitive displays, utilizing differences in ionic conductivity based on the degree of polymer crystallization within the gel. Jang et al. presents a wireless, stand-alone interactive display utilizing direct capacitive coupling to enable stimuli-responsive sensing and display functions (Figure 4d).^84^ The ionic compounds in the stimuli-responsive layer modulate impedance in response to pressure and temperature, altering the local electric field and enabling real-time sensing and visualization of external stimuli. The pressure-responsive layer employs ionic gel micropyramids, which deform under applied pressure, increasing contact area and reducing impedance. This transition enhances the vertical electric field, activating the electroluminescent display output. Similarly, the temperature-responsive layer, composed of a poly­(ethylene oxide) (PEO)/lithium bis­(trifluoromethanesulfonyl)­imide LiTFSI/poly­(ethylene glycol)­dimethyl ether composite, adjusts ionic conductivity with thermal changes, allowing accurate temperature sensing. Applications of this system include wireless medical monitoring, haptic feedback interfaces, and a trimodal smart braille display where AC-induced electroluminescence, sound, and tactile vibration enable intuitive user interactions.

These gel-based ionic resistors, distinct from conventional electronic resistors, utilize both the inherent properties of gels and the unique characteristics of ionic charge carriers. The soft and stretchable nature of gels enables conformal contact with curved or moving surfaces, while their transparency and biocompatibility facilitate integration into wearable or biointerfaced systems. At the same time, using ions as charge carriers introduces new functional possibilities that are difficult to realize with electrons, such as dynamic tunability, selectivity, and hysteresis. These features collectively allow gel-based ionic resistors to operate not only as passive circuit elements, but also as responsive, multifunctional components in soft electronics, bioelectronics, and neuromorphic devices.^76,81,82,85^

### 2.2 Ionic Capacitor

#### 2.2.1 Gel-Based Ionic Capacitors

A resistor (*R*) is ideally defined in a circumstance where the frequency of an electrical signal is not considered. However, since most electrical signals involve various ranges of frequencies with AC, the concept of a capacitor, another passive element of the circuit that accounts for the frequency of an electrical signal, is necessary.

A capacitor generally consists of two conductive plates separated by a dielectric (insulator). When an external voltage is applied, positive charges accumulate on one plate and negative charges accumulate on the other. The dielectric material prevents the direct flow of current, allowing the charges to remain on the plates. These accumulated charges enable the capacitor to store electric energy. The ability of a system to store electric charge per unit applied voltage is called capacitance (*C*), which, for parallel-plate capacitors, is typically given by Equation 3:

3 $$C=\varepsilon \frac{A}{d}$$

where ε is the permittivity of the dielectric (indicating how effectively the material blocks or accumulates electric fields), *A* is the area of each plate, and *d* is the distance between them. If a voltage *V* is applied to the capacitor, then the stored charge *Q* is expressed as *Q* = *CV*. During discharge, current flows through the circuit for a finite period, making capacitors useful for signal processing applications such as noise filtering,^86,87^ voltage smoothing,^88,89^ and waveform shaping.^90,91^

Meanwhile, a resistor exhibits a constant impedance, regardless of the frequency of the applied voltage. In contrast, when a capacitor with capacitance *C* is driven by an AC voltage, the resulting AC current *I* is determined by the following equation:

4 $$I=\frac{V}{X_{c}}$$

where *X*_*c*_, the capacitive reactance, represents the effective impedance of a capacitor in an AC circuit and is defined by the following equation:

5 $$X_{c}=\frac{1}{\omega C}=\frac{1}{2\pi \mathrm{fC}}$$

As shown in Equation 5, capacitive reactance, which opposes the flow of electric current, increases as the frequency of the electrical signal decreases. Consequently, at very low frequencies, the capacitive reactance becomes infinitely large, resulting in very high impedance. In contrast, at very high frequencies, the capacitive reactance approaches zero, leading to very low impedance. Thus, a capacitor is a frequency-dependent passive element, whose impedance is determined by the signal frequency.

In systems where ions serve as the main charge carrier, this passive element can be observed in so-called “ionic capacitors.” In this review, we define “ionic capacitors” as systems where ions, rather than electrons, serve as the primary charge storage medium by forming EDLs at the interfaces between ionic gels and electronic electrodes.^92,93^ The capacitive behavior is driven by ionic charge accumulation near the electrode interfaces, while electronic current flows in the external circuit. This distinguishes ionic capacitors from conventional electronic capacitors, where electrons are the sole charge carriers. Furthermore, all ionic capacitors discussed in this review operate under nonfaradaic conditions (i.e., in the absence of redox reactions), ensuring that the charge storage mechanism is electrostatic rather than electrochemical.

The most critical aspect of an ionic capacitor is EDL, which forms at interfaces between electrodes and ionic media (Figure 5a).^94,95^ At the electrode–electrolyte interface, when electrons (or positive charges) accumulate on the electrode surface, oppositely charged ions in the electrolyte arrange themselves to reach electrostatic equilibrium. This process produces an inner Helmholtz plane (IHP), where ions or molecules are adsorbed, and an outer Helmholtz plane (OHP), which includes counterions with their hydration shells. Further away from the electrode, the diffuse layer beyond the OHP gradually restores a uniform ion concentration, causing a change in electric potential. Collectively, these layers form the EDL, which governs the capacitance of ionic capacitors. Outside the EDL, defined here as the “bulk region”, the electrical potential approaches zero. Various models, such as the Stern model and the Gouy^96^–Chapman^97^ model, have been proposed to explain EDLs. However, the Gouy–Chapman–Stern model, which unifies the concepts of the IHP and the OHP, is now employed to interpret EDL behavior.

**Figure 5:**
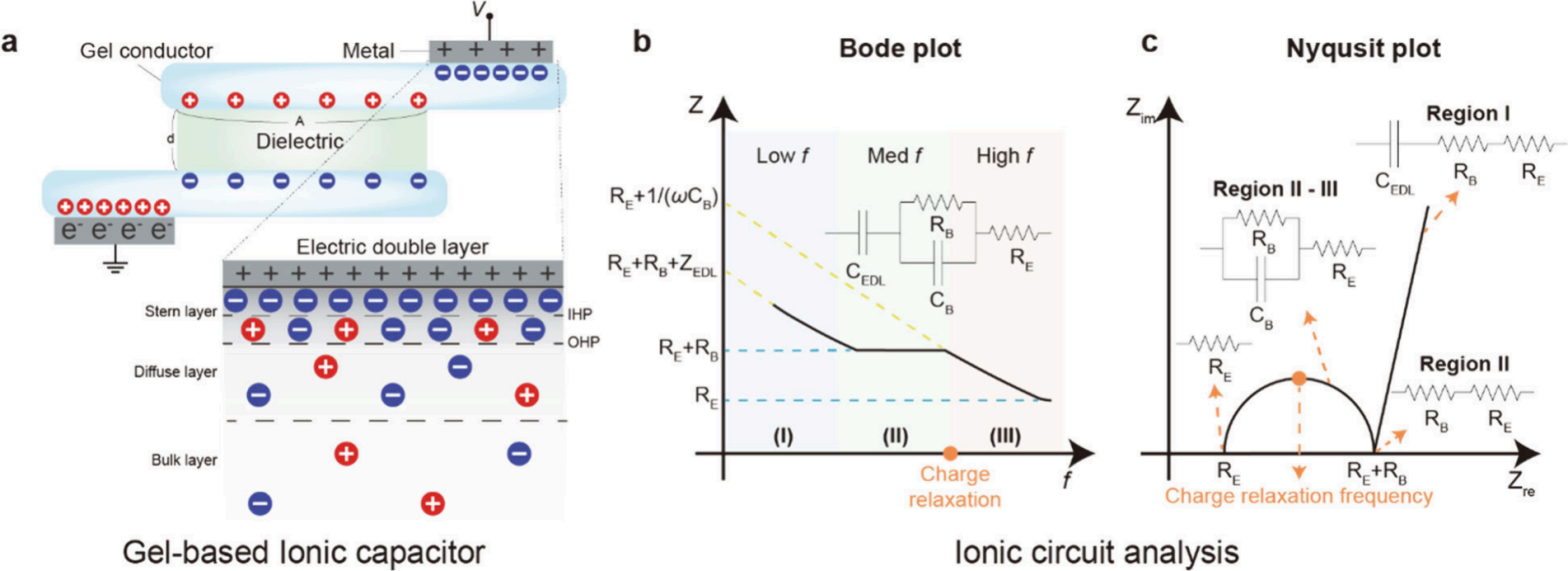
Gel-based ionic capacitors. Schematic diagrams of a gel-based ionic capacitor. (a) Ionic capacitor and the mechanism of EDL formation. When an electric field is applied to a gel conductor, the mobile ions within the gel behave similarly to electrons, thereby exhibiting capacitance. An EDL forms at the interface between the metal and the gel, effectively localizing the ions in place. Ionic circuit analysis of gel-based ionic capacitors. (b) Bode plot and (c) Nyquist plot of the ionic capacitor, highlighting its electrical performance. Reproduced with permission from ref 103. Copyright 2020 The American Association for the Advancement of Science.

Ionic capacitors exhibit several fundamental differences from conventional electron-based capacitors. When ions adhere closely to the electrode surface and form an EDL, the separated positive and negative charges interact across a gap on the order of just a few nanometers.^98–100^ This extremely short charge separation distance gives rise to an exceptionally high capacitance, often several orders of magnitude greater than that of traditional capacitors, constituting a key distinguishing feature of ionic systems. Therefore, the capacitance of ionic capacitors remains relatively high at low frequencies, where ions are free to move. In contrast, at high frequencies, ions cannot instantly follow the rapid changing of the electric field, causing ion relaxation and resulting in lower capacitance.

In order to analyze the AC impedance caused by such EDLs, it is necessary to examine both the Bode plot and the Nyquist plot. Figures 5b and 5c present the Bode and Nyquist plots of an ionic capacitor system, respectively. At the outset, we assume no faradaic (redox) reactions occur at the electrode–electrolyte interface, thereby omitting charge transfer resistance from consideration.

In the low-frequency region (Region I of Figure 5b, blue region), the bulk capacitance (*C*_*B*_) is much larger than the bulk resistance (*R*_*B*_), making the EDL capacitance (*C*_*EDL*_) the principal capacitance. Therefore, the electrical response in the low-frequency range is governed by the impedance of the EDL capacitance (*Z*_*EDL*_), with *R*_*B*_ and the electrode resistance (*R*_*E*_) added in series to contribute to the real (resistive) part of the impedance.

As the frequency increases, the imaginary component of the impedance contributed by the EDL capacitance decreases, reducing its influence and allowing the bulk impedance to become dominant. Consequently, in Region II (Figure 5b, green color region), the system can be modeled as a parallel RC circuit composed of *C*_*B*_ and *R*_*B*_. As shown in Figure 5b, the flat portion in Region II of the Bode plot corresponds to the real impedance (the sum of *R*_*B*_ and *R*_*E*_). Meanwhile, as seen in Figure 5c, Region II on the Nyquist plot begins at the point of intersection between the diagonal Warburg segment and the semicircle.

When the frequency increases further into the high-frequency range (Regions II–III), the influence of *C*_*B*_ becomes more pronounced as the imaginary component of the impedance continues to decrease. As shown in Figure 5b, the frequency at which the imaginary part of the impedance crosses the real part is the charge relaxation frequency. Because ionshave relatively lower mobility compared to electrons, they relax at lower frequencies. This low-frequency relaxation leads to hysteresis in electrical operation, a key characteristic of ion-based devices.

Consequently, Regions I and III, where polarization is dominant, are primarily determined by the real part of the impedance (*Z*_*re*_), whereas Region II, driven by ion transport, is characterized by a prominent imaginary part (*Z*_*im*_). Furthermore, in the very low-frequency region of the Nyquist plot, the linear segment corresponds to the Warburg impedance,^101,102^ which reflects ion diffusion rates in the gel matrix (Figure 5c).

#### 2.2.2 Ionic Capacitors with Gel-Based Dielectrics

The capacitance arising from the EDL, combined with the inherently superior mechanical properties of gels, has led to extensive research on diverse ionic capacitors. There are two types of ionic capacitors harnessing the gel’s properties. The first type is a system in which an ion-based gel acts as the dielectric in the capacitor (Figure 6a). The gel is a polymer structure containing a solvent, providing advantages such as transparency, stretchability, and flexibility and enabling a wide range of demonstrations. Although gels, which contain ions, are generally used as conductors, they can also serve as dielectrics when combined with an insulator. When a gel is used as the dielectric, its soft mechanical properties allow for mechanical freedom, enabling changes in the distance between the electrodes. Since capacitance varies with the distance between its electrodes, gel-based dielectric is frequently employed as a capacitive sensor.^104–108^ Various attempts have been made to build such sensors by converting these mechanical stimuli into changes in electrical signals.^109–113^ Wu et al. proposed a device in which a deformable gel is placed between two electrodes, allowing mechanical stimuli to be recognized as electrical signals (Figure 6b).^114^ Specifically, they positioned a rhombus-shaped gel composed of poly­(vinyl alcohol) (PVA) hydrogel and NaCl between the two electrodes. When a mechanical stimulus is applied, the gel is compressed, increasing the contact area with the electrodes. As a result, more ions come into contact with the electrodes, creating a potential difference between them. By exploiting the stretchability of the gel in a capacitor structure, the potentiometer can effectively detect not only static stimuli but also low-frequency dynamic stimuli with ultralow power consumption (less than 1 nW) and high tunability.

**Figure 6:**
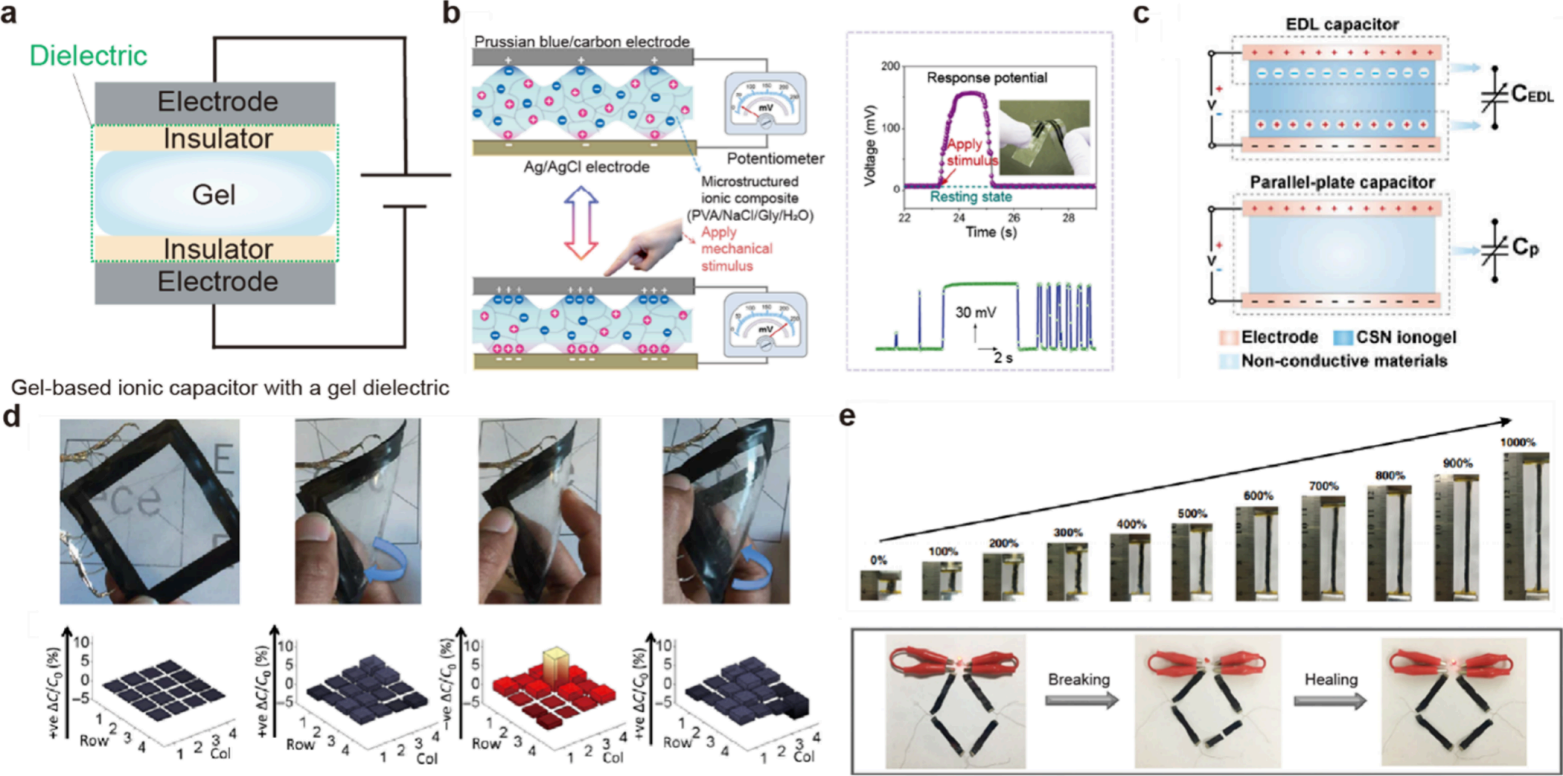
Applications of ionic capacitors using gel dielectrics. Gel-based ionic capacitors with gel dielectrics. (a) Schematic diagrams of a gel-based ionic capacitor incorporating a gel dielectric. (b) Thanks to the high compliance and transformability of gel, the movement of ions within gel changes under pressure, effectively enabling the device to function as a potentiometer. Reproduced with permission from ref 114. Copyright 2020 The American Association for the Advancement of Science. (c) By using the EDL, the capacitor dynamically adjusts its capacitance in response to pressure. Reproduced from ref 79. Copyright 2020 The American Association for the Advancement of Science under CC BY-NC 4.0 https://creativecommons.org/licenses/by-nc/4.0/. Constructed from soft materials, gel-based ionic capacitors can be deformed, demonstrating (d) flexibility, (e) stretchability, and self-healing properties. Reproduced with permission from ref 115. Copyright 2017 American Association for the Advancement of Science. Reproduced with permission from ref 116. Copyright 2019 Springer Nature under CC BY 4.0 http://creativecommons.org/licenses/by/4.0/.

As mentioned above, a key difference between electron-based capacitors and ionic capacitors is the presence or absence of an EDL. He et al. used a highly conductive and stretchable nanostructured ionogel to compare an EDL capacitor with a parallel-plate capacitor (Figure 6c).^79^ They showed that an EDL capacitor exhibits a much larger change in capacitance under applied pressure compared with a parallel-plate capacitor. The sensitivity of the EDL capacitor (*S* = (δΔ*C*/*C*_*0*_)/δ*P*) is about 0.23 kPa^–1^ within the pressure range of 1–5 kPa, which is significantly higher than that of the parallel-plate capacitive sensor (0.016 kPa^–1^). Additionally, while the parallel-plate sensor has low initial and maximum capacitances (*C*_*0*_ = 2.7 pF, *C*_*max*_ = 4.1 pF), the EDL capacitor with 40 wt % 1-ethyl-3-methyl-imidazolium dicyanamide ([EMIM]­[DCA]) exhibits ultrahigh capacitance (*C*_*0*_ = 36.8 nF, *C*_*max*_ = 329.7 nF). Furthermore, the *C*_*0*_ and *C*_*max*_ of the capacitive sensor increase with higher [EMIM]­[DCA] content since more IL moieties lead to more electron–ion pairs at the ionogel–electrode interface. Lastly, they demonstrated that this gel-based EDL capacitor could be used in wearable devices, thanks to its stretchability and self-healing properties.

One of the most prominent features of these gel-based ionic capacitors is their mechanical properties, such as flexibility and stretchability. Sarwar et al. leveraged these mechanical properties of gels to develop a bendable, stretchable touch sensor (Figure 6d).^115^ They used PDMS, widely used silicone elastomers, as the encapsulation layer for the dielectric combined with pAAm hydrogel. Since both PDMS and pAAm are bendable, stretchable, and optically transparent, the touch panel maintains functionality and remains transparent even under mechanical deformation. By applying a projected electric field, they enabled the sensor to detect a finger without direct contact, and by using mutual capacitance, they could differentiate between touch and bending.

Li et al. maximized the mechanical properties of gel-based capacitors by proposing an ultrastretchable supercapacitor (Figure 6e).^116^ Through double cross-linking of Laponite and graphene oxide, they developed a material that can stretch up to 1200%. They deposited carbon nanotube (CNT) films onto the prestrained gel, thus forming a wrinkled structure that could extend beyond 1000%. Benefiting from its wrinkled design, the device showed practically no change in capacitance under strains of up to 900%. Additionally, they took advantage of the gel’s self-healing property to further increase the device’s reliability. Since the hydrogel structure relies on physical cross-linking, applying a small amount of heat reforms the broken cross-links and restores the mechanical properties of the material, allowing the device to regain its original performance.

#### 2.2.3 Ionic Capacitors with Gel-Based Conductors

The second approach employs the gel as an ionic conductor (Figure 7a). As mentioned in Section 2.1.1, a gel is a material that can facilitate the movement of ions through a solvent, enabling it to function as a conductor by means of ion migration. As the gel itself serves as a conductor, its transparency, stretchability, and flexibility enable a wide variety of demonstrations.^117–124^

**Figure 7:**
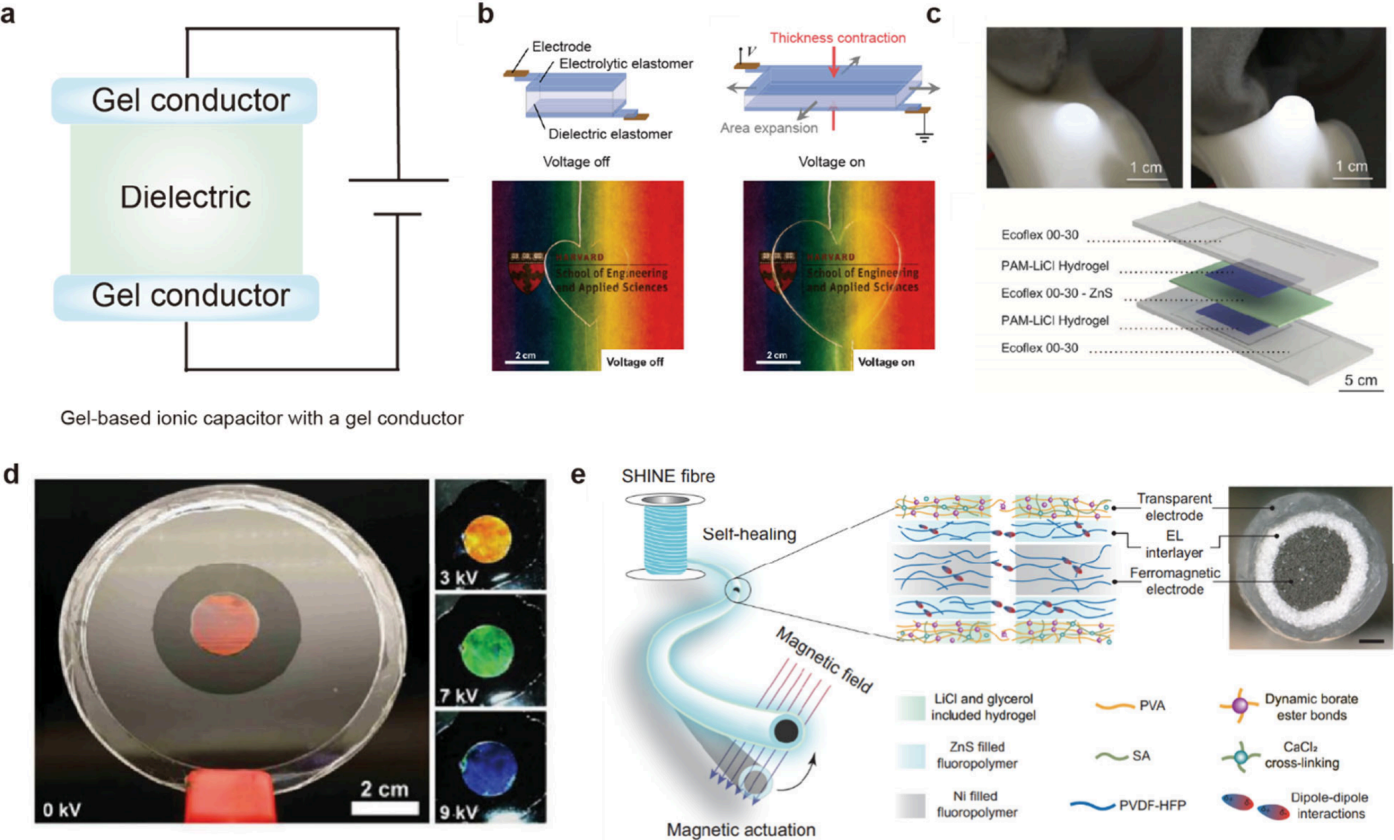
Applications of ionic capacitors using gel conductors. Gel-based ionic capacitors using gel conductors. (a) A schematic diagram of gel-based ionic capacitor incorporating a gel conductor. (b) By employing a gel as a reservoir for mobile ions, stretchable and transparent conductors can be developed. Reproduced with permission from ref 117. Copyright 2013 The American Association for the Advancement of Science. (c) Owing to the transparency of the gel conductor, ACEL can be demonstrated. Reproduced with permission from ref 135. Copyright 2016 American Association for the Advancement of Science. (d) When used in a DEA with photonic crystal gel, the gel conductor can display various colors with electrical signals. Reproduced with permission from ref 146. Copyright 2018 John Wiley and Sons. (e) By simultaneously cross-linking the gel conductor and the electroluminescent layer, a fiber-shaped display can be fabricated. Reproduced from ref 154. Copyright 2024 Springer Nature under CC BY-NC-ND 4.0. http://creativecommons.org/licenses/by-nc-nd/4.0/.

Keplinger et al. were the first to demonstrate the gel’s significant potential by showing its utility as a stretchable, transparent ionic conductor (Figure 7b).^117^ In their study, they explain that in a capacitor structure where a separate dielectric layer exists the voltage drop across the dielectric is much larger than that caused by the EDL. Consequently, despite the presence of the EDL, the gel still functions effectively as the conductor. They demonstrated this principle by applying the gel as the electrode in a dielectric elastomer actuator (DEA), which is a type of actuator that uses electrical signals to induce mechanical motion.^125–134^ When an electric field is applied to this capacitor-like structure, charges accumulate and create electrostatic forces, resulting in “Maxwell stress.” In a DEA, an elastomer used as the dielectric responds to this stress with a reduced thickness and increased area. The use of a hydrogel electrode in a DEAenabled the development of a transparent DEA device, further showing its feasibility as a high-frequency actuator capable of serving as a loudspeaker.

By facilitating the transparency of gel conductors, the demonstration of an electroluminescence-based display can be achieved. Larson et al. used the transparent and stretchable nature of gels to develop an electroluminescent skin capable of both optical signaling and tactile sensing (Figure 7c).^135^ By creating a hyperelastic light emitting capacitor composed of a dielectric layer (Ecoflex and ZnS phosphors) and an electrode layer (pAAm hydrogel infused with LiCl), they employed alternating current electroluminescent (ACEL) technology. In ACEL, an alternating voltage is applied to a phosphor-based light-emitting material.^135–142^ Within a capacitor structure, phosphor molecules or ions are alternately accelerated by the electric field, gain energy, and then emit photons as they return to the ground state.Owing to the unique mechanical and optical properties of gel-based capacitors, this work realized the first stretchable and soft display, capable of sustaining strains up to 549% while remaining easily deformable.

When a gel, which has good mechanical stability and stretchability, is used as the matrix for a photonic crystal, it can change color through the mechanical deformation, demonstrating its potential value as a color-tunable electrode.^143–145^ Kim et al. employed a photonic crystal gel electrode, whose reflected wavelength changes under mechanical stretching. Combined with a DEA structure, the system enabled a display that can produce both color changes and sound (Figure 7d).^146^ Photonic crystals are periodic structures of two materials with different refractive indices, reflecting a specific wavelength via Bragg’s diffraction.^143,147–153^ Kim et al. built a superlattice with uniformly sized nanoparticles designed to reflect visible light and embedded it in a gel. The color changes when the particles’ spacing is physically altered, making photonic crystals and stretchable gels a frequently used combination for controllable color shifts. Since the gel can be physically stretched, the interparticle distance in the superlattice changes in a stable manner, resulting in a shift of the reflected color. They developed a display whose color can be altered by electrical signals. Moreover, because this display is DEA-based, it achieves very high actuation speeds and can generate sound when driven by an AC signal. By controlling both AC and direct current (DC) signals, they demonstrated a new display concept that stimulates two human senses simultaneously by managing sound and color output in the same device.

By capitalizing on the hydrogel’s processability, transparency, and mechanical stability, the gel conductors show considerable potential for applications in wearable devices, healthcare, and beyond. Fu et al. harnessed the self-healing property, transparency, and processability of hydrogels to develop a self-healing and actuatable fiber (Figure 7e).^154^ By employing a transparent gel electrode as the foundation for an ACEL fiber, they addressed a critical shortcoming of conventional integrated electronic fibers and electroluminescent devices, which frequently experience severe performance degradation upon damage. Specifically, they utilized a hydrogel in both the electroluminescent layer and the transparent electrode. This design enabled the device to autonomously self-heal and recover up to 98.6% of its initial luminance, maintaining a stable performance for over 10 months. Moreover, the proposed fiber-type electroluminescent device demonstrated a record luminance of 1068 cd/m^2^ at an electric field of 5.7 V/μm. Through coaxial wet-spinning and ion-induced gelation, they successfully mass-produced this high-performance fiber up to 5.5 m in length. Additionally, incorporating a Ni core provided magnetic actuation capability, allowing the fiber to bend freely under an external magnetic field without requiring a separate actuator.

#### 2.2.4 Ionic Capacitors with Unique Characteristics of Ions

Ionic capacitors offer enhanced functionality due to the unique characteristics of the ions. The EDL formed between the ion and the electrode provides a notable characteristic of an ionic capacitor. The capacitance generated by the EDL is extremely high due to the very short distance between the electrode surface and the ion layer, typically on the order of a few nanometers. Consequently, this capacitor can achieve a capacitance larger than that of conventional capacitors, and such capacitors are referred to as supercapacitors. Unlike electrons, which relax quickly when the electric field is removed, ions have a slower relaxation time, enabling them to maintain capacitance for a longer duration. This feature enhances both the storage capacity and the retention time of a capacitor, which is traditionally used as a passive storage element. Wang et al. demonstrated a flexible supercapacitor using chemically cross-linked hydrogel (Figure 8a).^155^ They constructed a capacitor structure by employing PVA and H_2_SO_4_ hydrogel as the dielectric, with glutaraldehyde as a cross-linking reagent, and polyaniline as the electrode. This supercapacitor is capable of 300% stretching, exhibits flexibility, and demonstrates a capacitance of 488 mF/cm^2^. It also shows good cyclic stability and mechanical durability, suggesting its potential as a next-generation power source.

**Figure 8:**
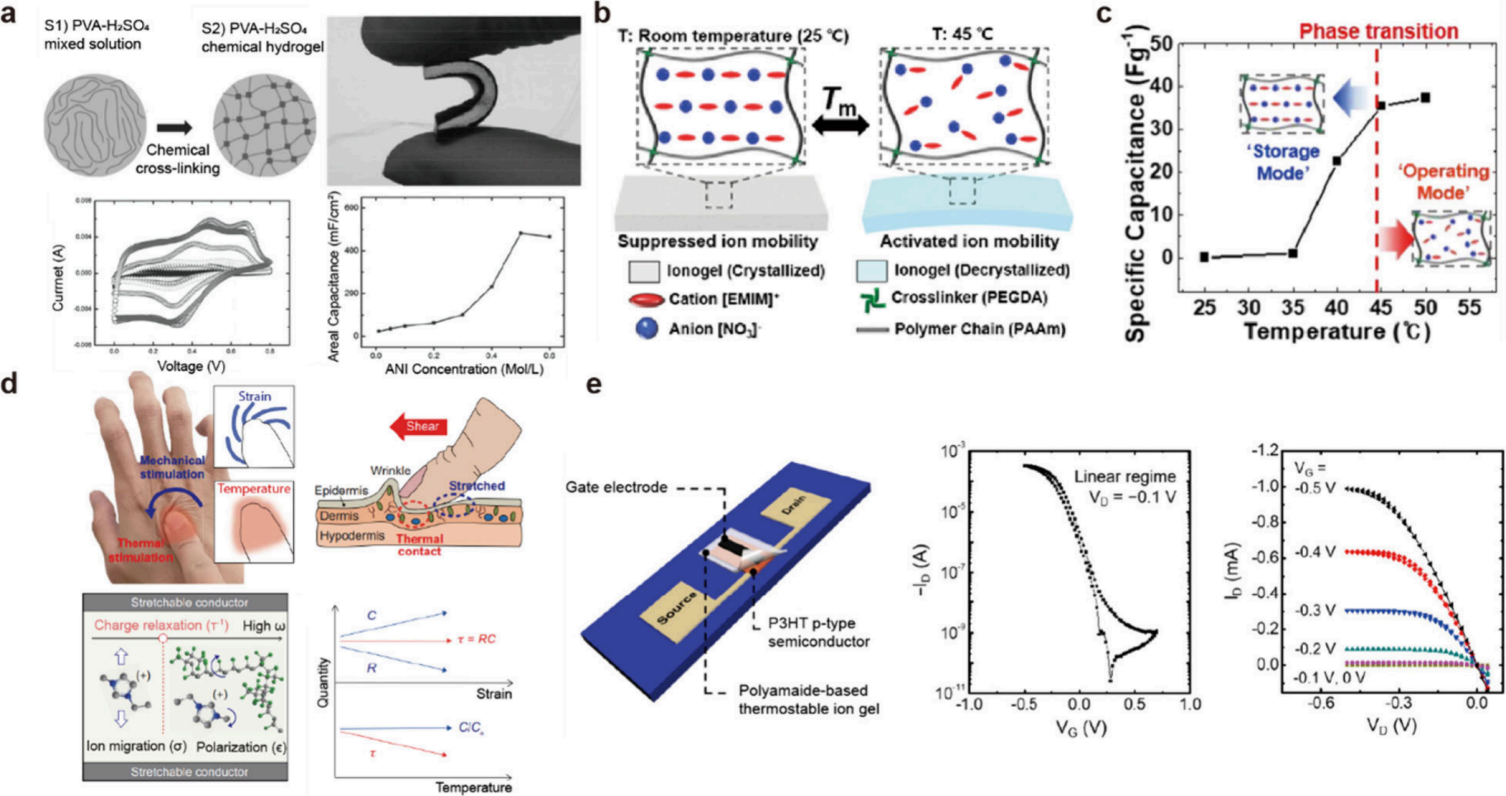
Applications of ionic capacitor using ion’s properties. By using ions as the electrical carriers, a distinctively functional ionic capacitor can be achieved. (a) Owing to the slow relaxation of ions, which facilitates the formation of the EDL in ionic capacitors, supercapacitors with higher capacitance and prolonged energy storage capabilities can be achieved. Reproduced with permission from ref 155. Copyright 2015 John Wiley and Sons. (b,c) By utilizing an IL and its sol–gel transition, a supercapacitor is proposed that can switch between storage mode and operation mode. Reproduced from ref 159. Copyright 2022 American Chemical Society under CC BY-NC-ND 4.0. http://creativecommons.org/licenses/by-nc-nd/4.0/. (d) By harnessing the inherent properties of ions—namely, that ion migration dominates at low frequencies while polarization dominates at high frequencies—a sensor is demonstrated that uses ionic relaxation dynamics to simultaneously detect mechanical signals and temperature. Reproduced with permission from ref 103. Copyright 2020 The American Association for the Advancement of Science. (e) By manipulating the transistor gate’s capacitance through dynamic ion migration and a gel, the device achieves a high on/off ratio (>10^5^) at low voltages (<1 V) and operates stably even at high temperatures (150 °C). Reproduced with permission from ref 167. Copyright 2020 American Chemical Society.

Motivated by the storage-element capabilities of such supercapacitors, various research groups have continued investigations in this area.^116,156–158^ Park et al. presented a phase transitional supercapacitor using an IL-based ionogel (Figure 8b,c), achieving selective operation through two modes: storage mode and operation mode. By using an IL as the main charge carrier that can crystallize when temperature changes, this device improves stability as a storage element.^159^ Traditional supercapacitors that use ions tend to relax relatively quickly when exposed to an electric field (i.e., they remian in an active state), making extended energy storage challenging. Park et al. proposed an ionogel composed of [EMIM]^+^[NO_3_]^−^ that can reversibly transition between a crystalline phase and an amorphous phase. In the amorphous phase, an electric field is applied to generate capacitance. Lowering the temperature then induces the crystalline phase, fixing the ions in place to preserve that capacitance. This crystalline-phase approach suppresses the degree of self-discharge, enabling storage of 89.51% of the charge even after 24 h, thus contributing to the development of next-generation storage devices.

In addition to serving as a storage element, capacitors have also been widely researched for their potential as sensors that detect changes in the capacitance. Gel-based sensors utilize the mechanism of detecting shifts in capacitance induced by physical changes. To measure pressure, contact, deformation, and temperature, multiple types of sensors would normally be required. However, human skin can recognize changes in both temperature and deformation separately owing to its use of ionic substances and multilayer structures. You et al. presented a sensor capable of detecting both temperature and mechanical deformation by leveraging ion relaxation dynamics (Figure 8d).^103^ The charge relaxation time of ions, which is defined as the ratio of permittivity to ionic conductivity, remains relatively unaffected by mechanical deformation. However, changes in temperature alter ionic conductivity and permittivity, so variations in the charge relaxation time can be used to sense the temperature. Additionally, as in a conventional capacitor-based sensor, the sensor can measure changes in capacitance induced by physical deformation, such as pressure or contact. Notably, when an AC electrical signal is applied to the ionic electrolyte in a capacitor structure, ion migration dominates at low frequencies, while polarization becomes dominant at high frequencies. Thus, in the high-frequency region, the capacitance due to polarization is more sensitive to physical changes, allowing the sensing of deformation. In the low-frequency region, by contrast, the ion relaxation time becomes more sensitive due to ion migration. This allows the temperature to be measured without interference between the two signals, thereby enhancing sensor accuracy.

As will be discussed in more detail in Section 3.2, transistors are the most representative components of semiconductor technology, capable of amplifying signals.^160–162^ Among such devices, electrolyte-gated transistors, which incorporate electrolyte in the gate, utilize dynamic ion movement and polarization effects to produce I–V curves or capacitances that depend on time and voltage history.^163–165^ These offer advantages such as low operating voltage, high output current, and low power consumption. In particular, since the systems utilizing ions create dynamic capacitance through ion dynamics, they can serve in various transistor modes.^166^ Cho et al. demonstrated an electrolyte-gated transistors employing gel-type electrodes made of semicrystalline polyamides and an IL (Figure 8e).^167^ Due to the high melting temperature of polyamides, they can function as gate dielectrics that operate above approximately 150 °C. Since ions act as carriers, the EDL formed at the electrode interface exhibits a very high specific capacitance (10.5 μF/cm^2^). By using this gel dielectric, the transistor operates at low voltages (<1 V) and achieves a high on/off ratio (>10^5^). This work illustrates the potential for expanding the use of ion-based devices.

In short, we examined the fundamental principles and distinctive characteristics of ionic capacitors. The formation of EDLs at the electrode and electrolyte interface enables exceptionally high capacitance, giving rise to superior energy storage capabilities compared with conventional electron-based capacitors. Additionally, the relatively slow relaxation dynamics of ions within gel matrices allows for stable charge retention over time. While the inherently slower mobility of ions leads to higher internal resistance and limits high-frequency performance, ionic capacitors offer rapid charge–discharge responses and high energy density in the low-to-mid frequency range, making them attractive for power-dense energy storage applications, which is called supercapacitor.^156,168–172^

In addition to their energy storage role, ionic capacitors have also been utilized as sensors that respond to external stimuli by modulating their capacitance.^173–183^ As discussed earlier, variations in the distances between the electrodes directly affect EDL formation, thereby altering the capacitance. Combining on the mechanical deformability of soft materials, such as hydrogels, researchers have developed sensors whose capacitance can dynamically change in response to mechanical,^184,185^ thermal,^186,187^ or chemical^188,189^ inputs. Moreover, while ions serve as the primary charge carriers, ionic sensors have also been developed that minimize the contribution of the EDL through internal circuit design and frequency control.^118^ Recent advances in soft-material fabrication have enabled the development of coplanar capacitor geometries, beyond traditional parallel-plate configurations, broadening the applicability of ionic capacitors as versatile and spatially resolved sensing platforms.^190,191^

### 2.3 Ionic Memristor

#### 2.3.1 Memristors and Memristive Systems

In 1965, Gordon E. Moore published an article proposing Moore’s Law, which states that the number of transistors integrated into a semiconductor doubles approximately every year.^192–194^ However, as semiconductor fabrication reaches physical limits, challenges such as cooling, cost-effectiveness, and quantum mechanical effects have slowed the advances in integration density. Moreover, the fundamental bottleneck of the Von Neumann architecture, which consists of a central processing unit (CPU), memory, and programs, necessitates a completely new design strategy.^195,196^

Recently, neuromorphic computing has emerged as an alternative to overcome the limitations of conventional computing architectures by mimicking the neurons (processing units) and synapses (memory units) structures of biological brains.^197,198^ For synapse-mimicking devices to function effectively, they must exhibit nonvolatility, multilevel conductance states, and adaptive behaviors.^199^ These devices should also be able to modulate their resistance based on the applied voltage while retaining this state for a given duration. A component that satisfies these properties is known as a memristor, a combination of memory and resistor.^200^

The concept of the memristor was first theoretically introduced in 1971 by electrical and computer engineer Leon Chua.^200^ Chua hypothesized the existence of a fourth passive element, the memristor, which connects the electric charge and magnetic flux (Figure 9a). Unlike other three linear time-invariant elements, the memristor operates as a dynamic element with memory-dependent functionality. According to him, the electrical resistance of a memristor is not constant but varies based on the direction and magnitude of historical current flow previously passed through it.^200,201^ He also analyzed “memristive systems” from the perspective of energy storage and measurable electrical properties, generalizing the concept of the memristor.^202^ An ideal nonvolatile memristor maintains its resistance state indefinitely without an external electric field, allowing discrete levels of electrical resistance to be retained over time.^203^ Additionally, a periodic pinched hysteresis loop in current–voltage (*I*–*V*) curves is a defining characteristic of memristors.^204,205^ In this Perspective, we discuss not only ideal memristors but also various studies that can be classified as memristive systems.

**Figure 9:**
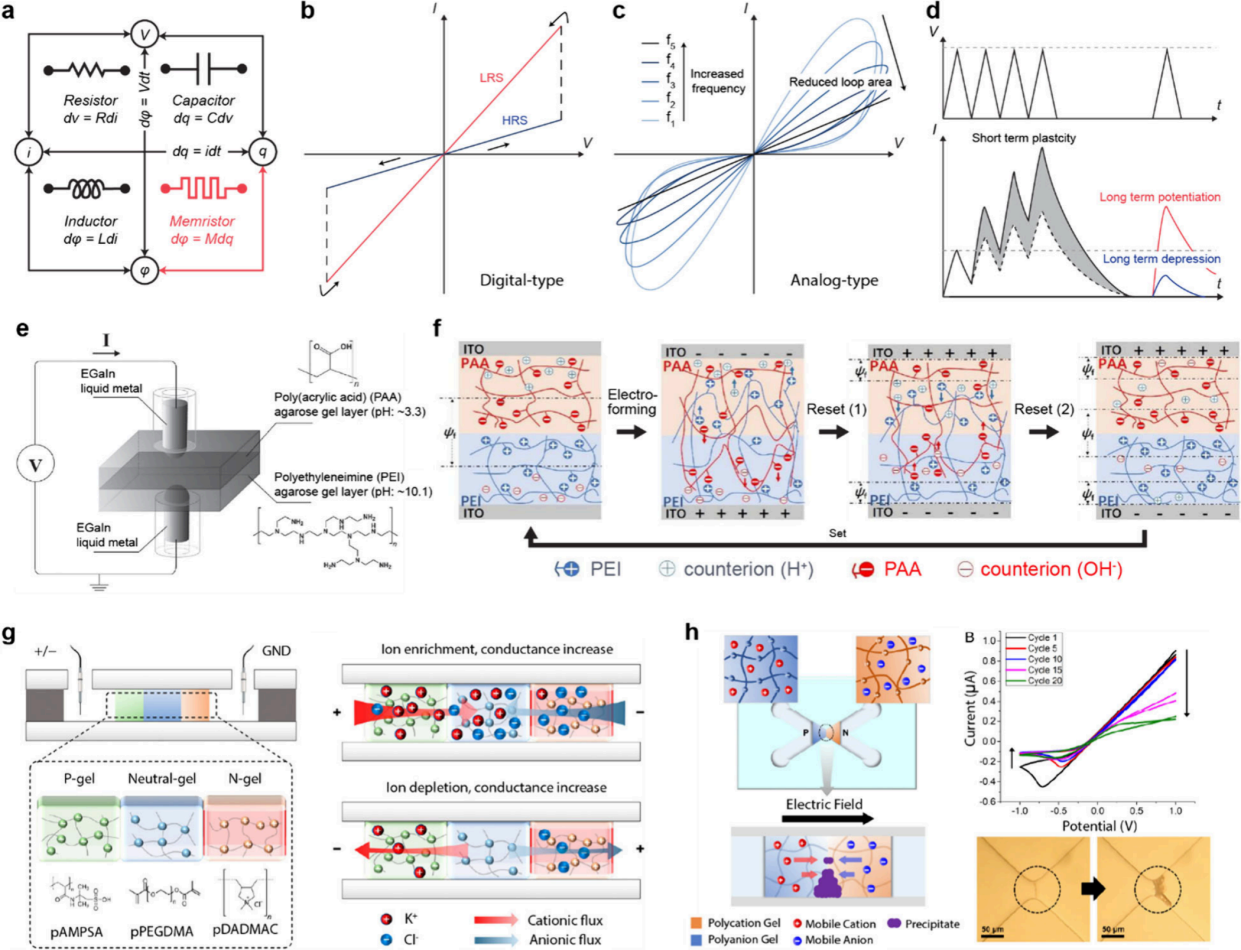
Electrical characteristics of ionic memristors and gel-based memristive devices. (a) The four fundamental passive circuit elements: resistor, capacitor, inductor, and memristor. The functional relationship of Memristance (*M*) = dφ = *M*d*q* = *v/i*, but its value is dynamically determined by the history of charge accumulation and depletion over time. (b) Current (*I*)–voltage (*V*) curves of the digital-type memristors. Two discrete types of resistive switching between high-resistance state and low-resistance state are shown. (c) *I–V* curves of the analog-type memristors under various frequencies. The loop area of pinched hysteresis curves decreases as the frequencies are increased. (d) Schematic depiction of synaptic plasticity: short-term plasticity, long-term potentiation, and long-term depression. (e) Soft memristor based on a polyelectrolyte gel and liquid metal. The formation and modulation of oxide layer at the gel/liquid metal interface show memristive behavior. Reproduced with permission from ref 217. Copyright 2011 John Wiley and Sons. (f) Polyelectrolyte gel/ITO electrode-based memristor. The migration of polyelectrolyte chains and counterions under an applied bias exhibited synaptic plasticity. Reproduced with permission from ref 218. Copyright 2022 American Chemical Society. (g) Bipolar polyelectrolyte hydrogel-based iontronic memristors. The electroneutral gel layer between the oppositely charged polyelectrolyte gels facilitates the effective modulation of ion transport and conductance. Reproduced with permission from ref 219. Copyright 2024 American Chemical Society. (h) Iontronic analogue of synaptic plasticity via reversible chemical precipitation and dissolution. Reproduced with permission from ref 220. Copyright 2022 the National Academy of Sciences under CC BY- NC-ND 4.0 https://creativecommons.org/licenses/by-nc-nd/4.0/.

Although first theorized in 1971, memristors were experimentally demonstrated 37 years later, in 2008, by HP (Hewlett-Packard) Laboratories.^206^ This breakthrough led to the development of various solid-state digital-type memristors that toggle between high-resistance states and low-resistance states, analogous to the “0” and “1” states in traditional binary data storage for computers (Figure 9b).^207^ For example, phase change memristor,^208,209^ resistive random access memory,^210,211^ ferroelectric memristor,^212^ and diffusive memristor^213,214^ are representative digital-type memristors that regulate electronic conductance via metals or metal ions. These systems exhibit not only resistive switching but also neuromorphic functionalities. However, when integrating these systems with biological systems, challenges inevitably arise due to the mismatch between electron- and ion-based language.

To bridge this gap, ionic and analogue-type memristors have been explored as potential solutions. These systems exhibit a pinched hysteresis loop that shrinks with increasing signal frequency and eventually collapses into a straight line at infinite frequency (Figure 9c). This behavior represents the fingerprint characteristic of a memristor, as originally defined by Chua.^215^ Compared with electronic memristors, ionic memristors more closely resemble biological systems in terms of both charge carriers and adaptive behavior. They exhibit gradual modulation of conductance, which mimics the continuous and graded nature of synaptic weight changes in biological systems. This enables them to emulate synaptic plasticity, a fundamental mechanism for information storage in the brain.^216^

Synaptic plasticity can be generally categorized into short-term plasticity (STP), which involves transient responses that decay rapidly, and long-term plasticity, which leads to persistent changes in synaptic efficacy. STP includes phenomena such as short-term facilitation and short-term depression (STD), which transiently enhance or suppress synaptic transmission in response to closely spaced stimuli. Long-term plasticity is further divided into long-term potentiation (LTP), which strengthens synaptic connections, and long-term depression (LTD), which weakens them (Figure 9d). These biological phenomena can be functionally emulated by ionic memristors, which modify their conductance through transient local ion accumulation and sustained ionic reconfiguration. Additionally, memristors can mimic various key features of synaptic functions, including PPF, paired-pulse depression (PPD), spike-rate-dependent plasticity (SRDP), and spike-timing-dependent plasticity (STDP).^207^ This functional correspondence highlights the potential of ionic memristors as hardware analogs of biological synapses in neuromorphic computing systems.

In the following section, we discuss gel-based ionic memristors, emphasizing their mechanical and electrical properties as well as their synaptic plasticity. We will explore their material designs, working mechanisms, and neuromorphic functionalities, covering their progression from early concepts to the latest advancements.

#### 2.3.2 Gel-Based Ionic Memristive Systems

Ionic memristors utilize dynamic ion transport behavior to generate various nonlinearities, including hysteresis and synaptic plasticity.^207^ This nonlinearity arises from the unique characteristics of ions, distinguishing them from electrons. Owing to their ability to emulate gradual and history-dependent signal modulation, ionic memristors are often classified as analogue-type memristors that are particularly suited for replicating biological synaptic functions. To facilitate stable ion transport, gels have been widely employed as ionic conductors. Their hydrated, soft polymer networks provide mechanical compliance and support efficient ion migration while maintaining chemical and structural stability over time. This structure mimics the environment of synaptic cells in the human body, where ion conduction plays a crucial role in signal processing.^221^ Various gel materials have been explored for this purpose, including chitosan,^222^ cellulose,^223^ silk fibroin,^224^ collagen,^225^ gelatin,^226^ and ionogels.^227–229^

However, not all memristive systems that utilize gels and ionic conduction are classified as analog-type memristor. Early studies on gel-based ionic memristors primarily employed gels to achieve resistive switching rather than to exploit the unique dynamics of ion transport. In 2011, Koo *et Al*. combined polyelectrolyte hydrogel (poly­(acrylic acid) , pAA, and polyethylenimine, PEI) with liquid metal (an eutectic alloy of gallium and indium, EGaIn) to present a soft, bistable memristive device (Figure 9e).^217^ In this system, PEI gel creates a sufficiently high pH to remove the oxide regardless of potential; therefore, always maintain the electrode interface conductive. In contrast, the interface in contact with a lower pH PAA gel can be deposited or removed depending on the polarity of the electrode.^230^ A more detailed explanation about the electrical characteristics of polyelectrolyte gel will be discussed in Section 3.1. This work demonstrated the feasibility of hydrogels as high density, 3D, soft, and flexible ionic conductors with memristive characteristics. However, while the device showed high-low resistive switching via ionic conduction in polyelectrolyte-doped hydrogel, specific behaviors of synaptic plasticity were not analyzed.

In ionic systems, synaptic plasticity is typically governed by the mass and momentum of the ions during migration. Ren et al. reported an ionic memristor employing the same PAA/PEI polyelectrolyte bilayer combination with an indium tin oxide (ITO) electrode in 2022.^218^ Unlike the study forementioned, this system achieved memristive characters through the migration of polyelectrolyte chains and counterions under an external electric field (Figure 9f). The dynamic formation and vanishment of the ionic double layer at the interface of the polyelectrolyte bilayer enabled the emulation of various synaptic plasticity features such as PPF, STDP, STP, LTD, and LTP. Zhao group also reported a similar system in which PAA and PEI polyelectrolytes as dielectric layers doped with calcium ions in a memristor.^231^ This study demonstrated that the fixed charge type of the polyelectrolyte gel plays a crucial role in exhibiting resistive switching behavior and synaptic plasticity. These findings highlight the importance of material selection in tuning ionic interactions and memory dynamics in gel-based memristive systems.

Despite recent progress, maintaining stable depression and potentiation states for prolonged periods remains challenging for gel memristors as diffusion progressively erodes the concentration gradient over time. To address this, Zhang et al. demonstrated a three-layer bipolar ion-selective hydrogel structure capable of extending memory retention from seconds to hours. (Figure 9g).^219^ The device architecture consisted of sequential cation-selective, neutral, and anion-selective hydrogel layers. The central neutral layer played a critical role in modulating ion transport by acting as a tunable barrier, enabling the selective accumulation and depletion of ions under forward and reverse biases, respectively. To support their experimental findings, they also conducted numerical simulations to investigate the ion transport mechanism with focus on the effect of geometry and space charge density of gels by Nernst–Planck equations. Together, these results demonstrate that spatially engineered ion-selective architectures could offer a promising strategy for enhancing the temporal stability of ionic memory systems.

A structurally similar yet mechanistically distinct approach was demonstrated by Wang et al. in 2024. They reported ionic potential relaxation behavior using a trilayer hydrogel architecture composed of a polycationic hydrogel sandwiched between two neutral hydrogel layers with ITO/polyethylene terephthalate electrodes.^232^ The anion selective nature of the polycationic hydrogel induced localized concentration gradients of K^+^ and Cl^–^, enabling selective permeation of anions under an external field and hysteretic diffusion of cations after stimulation. Based on this phenomenon, they successfully mimicked short- and long-term plasticity of synapses such as PPD, PPF, LTD, and LTP. In addition to its functional versatility, the device showed remarkable flexibility, withstanding 180° bending and tensile stretchability of up to 100%. Meanwhile, Lei and Wu presented asymmetric trimeric hydrogel systems, in which a polyelectrolyte hydrogel was sandwiched between electroneutral high- and low-salinity hydrogel.^233^ In this study, the mobile ions within the polyelectrolyte hydrogel spontaneously accumulated near the low-salinity hydrogel, generating an internal electric field. This mechanism enables spatiotemporal control of ion flow, facilitating information recognition, processing, and memory formation, thereby supporting short-term plasticity and multimodal memory. These results emphasize the role of ion gradients and gel heterogeneity in encoding dynamic memory behavior.

In addition to the synaptic plasticity driven by simple ion migration, Han et al. demonstrated a two-terminal bipolar membrane (BM)-based ionic memristive system by integrating an ionic diode with reversible chemical precipitation and dissolution.^220^ They constructed a precipitation-based iontronic synapse such as LTP, LTD, STP, and STD, all of which were governed by the history of input stimuli. Under forward bias, the formation of precipitate serves as a physical blockage, inducing synaptic depression, whereas reverse bias potential facilitates the dissolution of precipitate, leading to synaptic potentiation (Figure 9h). Moreover, they emulated hippocampal neural circuits by integrating multiple independent systems for either excitatory or inhibitory configurations. This approach highlights the potential of chemically gated ionic systems for scalable neuromorphic architectures with reconfigurable signal processing.

Recently, the application scope of memristive systems has expanded beyond the synaptic plasticity stimulated by an electric signal, encompassing multimodal sensing and autonomous feedback control. In 2023, Tian et al. proposed an near-infrared optically responsive hydrogel consisting of Fe_3_O_4_ nanoparticles to emulate synaptic functions.^80^ This hydrogel functioned as an information processing unit, enabling the construction of an autonomous motion feedback system for the logical regulation of a robotic hand’s grasping behavior. In a similar vein, Luo et al. desingned a polypyrrole (PPY) nanoparticles-doped ionogel/pure ionogel heterojunction system for an artificial self-powered hemispherical retinomorphic eye in 2024.^234^ In this study, the photothermal effect and the thermoelectric conversion process induced by the migration of Li^+^ and TFSI^–^ ions from the pure-gel to the PPY-doped gel generated an inherent electric field within the heterojunction. They investigated neuromorphic photoperception, retinal transplantation, and visual restoration, allowing real-time dynamic visual imaging and motion tracking.

These gel-based ionic memristors differ fundamentally from their electronic counterparts by using mobile ions and ion-trapping mechanisms to encode the memory. This ionic mechanism enables analog signal processing and emulation of synaptic behaviors such as PPF, STDP, STP, LTD, or LTP, closely resembling the adaptive nature of biological synapses.^220^ Moreover, incorporating gels as the ionic conductors introduces mechanical compliance, transparency, and responsiveness to multiple external stimuli.^232,235^ These combined features position gel-based ionic memristors as promising candidates for low-power neuromorphic computing, adaptive sensors, and memory architectures in bioelectric systems.

## 3 Active Ionic Circuit Elements

### 3.1 Ionic Diode

#### 3.1.1 Polyelectrolyte Gels and Ionic Diodes

A diode is a two-terminal electric circuit element that exhibits rectifying behavior, allowing low resistance in one direction while maintaining a high resistance in the opposite direction. The most common type of electronic diode is the semiconductor p–n junction diode, formed by interfacing p- and n-type semiconductor materials. The p-type region contains an excess of positively charged carriers (holes), while the n-type region contains an excess of negatively charged carriers (electrons). Depending on the applied voltage across the p–n junction, the diode operates in one of two modes: forward bias where current readily flows and reverse bias where little or no current flows.

Analogous principles have been extended to ionic systems, where the rectification behavior is achieved through selective ion transport. Fundamental principles of ionic current rectification phenomenon were first demonstrated in nanofluidic and nanopore devices.^236,237^ These early demonstrations laid the foundation for subsequent developments in ionic diodes. A typical ionic diode consists of cation- and anion-selective membranes, analogous to p- and n-type semiconductors of electronic diode, respectively.^11^ One of the most common examples of an ionic diode is a structure composed of double layers of two oppositely charged polyelectrolyte gels.^238^ A polyelectrolyte gel is a polymer network that carries fixed charges of a single type, allowing only oppositely charged ions to remain mobile (Figure 10a). This property enables polyelectrolyte gels to function as ion-selective membranes, which regulate ion migration.

**Figure 10:**
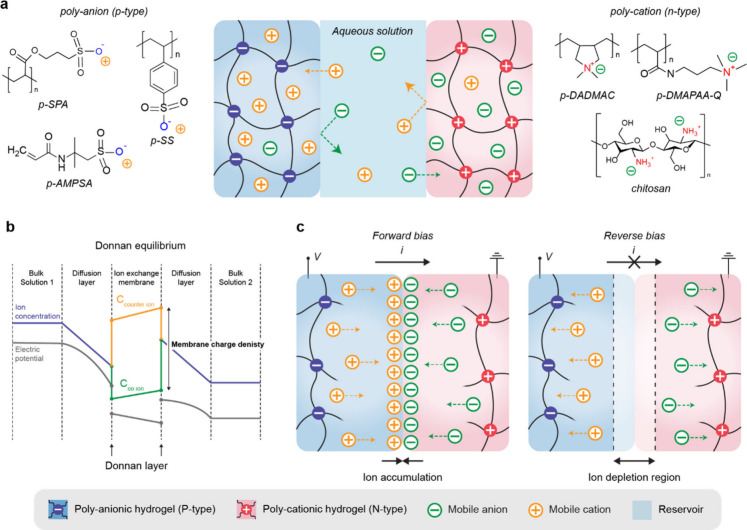
Polyelectrolyte gel and ionic diode. (a) Schematic depiction of p-type (left) and n-type (right) polyelectrolyte gel. Various chemical structures of p-type and n-type polyelectrolyte are presented. (b) Donnan equilibrium, an ion distribution, and electric potential at the membrane/solution interface. (c) Working mechanism of PN bipolar ionic diode.

Polyelectrolyte gels are classified into two groups based on their fixed charges. The p-type polyelectrolyte gel is a polymer network composed of negatively charged polymer backbones with mobile positive ions, while the n-type polyelectrolyte gel is composed of positively charged polymer backbones with mobile negative ions. The p-type polyelectrolyte gel, also known as polyanion gel, contains negatively charged functional groups such as sulfate (SO_3_^–^), carboxylate (COO^–^), or phosphate (PO_4_^2–^). On the other hand, the n-type polyelectrolyte gel, also known as polycation gel, contains positively charge functional groups such as ammonium (NH_3_^+^), pyridinium (C_5_H_5_N^+^), or imidazolium (C_3_H_5_N_2_^+^). Representative examples of polyelectrolyte gels are depicted in Figure 10a.

The fixed charges of polyelectrolyte gels and their mobile counterions generate electrostatic potential and asymmetric ion transport behavior, both of which play an important role in ionic diodes.^238^ The phenomenon is described by the Donnan equilibrium or Donnan layer model, which characterizes ion transport fluxes, *J*, and ion distribution at the gel/solution interface (Figure 10b).^239^ Within the gel, fixed charges carry a charge density, *X*. The counterions are the ions with a charge opposite to that of the fixed charges, while the co-ions, or commonly referred to as the free ions, are those with the same charge type as the fixed charges. Inside the gel, counterions accumulate to a higher concentration compared to that in the bulk solution outside the gel, whereas co-ions are present at a lower concentration. To maintain electroneutrality within the gel, the co- and counterion concentrations follow the relation:

6 $$C_{\mathrm{counterion}}=X+C_{\mathrm{co‐ion}}$$

Moreover, an EDL is formed at the gel/solution interface due to local charge separation. An excess charge on one side of the interface is balanced by an equal but opposite charge on the other side.^240^ This results in sharp gradients in ion concentration, as well as the electric potential, ϕ, across the EDL. Consequently, the co-ions are excluded from the gel, while counterions are accumulated within it, a phenomenon known as Donnan exclusion.^241^ This ion-selective behavior enables polyelectrolyte gels to mimic p- or n-type semiconductors.

Arranging p-type and n-type polyelectrolyte gel in sequence creates an asymmetry in ion transport across the interface of this p–n BM, or bipolar junction (BJ), which leads to electrically rectifying behaviors (Figure 10c).^242^ When a forward bias is applied to the BM, both the mobile cations from the p-type polyelectrolyte gel and the mobile anions from the n-type polyelectrolyte gel migrate toward the p–n junction. This ion accumulation effectively neutralizes the fixed charges of the gels at the interface, resulting in a locally elevated ion concentration. The increased ion concentration of the junction lowers the resistance, allowing appreciable current flow in the circuit. Conversely, under a reverse bias, ions are driven out of the junction, forming an ion depletion region at the junction. The resulting scarcity of mobile ions dramatically increases the resistance of the junction, thereby significantly restricting ion transport.^243^

Based on the ion-selective features of polyelectrolyte gel, various studies have investigated p-n bipolar ionic diodes. In the following section, we review gel-based ionic diodes with unique mechanical, chemical, and electrical properties. To provide a comprehensive understanding, we will cover fundamental principles as well as recent advances in materials, design, and functionality of gel-based ionic diodes.

#### 3.1.2 Ionic Diodes with Advantages of Gels

Cayre et al. were the first to propose the concept of polyelectrolyte diode.^244^ By sandwiching two aqueous polyelectrolyte gels with opposite fixed charges between Pt electrodes, these diode prototypes demonstrate a nonlinear current response (Figure 11a). In their design, poly­(styrene sulfonic acid) (pSSA) and poly­(diallyl dimethylammonium chloride) (pDADMAC) served as the p-type and n-type polyelectrolyte gels, respectively. To eliminate excess electrolyte ions, the gels were desalinated, ensuring that only polyelectrolyte counterions, hydroxide ions, and hydrogen ions contributed to charge transport across the BM. Notably, the ion rectification mechanism in this system is distinct from that of electrolytic ionic diodes, which achieve rectification through asymmetric Faradaic reactions at the electrodes. The use of polyelectrolyte gels in this system provided both mechanical stability and ease of fabrication. Following this foundational work, numerous studies have further advanced polyelectrolyte gel-based ionic diodes, expanding on their material properties and electrical characteristics.^245–247^

**Figure 11:**
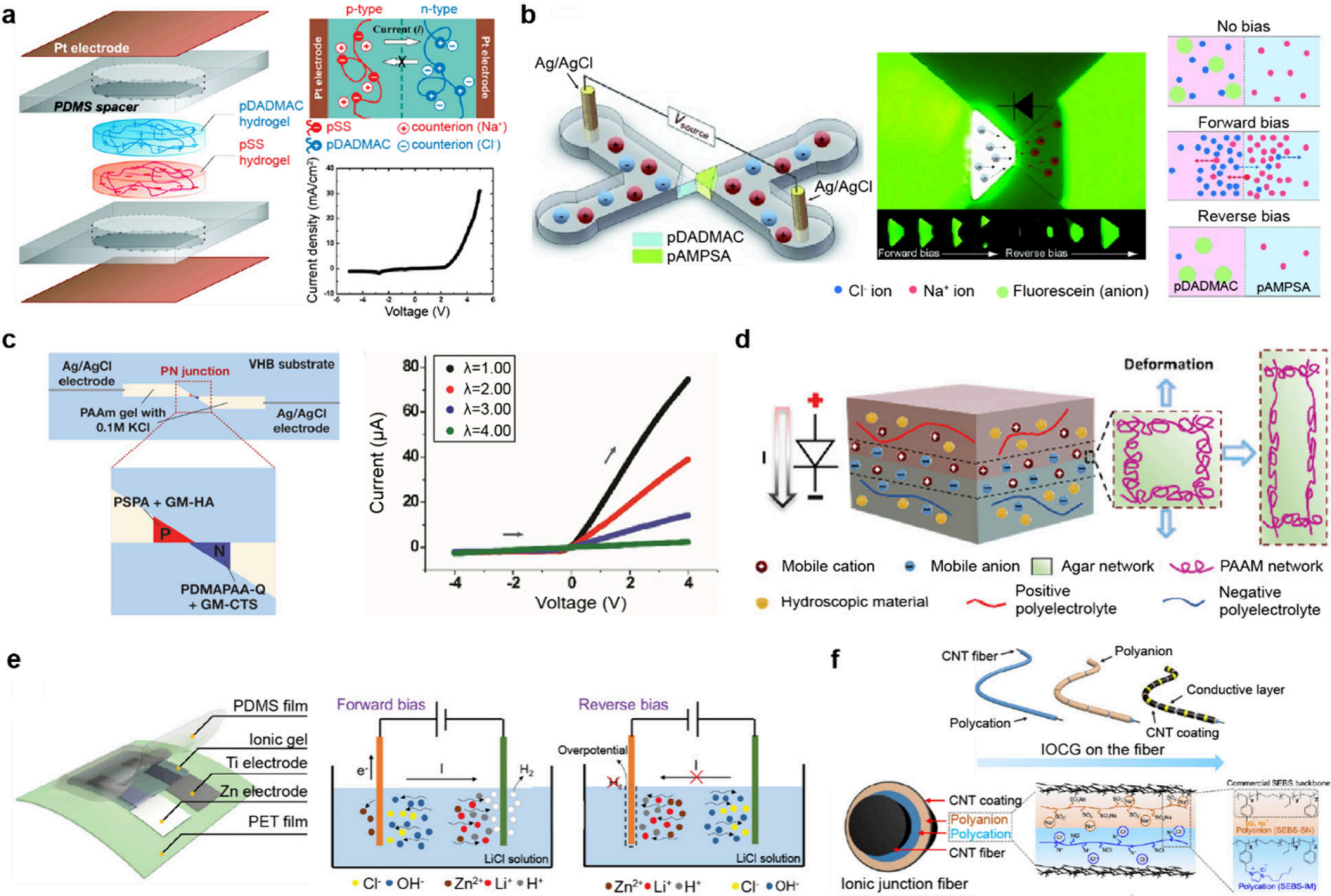
Various ionic diodes leveraging the advantages of gels. (a) Sandwich-type ionic diodes using two agarose gels with oppositely charged polyelectrolytes. Reproduced with permission from ref 244. Copyright 2007 American Chemical Society. (b) Microchip-type ionic diode composed of p- and n-type polyelectrolyte gels with fluorescein to visualize the ion accumulation and depletion. Reproduced with permission from ref 243. Copyright 2009 John Wiley and Sons. (c) Stretchable and transparent bipolar polyelectrolyte hydrogel-based ionic diode which can rectify ionic current under 300% strain. Reproduced with permission from ref 253. Copyright 2018 John Wiley and Sons. (d) Stretchable ionic skin composed of bipolar double-network polyelectrolyte hydrogels with hygroscopic substances. Reproduced with permission from ref 255. Copyright 2020 Royal Society of Chemistry. (e) Flexible and temperature tolerant ionic diode by using one ion-gel and an asymmetric reduction potential of aqueous H^+^. Reproduced with permission from ref 256. Copyright 2022 John Wiley and Sons. (f) Ionic-junction fiber made of bipolar polyelectrolyte gels and a carbon nanotube. It demonstrated functionality as ionic diodes and ionic bipolar junction transistors and exhibited synaptic characteristics. Reproduced with permission from ref 257. Copyright 2023 Springer Nature under CC BY 4.0 http://creativecommons.org/licenses/by/4.0/.

Building on the concept of polyelectrolyte gel-based ionic diodes, Han et al. advanced the field by developing ionic circuits that provide both fundamental insights and practical applications (Figure 11b).^243^ To ensure that the *I*–*V* behaviors were primarily governed by the impedance at the p–n junction, they minimized electrochemical reactions at the electrode surfaces by introducing Ag/AgCl electrodes. In addition, the polyelectrolyte gels were patterned into an hourglass shape to concentrate the electric field predominantly at the p–n junction. By incorporating an anionic fluorescent dye, fluorescein, they visualized and analyzed the dynamic distribution of the ion within the polyelectrolyte gels. Han et al. further analyzed the ion current rectification and hysteresis by investigating the effects of electric potential, scan rate, and electrolyte concentration.^248^ While gel-based ionic diodes have inherent limitations in switching speed and size, their intrinsic flexibility, stretchability, and biocompatibility position them as attractive candidates for future ionic information processors. In this context, there are various ionic circuit systems incorporating polyelectrolyte gel-based ionic diodes on microchips, further expanding the potential of ionic computing.^249–251^

Although polyelectrolyte gels are intrinsically flexible and stretchable, their mechanical properties and adhesion to substrates present challenges in integrating ionic diodes into wearable devices. To address this, Zhao et al. reported flexible ionic diode-based ion sensor.^252^ Moreover, Lee et al. developed stretchable ionic diodes using mechanically modified polyelectrolyte hydrogels that combined conventional charged monomers with methacrylated polysaccharides.^253^ They also introduced chemical adhesion between the gel and stretchable, transparent elastomeric substances, VHB (Figure 11c). The system maintained its rectifying properties even under strains exceeding 400% and withstood hundreds of stretching cycles up to 200%. In a related effort, Wang et al. reported the development of transparent and stretchable ionic diodes based on pAAm hydrogels, which were subsequently applied to construct ionic logic gates such as AND and OR configurations.^254^

Beyond their mechanical excellence, polyelectrolyte gel-based ionic diodes have also been explored for multifunctional biointerfaces. Ying et al. reported biocompatible, multimodal ionic skin capable of strain and humidity sensing as well as energy harvesting.^255^ The device withstood more than 400% strains without rupture due to its synergic effect of physically cross-linked agarose and covalently cross-linked pAAm networks within the polyelectrolyte gel (Figure 11d). It also converted both mechanical stimuli and humidity into four types of electrical signals: resistance, capacitance, open circuit voltage, and short circuit current. As the latter two signals are self-generated without an external power supply, the device demonstrated low-frequency energy harvesting from human walking. This approach shows the potential of the ionic diode for the development of next-generation wearable and implantable devices, particularly for applications requiring soft, multimodal human–machine interfaces.

Owing to its high water content, hydrogels are inherently susceptible to evaporation at temperatures exceeding biological levels and freezing at subzero temperatures. To address this challenge, Guo et al. demonstrated a temperature-resistant ionic diode enabled by an ethylene glycol/water binary solvent system in 2022 (Figure 11e).^256^ They exploited the asymmetric reduction potential of aqueous H^+^ at the surface of two different electrodes (Zn and Ti) to achieve a rectifying behavior. Furthermore, the water-locking effect of ethylene glycol molecules suppressed both the evaporation and freezing of free water molecules. They analyzed and optimized the electrical performances of the ionic diode by varying the concentration of ethylene glycol at −20 °C, 25 °C, and 60 °C. This study expanded the applicability of ionic diode by combining the inherent advantages of gels, such as transparency, flexibility, and ion selectivity, with extreme temperature tolerance.

Another advantage of gel-based systems is their processability. Moving beyond the two-dimensional (2D) planar or 3D bulk device structures, Xing et al. devised 1D ionic-junction fiber in 2023.^257^ They fabricated a kilometer-long ionic junction fiber by coating a CNT fiber core with sequential layers of polycations, polyanion, and CNT sheath (Figure 11f). Notably, this fiber was integrated in vivo as an artificial nerve pathway, interfacing with the gastrocnemius muscle and tibialis anterior muscle of mice. In the same year, Woo et al. reported a fiber-based flexible ionic diode composed of Zn-based anode and Ti-based cathode in a double-helical configuration, paired with a LiCl hydrogel electrolyte.^258^ Their device achieved a high rectification ratio of 2,773 and an output current of 28.2 mA under 3 V bias. With excellent geometric compatibility and stable electrical properties, the fiber-based ionic diode holds potential for clinical applications involving biological nerves such as nerve repair and rehabilitation.

Inspired by biological systems that respond dynamically to environmental stimuli, researchers have developed functional devices that can be directly modulated by external inputs. Bao et al. reported electro- and pH-modulated ionic rectification using hydrogel/conducting polymer heterogeneous membranes.^259^ Their system, composed of p­(AAm-*co*-AA) hydrogel and conducting polymer PPy, introduced asymmetries in chemical composition, structure, and surface charge polarity, leading to rectified ion transport. In a separate study, Ren et al. demonstrated an optically responsive ionic diode based on a photoresponsive hydrogel with light-modulated ion rectification.^260^ By exploiting photoresponsive changes in proton concentration within the hydrogel, the system exhibited enhanced ionic current rectification under UV irradiation. Remarkably, the system achieved a maximum rectification ratio of approximately 4 × 10^5^, demonstrating the potential of photomodulated iontronics for adaptive electronic systems.

#### 3.1.3 Ionic Diodes with Unique Characteristics of Ions

Beyond the ionic diodes that exploit the advantages of gels, another class of ionic diodes utilizing the unique properties of ions began to emerge in the 2010s. In this section, we highlight representative applications of ionic diodes which closely mimic biological systems or emphasize practical applications by leveraging the characteristic ion behaviors.

One of the key mismatches between polyelectrolyte gel-based ionic diodes and biological systems arises from a difference in ion distribution mechanisms. Unlike ionic diodes where ions are trapped or repelled by charged polymer networks, biological systems maintain a relatively uniform ion distribution across cellular membranes. Moreover, rather than relying on fixed charges in polymer backbones or surfaces, biological systems regulate ion transport through electrochemical gradients. Building on this distinction, Jiang et al. proposed gel polymer electrolytes that operate via an nonfaradaic ion-diffusion–migration mechanism.^261^ In this system, both cations and anions remain mobile and diffuse freely with rectification arising from differences in their diffusion/migration rates (Figure 12a). The limited diffusivity of Zn^2+^ and Cl^–^ in two disparate regions of the system led to the formation of an ionic double layer, which blocked further diffusion of mobile [EMIM]^+^ and CF_3_O_3_S^–^ ions across the junction. Expanding the concept of gel polymer electrolytes, Moon et al. demonstrated ionogel-based systems for flexible, low-voltage, and emissive displays fabricated on plastic substrates.^262^ By incorporation of electrochemically emissive luminophores into electrochemiluminescent ionogels, the system enabled quick and efficient light emission. This result further demonstrates the versatility of gel polymer electrolytes for multifunctional iontronic devices.

**Figure 12:**
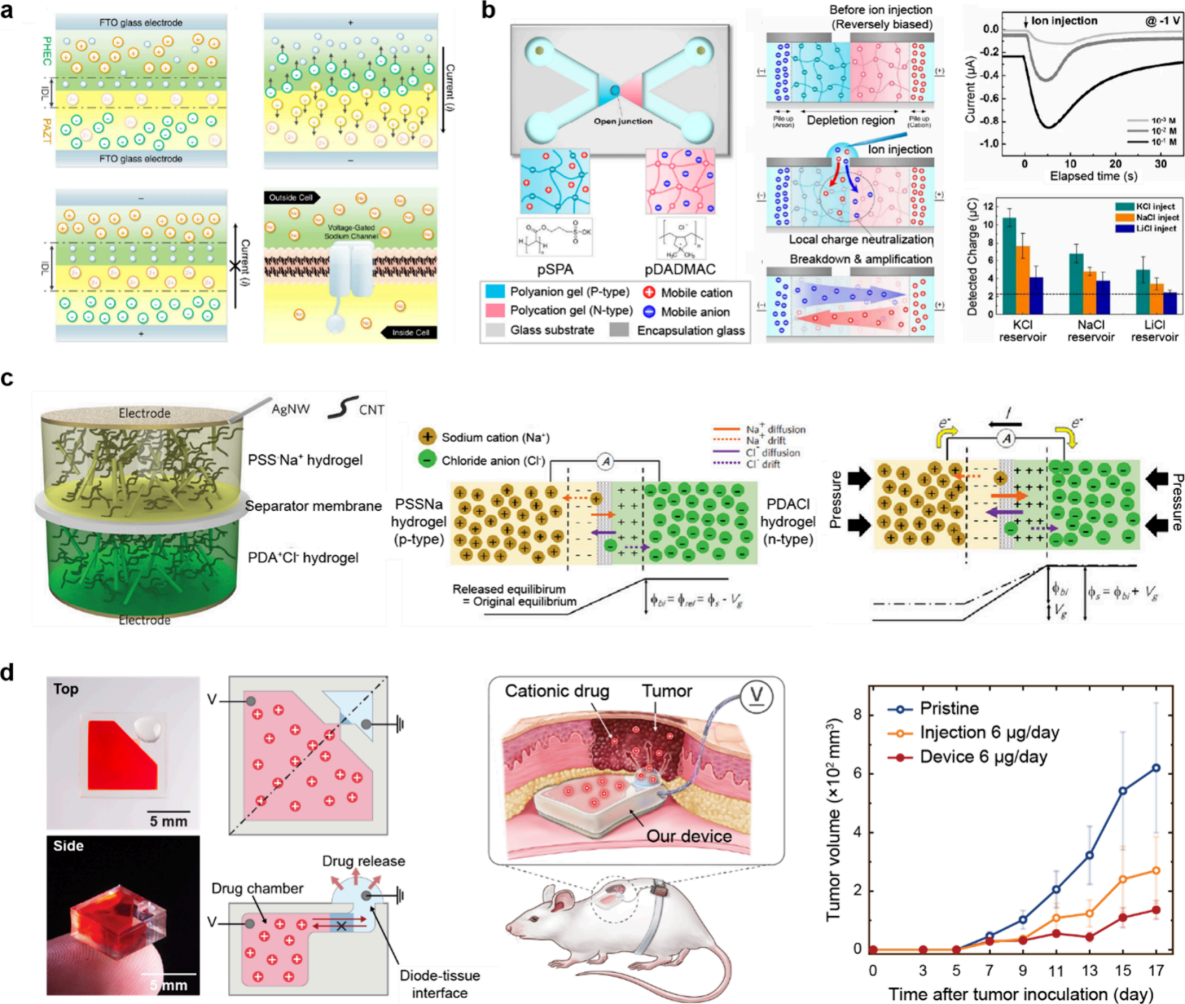
Applications of gel-based ionic diode using unique properties of ions. (A) Gel polymer electrolyte ionic diode. This system demonstrated higher temperature tolerance and thermal stability than hydrogel ionic diodes. Reproduced with permission from ref 261. Copyright 2022 Springer Nature under CC BY 4.0 http://creativecommons.org/licenses/by/4.0/. (B) Ion-to-ion signal sensing, conversion, and amplification system. The system facilitated direct communication between the ionic input signal and iontronic devices. Reproduced with permission from ref 263. Copyright 2019 The National Academy of Sciences. (C) Hydrogel ionic diode-based mechanical energy harvesting device. Reproduced with permission from ref 266. Copyright 2021 John Wiley and Sons. (D) Polyelectrolyte hydrogel-based ionic drug delivery system. The efficiency and off-target immune toxicity was analyzed through an in vivo antitumor drug delivery experiment. Reproduced with permission from ref 268. Copyright 2024 John Wiley and Sons.

Besides, biological systems primarily rely on the transport of ions such as K^+^, Ca^2+^, Cl^–^, and neurotransmitters to transmit signals, whereas conventional ionic diodes operate under an external electric bias. To bridge this gap, Lim et al. proposed an open-junction ionic diode structure for the direct transmission and amplification of ionic signals in 2019 (Figure 12b).^263^ They verified the ion-to-ion signal amplification mechanism through the ion-selective response of fluorescent dyes and finite element method simulations. The signal amplification behavior of the system was further examined under various reservoir concentrations, reverse bias voltages, and ions species. Notably, the amplification ratio differed depending on the ionic species, even when their mobilities within the polyelectrolyte gel were similar. This finding suggests the potential for developing an ion-to-ion signal transmission system capable of distinguishing specific ion species, paving the way for selective ionic signal processing. Building on this concept, Yoo et al. reported a noninvasive in vitro ion monitoring system using the selective ion-to-ion current amplification phenomenon for real-time chemical sensing.^264^

As gel-based ionic diode systems are still in their early stages, most applications remain at the prototype level. Nevertheless, we present several studies demonstrating practical applications.

In 2017, Zhou et al. developed a biocompatible and flexible energy harvesting device that utilizes a hydrogel-based ionic diode as an electromechanical transducer.^265^ Their system exhibited superior performance in harvesting low frequency mechanical energy, outperforming conventional mechanical energy harvesters, which typically operate efficiently only at high frequencies. In 2021, Zhang et al. reported an ionic diode systems designed for harvesting ultralow-frequency mechanical energy.^266^ They embedded CNTs and silver nanowires within a layered structure composed of two hydrogel polyelectrolytes, polystyrenesulfonate (pSS) and pDADMAC (Figure 12c). Upon mechanical stimulation, ion diffusion occurs and disrupts the original equilibrium of mobile Na^+^ and Cl^–^ ions. As a result, ion transport within the device induces current flow through an external circuit. Their mechanical energy harvesting performance exceeded that of existing technologies at 0.01 Hz by several orders of magnitude. This frequency range is particularly significant given that typical human motions occur below 1–5 Hz, whereas most current harvesters operate efficiently only at frequencies above 10 Hz. More recently, in 2025, Gao et al. demonstrated a hybrid electromagnetic and moisture energy harvesting system.^267^ By using moisture-induced ionic rectification as a bridge mechanism, they established a coupling effect between environmental humidity and electromagnetic energy harvesting. These systems broadened the operational versatility of ionic rectification systems.

Yoo et al. reported an ionic diode-based drug delivery system capable of spatiotemporally controlled yet sustained drug release in 2024.^268^ Unlike conventional p-n bipolar junction ionic diodes, their system attained ion rectification behavior by introducing a single n-type polyelectrolyte gel combined with geometric effects and ion concentration gradients (Figure 12d). This design enabled precise drug migration at the nanogram to microgram scale and ensured continuous diffusion to the lesion site without generating hydrodynamic pressure, a common limitation of conventional active drug delivery systems. Additionally, when loaded with the antitumor drug doxorubicin, the system exhibited superior immune cell viability and tumor suppression compared to intratumoral injections over a 17-days of in vivo experiments in freely moving tumor-bearing mouse models. Owing to its reliance on unidirectional ion migration, it shows potential as a universal drug delivery platform. Similarly, Oh et al. proposed a chemical delivery probe based on the inverted ion current rectification phenomenon, using a polyelectrolyte gel-filled micropipette.^269^ Their system enabled localized in vitro delivery of glutamate to hippocampal neurons, demonstrating the utility of the ion rectification phenomenon for neuronal modulation.

Gel-based ionic diodes rely on asymmetric ion transport across charge-selective gel interfaces to achieve rectification. The ionic conduction in gel enables operation in aqueous or biocompatible environments without the need of rigid packaging.^11^ Soft and stretchable configuration of gel also allows seamless integration into biological systems.^270^ Moreover, the use of polyelectrolyte gels offers tunability in terms of ion selectivity, response time, and threshold behavior. These advantages propose gel-based ionic diodes as promising components in ionic logic systems, sensors, bioelectric circuits, and implantable ion delivery systems.^271^

### 3.2 Ionic Transistor

The transistor, a three-terminal system that amplifies or switches electric signals, is one of the greatest inventions of the past century.^272^ Massive numbers of transistors are integrated into small chips to enable rapid processing and complex calculations, which form the foundation of the information age.^273^ Typical electronic transistors are broadly categorized into two groups by their working mechanism: field effect transistors and bipolar junction transistor. Despite variations in their structures and operating principles, all electronic transistors share a fundamental characteristic: the electron is their primary charge carrier.

Ionic transistors, which utilize ions as the primary charge carriers, have also gained increasing attention over the past few decades. Unlike electronic transistors, ionic transistors are broadly classified into three groups based on their working mechanism: organic field effect transistors (OFETs), organic electrochemical transistors (OECTs), and ionic bipolar junction transistors (IBJTs). In the following section, we will first examine the structure, mechanism, and electrical properties of each type with a particular focus on the role of gels in their operation. Then, we introduce some representative studies that could demonstrate their potential applications.

#### 3.2.1 Gel-Based Organic Field Effect Transistors (OFETs)

OFETs consist of five essential components: the source, the drain, the gate, the organic semiconductor layer, and the dielectric.^274^ OFETs are classified by the type of their organic semiconductor: p-type, which relies on holes, and n-type, which relies on electrons. The representative top-gate-top-contact structures of p-type OFETs (Figure 13a) and n-type OFETs (Figure 13b) are illustrated. The source and the drain form the main conducting channel of the electric circuit, while the gate controls the charge induced into the channel. Hence, charge carriers move from the source to the drain (*I*_*D*_), with the electric potential applied to the gate (*V*_*G*_) regulating this movement. The organic semiconductor layer connects the source and drain, playing the role of charge generation, injection, and transport. During OFET operation, an external gate voltage induces electrostatic charge accumulation at the gate/dielectric interface, leading to the formation of an oppositely charged layer at the semiconductor/dielectric boundary.^275^ These accumulated charges within the semiconductor form a conductive channel with a thickness of only a few nanometers, allowing carriers to migrate from the source to the drain.^163^

**Figure 13:**
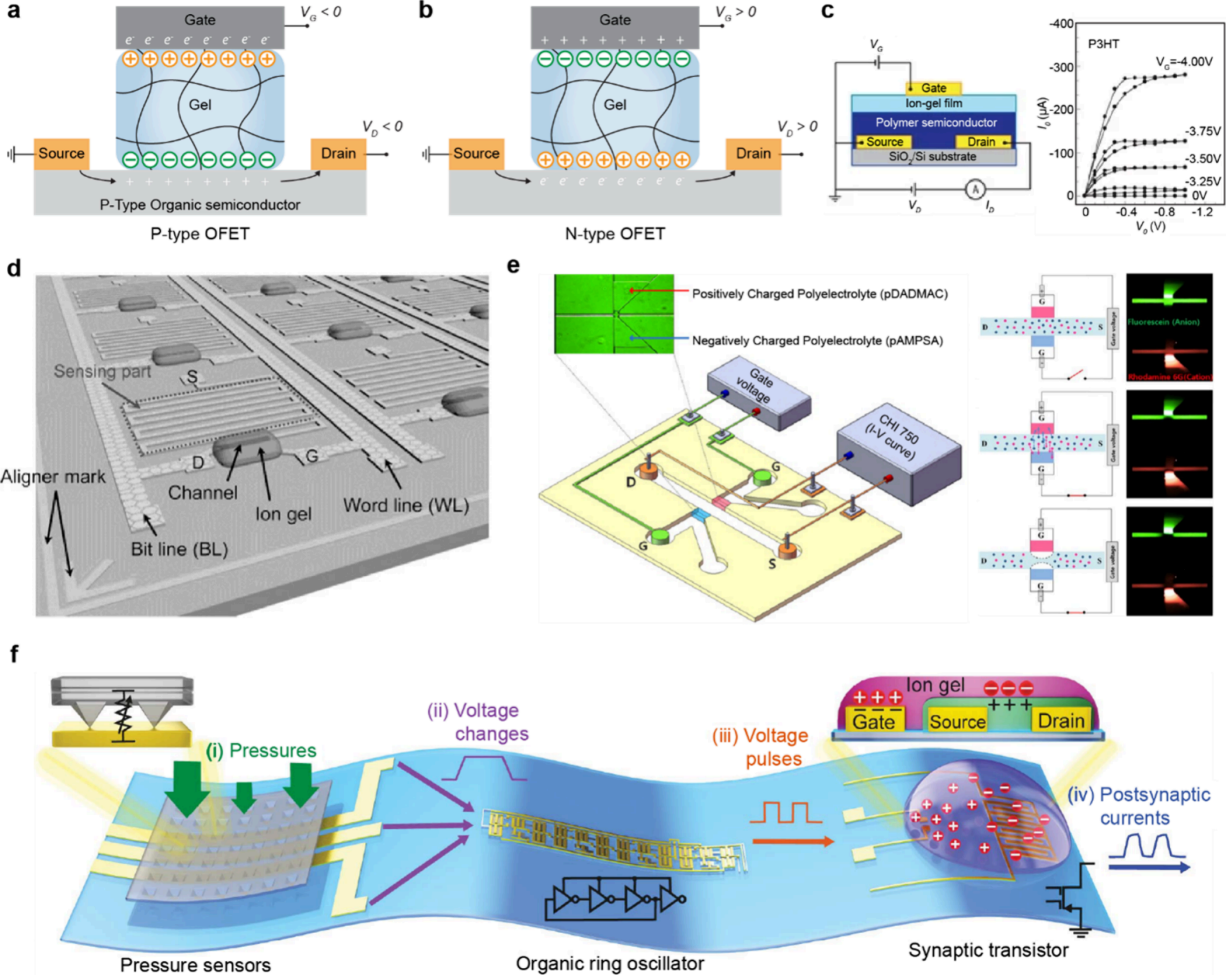
Mechanisms and applications of organic field-effect transistor (OFET). (a,b) Schematic depiction of p-type OFET (a) and n-type OFET (b). (c) Printable ion-gel film used as dielectrics for flexible electronics. The high polarizability of ion-gels enables simplified transistor architecture. Reproduced with permission from ref 277. Copyright 2008 Springer Nature. (d) Transparent, low-power pressure sensor by graphene field-effect transistor with an ion-gel gate dielectric. Reproduced with permission from ref 278. Copyright 2014 John Wiley and Sons. (e) Polyelectrolyte junction field-effect transistors which control ionic flow in an aqueous medium. Reproduced with permission from ref 279. Copyright 2010 AIP Publishing. (f) Artificial afferent nerve based on a synaptic field-effect transistor. Ionogel was used as the gate dielectric, and allowed the active channel to be gated by multiple electrodes. Reproduced with permission from ref 280. Copyright 2018 American Association for the Advancement of Science.

As discussed in Section 2.2, gels have been explored as promising materials for dielectric due to their high dielectric constant (*ε*), mechanical compliance, biocompatibility, and self-healing properties.^276^ These attributes make gels highly attractive for OFET, particularly in stretchable, wearable, and biointegrated devices. In the following sections, we will introduce various OFET studies with gels and analyze their mechanical properties, electrical performance, and potential applications.

Although there is various research using hydrogel-based OFETs,^281–283^ ionogels are widely used in the field of OFETs due to their nonvolatility. In 2008, Cho et al. first introduced ionogel gated thin-film transistor, which share the same architecture as OFETs, for flexible, printable electronics with high electrical performance.^277^ They used poly­(styrene-*block*-ethylene oxide-*block*-styrene) (PS-PEO-PS) mixed with an IL, [EMIM]^+^[TFSI]^−^, for the dielectric ionogel (Figure 13c). The ionogel exhibits high capacitance (∼0.02 μF/cm^2^) even at a high frequency of 10 kHz, surpassing that of conventional ceramic or polymeric gate dielectrics due to its large concentration and high mobility of ionic species within the gel. They also printed all components of the system onto a plastic substrate by using a commercial aerosol jet printing technique, demonstrating the potential processability of gel-based OFET prototypes. Since then, numerous studies have been conducted on the ionogel gate dielectric for OFET.^276,284–289^

Among these studies, Cho and his team developed a low voltage, transparent pressure sensor matrix (4 × 4 pixel) for e-skin application in 2014.^278^ They integrated coplanar graphene with an ionogel gate dielectric for OFETs on a flexible plastic substrate (Figure 13d). The device exhibited outstanding pressure sensor properties, including a high transparency (∼80%) across the visible range, a low operating voltage of less than 2 V, a high pressure sensitivity of 0.12 kPa^–1^, and an excellent mechanical durability over 2,500 cycles. This study suggested the potential of gel-based OFETs for applications in transparent, flexible, and stretchable electronics. Similarly, Sun et al. introduced an electronic skin strain sensor in 2015, based on coplanar graphene and an ionogel gate dielectric.^290^ They developed transparent and stretchable sensors on a PDMS substrate, enabling real-time monitoring of human hand movements. Recent studies have continued to explore ionogel dielectrics and graphene-based structures for OFETs, further advancing their applications in flexible and transparent electronics.^291,292^

Unlike conventional OFET structures, Kim et al. suggested a polyelectrolyte junction field effect transistor that operates in an aqueous medium.^279^ The system consists of p- and n-type polyelectrolyte gels on the gate channel, which regulate the ion migration within the main channel (from the source to the drain) based on the extent of ion depletion induced by gate voltage (Figure 13e). While the electrical behavior of the system appears similar to that of conventional OFET, it differs significantly in terms of the charge carriers, the working principle, and the operating medium. As this microfluidic system eliminates the need for complex fabrication processes, it holds potential for broader applications in aqueous information processing.

The potential of gel-based OFETs for human–machine interface, where flexibility, biocompatibility, and electric conductivity are highly required, has been frequently discussed. Kim et al. reported an artificial afferent nerve system based on ionogel dielectric OFET.^280^ The system consists of three main components: pressure sensors, an organic ring oscillator, and a synaptic transistor (Figure 13f). The ionogel dielectric in the synaptic transistor not only facilitated low-voltage operation but also enabled post-synaptic current behavior. The accumulation and gradual decay of charges within the ionogel allowed for continuous adaptation to sensory input, an essential feature for neuromorphic computing. Furthermore, analogous to how dendrites of post-synaptic neurons integrate action potentials from multiple presynaptic neurons, a single synaptic transistor could sum voltage inputs from multiple ring oscillators. To demonstrate a hybrid bioelectronic reflex arc, their system was connected to the biological efferent nerves of a discoid cockroach, successfully triggering muscular actuation in its leg.

#### 3.2.2 Gel-Based Organic Electrochemical Transistors (OECTs)

OECTs were first developed in the 1980s and have gained widespread attention for their applications in sensitive and flexible bioelectronics.^293,294^ Their use in neural recording elements,^295^ ion sensors,^296^ gas sensors,^297^ biomolecule detectors,^298,299^ artificial synapse,^300–302^ and cell activity monitors,^303^ is driven by their mechanical resilience, low operating voltages, high transconductance, and excellent biocompatibility.^304,305^ While the structure of the OECTs is similar to that of the OFETs, their operation mechanisms and principles differ substantially. The most significant difference in their structures is the use of an electrolyte, which replaces the gate dielectric layer of OFETs and enables direct ion transport to the channel. During operation, a voltage bias (*V*_*D*_) is applied between the source and the drain electrode, driving an electric current (*I*_*D*_) through the organic channel. The magnitude of the *I*_*D*_ is regulated by an input voltage at the gate electrode (*V*_*G*_), as ions injected into or extracted from the electrolyte to channel modify the doping level of the channel material.^306^

OECTs are classified according to the type of channel materials, such as organic semiconductors or conductive polymers, which can transport either holes (p-type) or electrons (n-type). Moreover, the operational mode of an OECT, accumulation or depletion, is determined by the interaction between the channel and the electrolyte as well as the polarity of the applied gate bias. In accumulation mode, gate bias causes ion injection into the channel, increasing its conductivity and turning the device ON. In depletion mode, ions are extracted from the channel upon gate bias, reducing conductivity and switching the device OFF.^294^ The representative designs of accumulation mode OECT (Figure 14a) and depletion mode OECT (Figure 14b), along with their *I*–*V* curves (Figure 14c) are depicted. Hydrogels and ionogels have been introduced as solid-state electrolytes due to their flexibility, mechanical compatibility with soft tissue (Young’s modulus ranging from 500 Pa to 500 kPa), high ion conductivity, and biocompatibility.^303,307^

**Figure 14:**
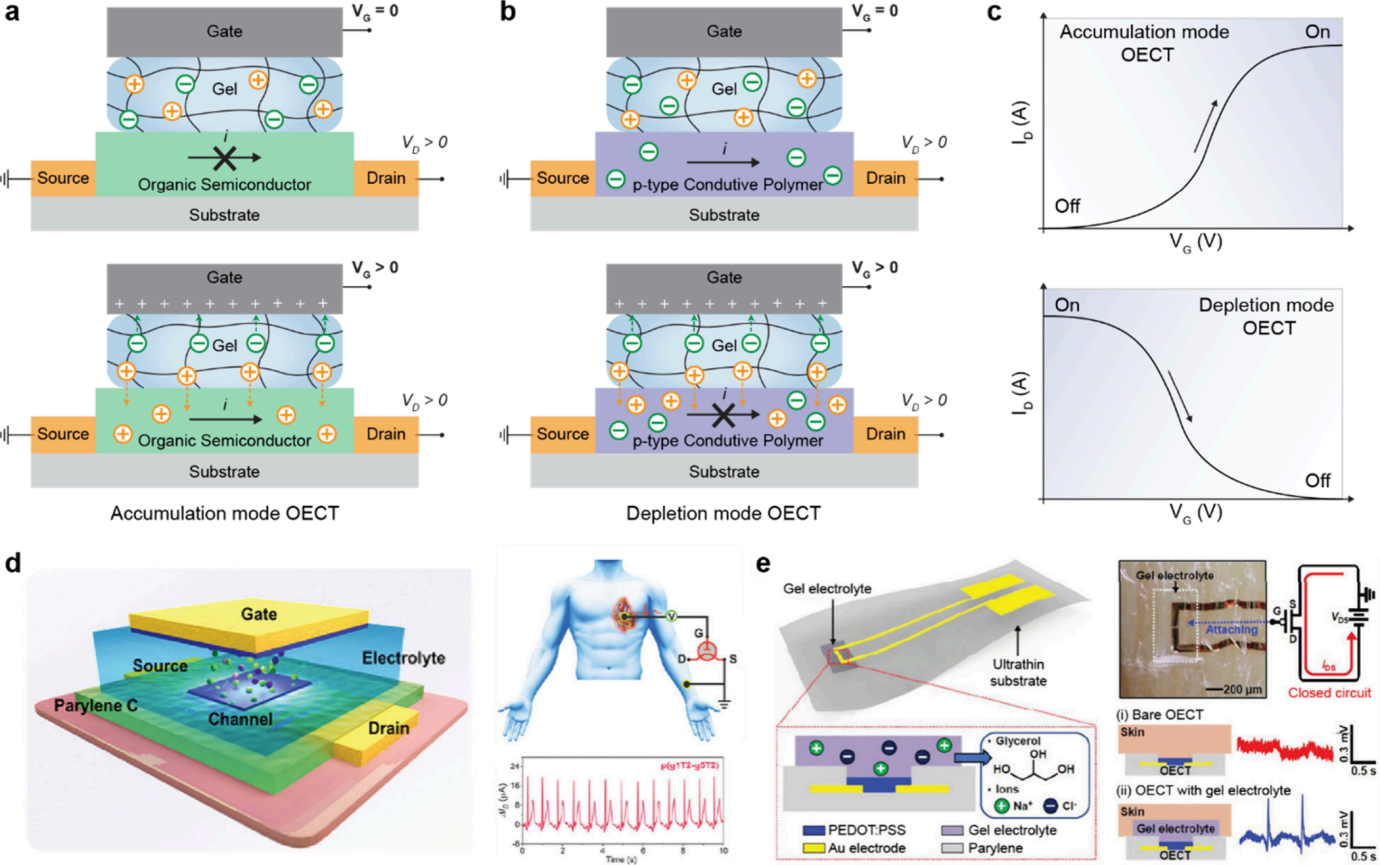
Mechanisms and applications of organic electrochemical transistor (OECT). (a,b) Schematic depiction of accumulation mode OECT (a) and depletion mode OECT (b). (c) *I*–*V* curves of the accumulation mode of the OECT (top) and depletion mode of the OECT (bottom). (d) Solid state organic electrochemical transistors and its application as electrocardiography monitoring system. Ionic gels were used for flexibility and ion migration efficiency. Reproduced with permission from ref 308. Copyright 2022 John Wiley and Sons. (e) Nonvolatile glycerol ionic gel was used for nonvolatile and thin electrolyte reservoir in OECT. Reproduced with permission from ref 309. Copyright 2019 John Wiley and Sons.

One of the most proficient target applications of the OECTs is a sensor to monitor electrophysiological signals. For example, OECTs with gel electrolyte containing IL have been used in wearable, noninvasive sensors capable of collecting biometric signals from dry skin surfaces,^310^ Lee et al. presented an electrophysiological sensor with an ultrathin 6 μm layer of a nonvolatile gel electrolyte in 2019 (Figure 14d).^308^ This system showed good mechanical stability and conformal contact with the skin, which is essential for wearable devices. It also demonstrated stable electrical performance over 1 week with a signal-to-noise ratio of 24 dB in their on-skin electrocardiogram (ECG) monitoring experiment. Building on these results, recent work has further expanded the integration of OECTs into wearable ECG platforms, highlighting their practicality for long-term, on-skin health monitoring technologies.^311,312^

However, to meet the thermal requirements of commercially packaged electronic devices, OECTs are required to operate above 90 °C. In 2023, an ionogel electrolyte-based OECT with a wide operation temperature range from −50 to 110 °C was developed by Wu et al. (Figure 14e).^309^ They introduced poly­(vinylidene fluoride-*co*-hexafluoropropylene) (PVDF-*co*-HFP) copolymer mixed with glycerol and IL of [EMIM]^+^[BF_4_]^−^. This nonvolatile ionogel exhibited outstanding mechanical, electrochemical, and thermal stability and was also able to operate at kHz frequencies.^313^ Furthermore, the system was successfully applied to continuous ECG monitoring devices, demonstrating reliable performance under extreme conditions from −50 to 110 °C. In addition, glycerol-based gels have been further investigated to enhance elasticity, electrical stability, transconductance, and durability.^314–316^

#### 3.2.3 Gel-Based Ionic Bipolar Junction Transistors (IBJTs)

IBJTs have a distinct structure and operating mechanism compared with OFETs and OECTs. While all three types of transistors share a three-terminal structure, the names of the terminals differ: collector (C), emitter (E), and base (B). Representative illustrations of pnp-type IBJT in a cutoff mode (Figure 15a) and active mode (Figure 15b) are depicted, where the emitter and the collector are cation-selective (p-type) while the base is anion-selective (n-type). As indicated by their nomenclature, IBJTs consist of two pn junctions sharing a base region.

**Figure 15:**
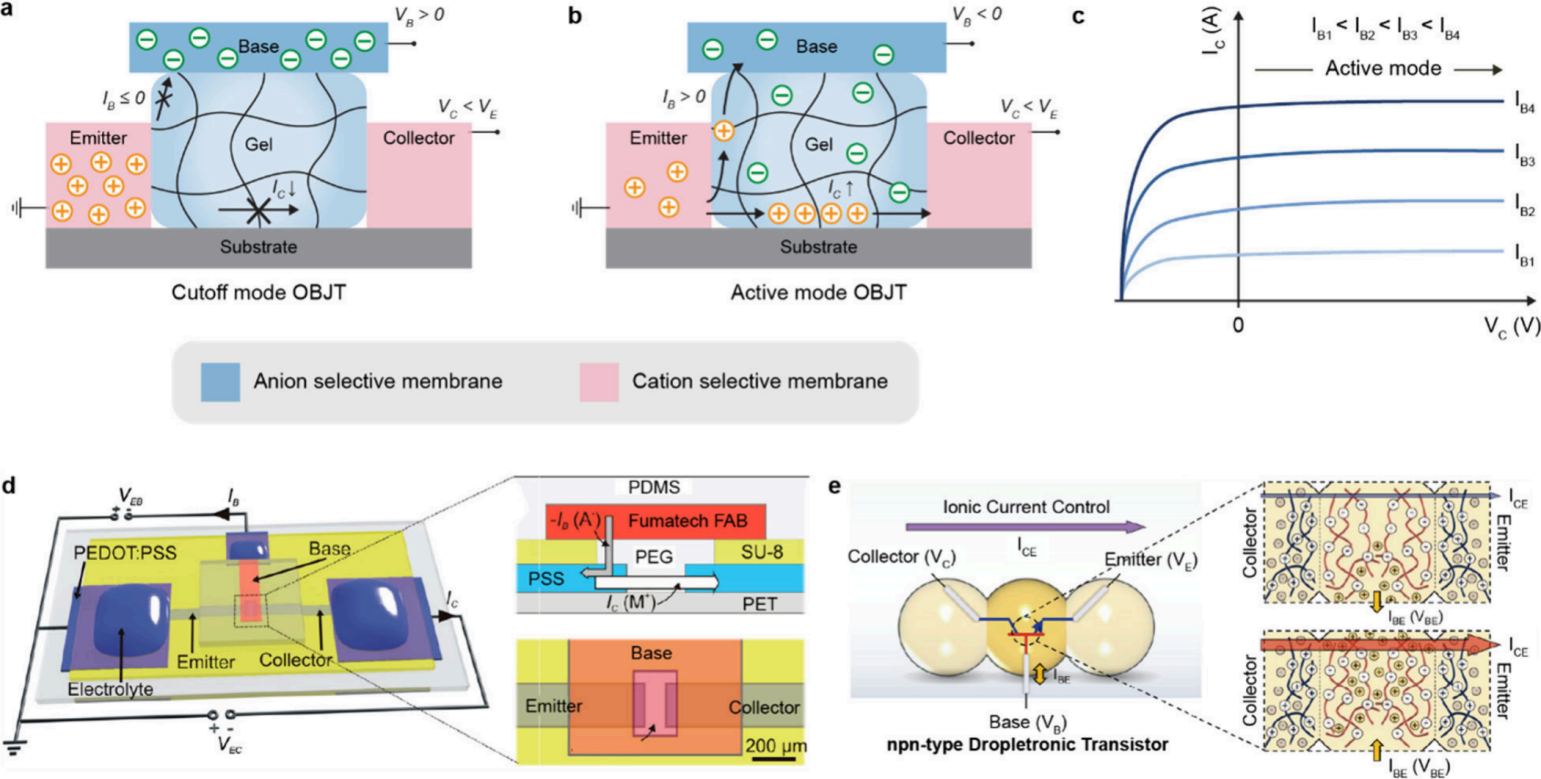
Mechanisms and applications of ion bipolar junction transistor (IBJT). (a,b) Schematic depiction of cutoff mode of the OECT (a) and active mode of the OECT (b). (c) Characteristic *I*_*C*_–*V*_*C*_ curves under various *I*_*B*_. (d) Architecture of the prototype IBJT. The mobile ions are extracted from or accumulated within the intermediate gel layer. Reproduced with permission from ref 318. Copyright 2010 The National Academy of Sciences. (e) Microscale droplet silk hydrogel assembly for an npn-type dropletronic transistor. Reproduced with permission from ref 317. Copyright 2024 The American Association for the Advancement of Science.

The operating mechanism of IBJTs mainly relies on Donnan exclusion in ion selective regions. In the cutoff mode of a pnp-type IBJT, when the base-emitter current (*I*_*B*_) ≤ 0, ion depletion is enhanced at the C/B and B/E interface, resulting in a small collector-emitter current (*I*_*C*_). In contrast, in the active mode, a positive **I*_B_* facilitates cation transports between the collector and the emitter, enabling transistor operation with an increased *I*_*C*_. Therefore, unlike the case for OFETs and OECTs, where *I_D_* is controlled by *V*_*G*_, *I*_*C*_ is regulated by *I*_*B*_ in IBJTs. The representative *I*_*C*_–*V*_*C*_ curves of IBJT are depicted in Figure 15c. The gel can function either as an electrolyte placed between the three ion-selective terminals or as the ion-selective terminal itself. By switching the position of cation- and anion-selective regions, an npn-type IBJT can be constructed, exhibiting transistor characteristics based on anion transport.^317^

The concept of gel-based IBJT was first proposed by Tybrandt in 2010 (Figure 15d).^318^ Their system used overoxidized poly­(3,4-ethylenedioxythiophene)-polystyrenesulfonate (PEDOT:PSS) as the cation-selective emitter and collector region, while anion-selective Fumatech FAB was applied to the base region. A PEG gel was placed between the three terminals as an electrically neutral intermediate layer, effectively separating them. Under reverse and forward bias voltages, mobile ions are extracted from or accumulated within the PEG layer, respectively, contributing to the maintenance of a high rectification ratio.^319^ This study demonstrated that the IBJT shares structural and functional similarities with electronic bipolar junction transistors.

Various studies have attempted to develop IBJT with practical structures and applications. Xing et al. developed an ionic junction fiber designed for ionic diodes and IBJTs.^257^ They demonstrated synaptic functionalities by using fiber memory capacitance and applied their system to an in vivo artificial nerve pathway. In 2024, Zhang et al. reported microscale, modular, soft, biocompatible, and self-assembled dropletronic devices for IBJT.^317^ Using a protein modification technique, they developed ion selective silk hydrogel droplets that can be sequentially positioned in a surfactant-containing oil and activated by UV cross-linking to form continuous structures (Figure 15e). They also fabricated diodes, reconfigurable logic gates, electrophysiological recording systems, and artificial synapses. This study presents the potential of IBJT as a biocompatible approach for the direct communication of multiple vital ions. Similarly, Huo et al. also reported 3D printable modular ionic microgels for ionic diodes, ionic rectifiers, ionic touchpads, and IBJT.^320^

Gel-based ionic transistors leverage key material advantages such as softness, ionic conductivity, and processability of gels to enable diverse signal transduction mechanisms that are challenging to realize with conventional solid-state electronics. Depending on their type of gating mechanism and how the channel is modulated, these devices are categorized into OFETs, OECTs, and IBJTs. Each device offers distinct advantages tailored to specific functional applications. (Table 1) OFETs employ electrostatic and capacitive gating via a dielectric, enabling fast switching and low voltage operation. These properties make them well-suited for flexible logic circuits and printed electronics.^292^ OECTs rely on faradaic ion-electron coupling to achieve low power consumption, high transconductance, and sensitive signal amplification. As a result, they are particularly beneficial for electrophysiological sensors, wearable bioelectronics, and neuromorphic systems.^303^ IBJTs further expand the design landscape of ionic transistors by allowing direct ionic amplification through base current modulation. This feature provides unique advantages for ion-selective applications. Compared with rigid semiconductor transistors, gel-based ionic transistors offer superior aqueous stability, mechanical compliance, and biocompatibility. These characteristics support their integration into stretchable or biointerfaced platforms.

**Table 1:** Key Characteristics and Applications of Gel-Based Ionic Transistors

Type	Driving mechanism	Switching speed	Advantage in gel-based system	Representative applications
**OFET**	Capacitive coupling	Fast (∼ms)	Fast switching, low-voltage operation	Flexible circuit, printed electronics
**OECT**	Faradaic reaction	Moderate (∼s)	High transconductance, high signal amplification	Biosensing, neuromorphic computing
**IBJT**	Ion current amplification	Slow (∼s or longer)	Ion-to-ion amplification, aqueous compatibility	Ion-selective system, drug delivery

## 4 Ionic Power Sources

Various types of power sources utilizing ions have emerged, making use of ion transport and ionic interactions, rather than conventional electron flow. These gel-based ionic power sources operate through mechanisms such as ion diffusion, charge separation, and potential gradients, enabling energy harvesting from mechanical motion, thermal gradients, concentration differences, and environmental moisture. In this section, we explore five key types of ionic power sources that rely on triboelectric, thermoelectric, concentration-driven, piezoionic, and moisture-based energy harvesting mechanisms. It is important to note that this Review excludes electrochemical battery systems based on redox reactions, such as lithium-ion batteries and fuel cells.

### 4.1 Gel-Based Triboelectric Nanogenerators (TENGs)

A TENG generates electricity through contact electrification and electrostatic induction. Initially, the system is at equilibrium with no net charge separation (Figure 16a). When a contact material encounters the elastomer, electron transfer occurs due to differences in triboelectric properties, leaving the contact material positively charged and the elastomer negatively charged (Figure 16b). As the contact material moves away, the transferred charges remaining on the surface attract cations within the gel, while repelling anions (Figure 16c). This charge imbalance induces an electron flow through an external circuit, generating electrical output. When the contact material moves back, the process reverses due to electrostatic induction, leading to AC generation (Figure 16d). Building on this gel-based ionic energy harvesting mechanism, researchers have developed ionic communicators,^78,321^ battery-free wearable sensors,^322–325^ and electrotherapeutic devices.^124,326–328^

**Figure 16:**
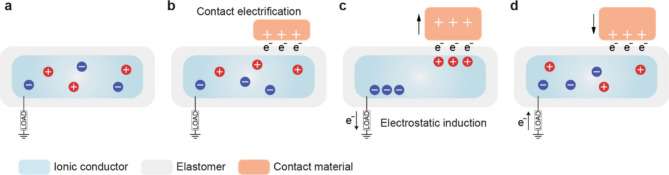
Potential generation mechanism of a TENG with soft materials. (a) The ionic conductor maintains charge balance in its initial state. (b) When two materials (contact material and elastomer) with different triboelectric properties come into contact and then separate, electrons transfer between them. (c) When the contact material moves away, the transferred electrons at the contact surface attract cations in the ionic conductor. The repelled anions generate current in the load by pushing electrons through the wire. (d) As the contact material approaches again, a reverse electron flow is induced by electrostatic induction.

Lee et al. developed the first gel-based ionic TENG as a transparent, self-cleaning, and attachable power source for wearable electronics and human–machine interfaces (Figure 17a).^321^ Unlike conventional TENGs, which often struggle with transparency and flexibility, this approach integrates hydrogel electrodes with chemically anchored elastomers, ensuring mechanical stability and high optical transmittance. The fluorinated surface treatment enhances triboelectric charge transfer and provides anticontamination properties, thereby improving electrical output and long-term stability. Additionally, the gel-based structure enables stretchability and adaptability to various surfaces, making it highly suitable for real-time, on-skin applications. A notable application includes the development of an ionic communicator, where gel-based TENGs translate human touch into wireless digital signals, enabling interactive and wearable communication interfaces.

**Figure 17:**
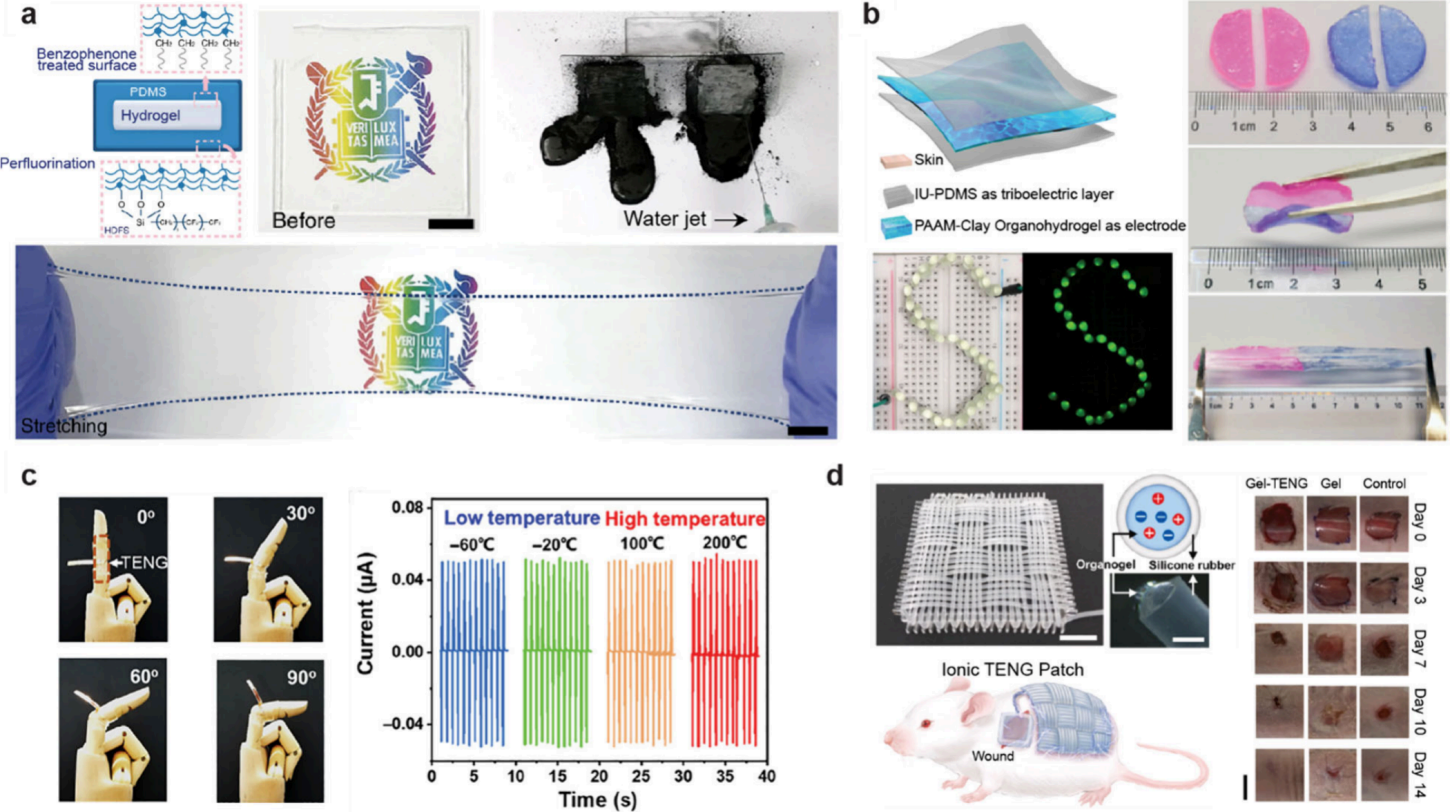
TENGs using an ionic conductor. (a) Highly stretchable (∼330%), self-cleanable, and transparent (∼99.6%) TENG communicator with a conductive hydrogel and a chemically modified elastomer. Reproduced with permission from ref 321. Copyright 2018 Springer Nature under CC BY 4.0 http://creativecommons.org/licenses/by/4.0/. (b) TENG using self-healing (∼95%) and nondrying conductive organohydrogel. Reproduced with permission from ref 36. Copyright 2021 Elsevier. (c) A highly conductive and stretchable click-ionogel-based wearable TENG application operable over a wide temperature range (−75 to 340 °C). Reproduced with permission from ref 329. Copyright 2019 The American Association for the Advancement of Science under CC BY 4.0 http://creativecommons.org/licenses/by/4.0/. (d) Wearable and battery-free TENG ionic patch for rapid wound healing. Reproduced with permission from ref 124. Copyright 2021 Elsevier.

To address the dehydration and freezing issues associated with hydrogels, Huang et al. presented a flexible and environment-resistant ionic TENG utilizing an ultrafast self-healing, nondrying conductive organohydrogel (Figure 17b).^36^ They incorporated organohydrogels, which remain stable across a wide temperature range (−30 to 80 °C) due to their hydrophobic and icephobic properties. The fabrication process involves solvent exchange, where water in a pAAm–clay hydrogel is replaced with glycerol, ensuring long-term conductivity and nondrying behavior. This design significantly enhances electrical output stability compared to that of traditional hydrogel-based TENGs. The organohydrogel-based TENG exhibits remarkable functionalities, including rapid self-healing (within 1 s), high mechanical durability, and stable electrical performance even under harsh environmental conditions. The developed system has been successfully integrated into self-powered sensors for biomechanical energy harvesting, such as motion detection in wearable electronics.

A flexible ionic conductor that operates stably over a wide temperature range is highly desirable for energy generation applications. A gel-based ionic TENG utilizing the low vapor pressure of an IL was developed, offering exceptional conductivity, mechanical resilience, and broad thermal stability (Figure 17c).^329^ The ionogel was synthesized via thiol–ene click chemistry, forming a dual-network structure that enhances both the ionic conductivity and mechanical strength. This structure enables the ionogel to remain highly stretchable, transparent, and resistant to freezing and heat degradation across a wide temperature range (−75 to 340 °C), ensuring stable electrical performance. These properties allow the click-ionogel to serve as a highly efficient electrode in flexible TENGs, enabling energy harvesting from mechanical stimuli. The developed TENG demonstrated robust mechanical and electrochemical performance under extreme conditions.

The soft, motion-driven nature of gel-based TENGs makes them highly suitable for integration into wearable systems. Accordingly, Jeong et al. developed a soft wound healing patch utilizing the battery-free mechanism of an ionic TENG as a wearable electrotherapeutic device. (Figure 17d).^124^ The system consists of a tube-structured TENG fabricated by using ionically conductive and stretchable organogel fibers. This fully wearable and flexible device effectively converts biomechanical energy from body motion into an electric field for electrical stimulation therapy. The power-generating component consists of a woven ionic fabric, which enhances the potential generation and charge transfer efficiency. When the ionic patch is applied to a wound site, it provides a uniform and symmetrical electric field, directly stimulating the wound. This triboelectric-driven electric field promotes fibroblast migration, angiogenesis, and collagen synthesis, thereby accelerating wound healing.

### 4.2 Gel-Based Thermoelectric Generators (TEGs)

Ionic thermoelectric generators (i-TEGs) utilize the movement of mobile ions to convert thermal gradients into electrical energy.^330^ Gel-based i-TEGs operate through ion-based charge transport mechanisms, offering high flexibility, stretchability, and compatibility with soft and biological environments.^331–334^ These systems primarily exploit two mechanisms: thermodiffusion (Soret effect) and thermogalvanic reactions. In the thermodiffusion mechanism, a temperature gradient induces the migration of ions from the hot side to the cold side, leading to charge separation and the generation of a thermovoltage (Figure 18a).^335–339^ The efficiency of this process is characterized by the ionic Seebeck coefficient, which can reach values significantly higher than those of traditional electronic thermoelectric materials. In contrast to purely diffusion-based processes, thermogalvanic mechanisms leverage redox reactions for a sustained voltage output. The thermogalvanic mechanism relies on redox species within the gel undergoing temperature-dependent electrochemical reactions at electrodes with different temperatures (Figure 18b).^340–343^ These reactions drive a continuous voltage output as redox couples cycle between the oxidation and reduction states. While thermodiffusion relies on ion mobility, thermogalvanic systems depend on redox potential differences induced by temperature gradients. Recently, synergistic approaches combining both thermodiffusion and thermogalvanic effects have been explored to enhance power output and stability.^344,345^ These gel-based i-TEGs offer promising opportunities for wearable thermal energy harvesting, self-powered bioelectronic devices, and flexible sensors, providing a sustainable alternative to conventional thermoelectric materials.

**Figure 18:**
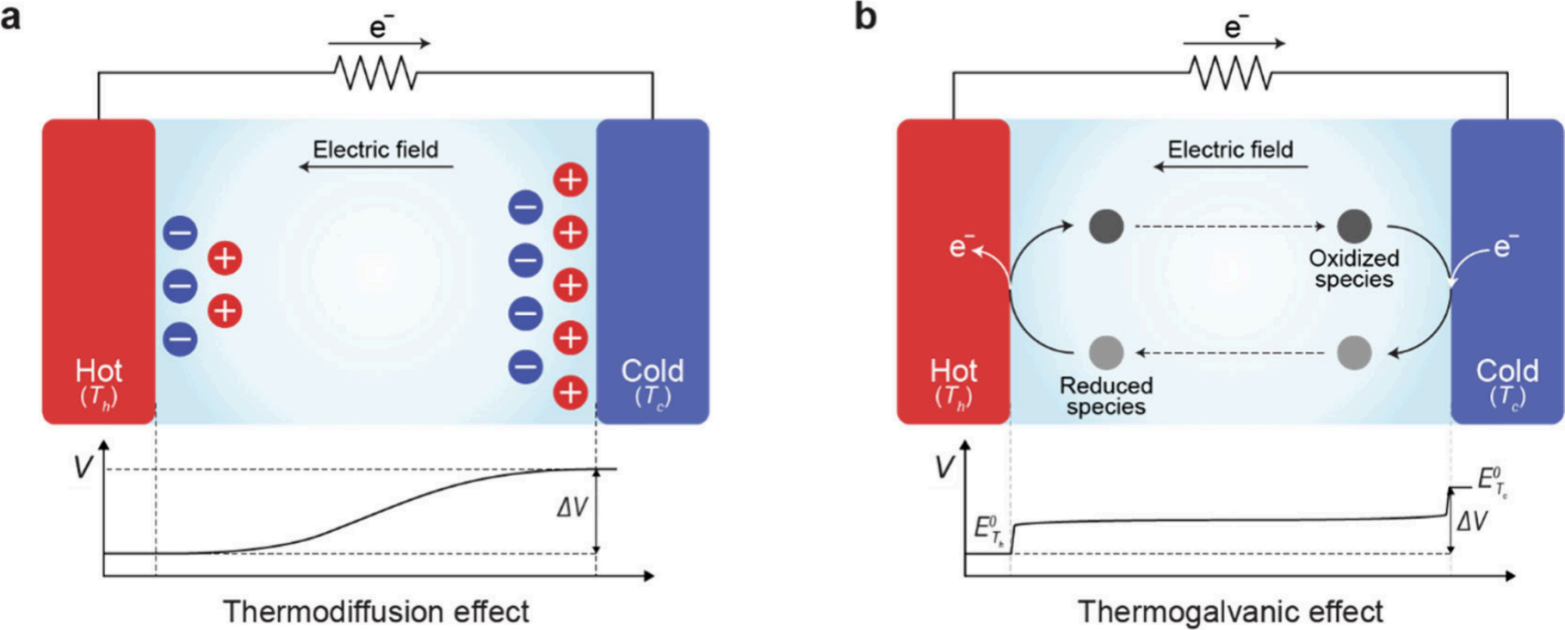
The two major mechanisms of thermoelectric generator (TEG). (a) The thermodiffusion effect caused by differences in ion mobility under a temperature gradient. (b) The thermogalvanic effect involving redox reactions at electrodes with different temperatures.

A thermoelectric generator (TEG) based on thermodiffusion is classified as either n-type or p-type, depending on the dominant ionic species responsible for charge transport. Recently, a gel-based system capable of bidirectional and highly efficient thermoelectric generation within an ionogel has been reported. This advancement was achieved by doping ions that selectively interact with pre-existing cations or anions (Figure 19a).^346^ They successfully modulated thermopower between −15 mV K^–1^ and +17 mV K^–1^, making it one of the best-performing n-type ionic thermoelectric materials. The key mechanism behind this tunability is the introduction of strong ion–ion interactions by doping Li^+^ for n-type behavior and Cl^–^ for p-type behavior into an ionogel matrix. Depending on the choice of dopants, cations or anions can dominate thermodiffusion, thereby controlling the direction of the thermopower. The use of ionogels instead of traditional liquid electrolytes provides enhanced thermal and mechanical stability, making them highly suitable for flexible and wearable thermoelectric applications. The researchers demonstrated a prototype wearable ionic thermoelectric device integrating 12 p-n pairs, achieving a total thermopower of 0.358 V K^–1^.

**Figure 19:**
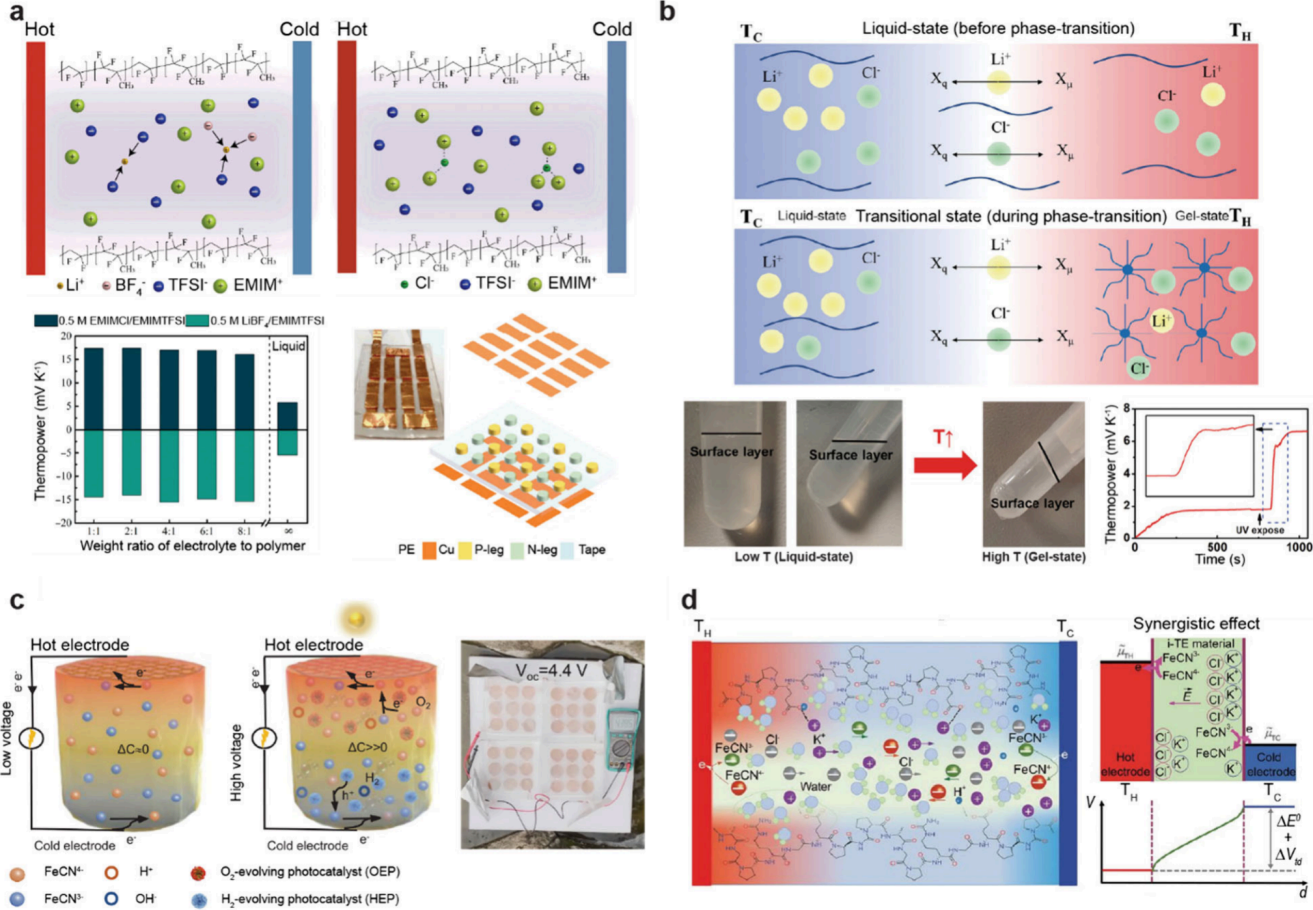
The gel-based thermoelectric generators with distinct ionic properties. (a) Bidirectionally tunable thermopower enabled by selective ion doping. Reproduced with permission from ref 346. Copyright 2022 The American Association for the Advancement of Science under CC BY 4.0 http://creativecommons.org/licenses/by/4.0/. (b) Modulated thermopower with ion mobility controlled by a phase-transition matrix. Reproduced with permission from ref 347. Copyright 2022 John Wiley and Sons. (c) Effective thermogalvanic properties in photocatalyst-doped hydrogels that have a high ion concentration difference. Reproduced with permission from ref 348. Copyright 2023 The American Association for the Advancement of Science. (d) Enhanced thermopower with synergistic thermodiffusion and thermogalvanic effect. Reproduced with permission from ref 345. Copyright 2020 The American Association for the Advancement of Science.

Physically cross-linked hydrogels, such as gelatin and agarose gels, undergo temperature-responsive sol–gel transitions, altering ion mobility. Utilizing this mobility difference in a phase-transitioning matrix, a significant enhancement in thermopower was reported (Figure 19b).^347^ The study revealed that a sol-to-gel transition in the poloxamer/LiCl i-TEG system induces a 6.5-fold increase in thermopower and a 23-fold enhancement in the ionic figure of merit. This drastic improvement is attributed to changes in ion transport properties, including alterations in ion diffusion and thermodiffusion behaviors during phase transition. The mechanism behind this enhancement is linked to the restructuring of the polymer network, which modulates the ion mobility, leading to increased ion separation under a temperature gradient. Instead of using stable gels or liquid electrolytes, this study emphasizes the potential of phase-transition-induced thermoelectricity for tunable energy harvesting.

A gel-based thermogalvanic cell incorporating a photocatalyst was reported, utilizing a continuously regulated and sustained redox ion concentration gradient to achieve enhanced thermoelectric performance (Figure 19c).^348^ The system integrates a hydrogel matrix incorporating a redox couple (FeCN^4–^/FeCN^3–^) and photocatalysts for oxygen and hydrogen evolution. Under sunlight irradiation, the photocatalysts enhance the conversion of FeCN^3–^ to FeCN^4–^ at the hot side and FeCN^4–^ to FeCN^3–^ at the cold side, generating a continuous ion concentration gradient. This mechanism enhances the thermopower to 8.2 mV K^–1^, significantly outperforming conventional thermogalvanic systems. The hydrogel-based platform offers stable ion migration, improved mechanical integrity, and enhanced light absorption, making it suitable for large-area and scalable energy harvesting. The simultaneous generation of electricity and hydrogen further highlights the potential of this system for renewable energy applications. By integrating photocatalysis with thermogalvanic energy conversion, this approach paves the way for sustainable and efficient thermal-to-electrical energy harvesting.

Han et al. demonstrated the first synergistic ionic thermoelectric generator that combines thermodiffusion and thermogalvanic effects to achieve exceptionally high thermopower within a gel matrix (Figure 19d).^345^ By incorporating ion providers (KCl, NaCl, and KNO_3_) for thermodiffusion and a redox couple [Fe­(CN)_6_^4–^/Fe­(CN)_6_^3–^] for thermogalvanic conversion, they achieved a thermopower of 17.0 mV K^–1^. The system operates through a synergistic mechanism, where thermodiffusion creates an initial charge separation under a temperature gradient, while the redox reaction at the electrodes sustains continuous electron flow. The gel-based structure provides mechanical flexibility and long-term stability, making it suitable for wearable energy harvesting. A proof-of-concept wearable device composed of 25 unipolar elements generated over 2 V using body heat, achieving a peak power of 5 μW. This work highlights the potential of ionic thermoelectric materials for self-powered electronics, particularly in flexible and wearable applications.

### 4.3 Gel-Based Concentration-Driven Power Generators

Ionic systems have also been explored for concentration-driven energy harvesting utilizing ion transport mechanisms across selective membranes within gel electrolytes. These systems generate electrical power from ion concentration gradients, similar to the biological processes observed in electric eels. Voltage generation in gel-based ionic systems occurs through ion concentration gradients between high- and low-salinity gels, facilitated by cation- and anion-selective membranes (Figure 20a). When these two gels are placed in contact with selective membranes, cations preferentially migrate through the cation-selective membrane, while anions move through the anion-selective membrane, creating charge separation (Figure 20b). The primary mechanisms driving ionic conduction in these systems include diffusion-driven ion transport, reverse electrodialysis, and Donnan potential effects, all of which contribute to voltage generation. Cation- and anion-selective membranes play crucial roles in directing ionic flow, maintaining charge separation, and enabling continuous power output. Based on these principles, various applications have been developed, including bioinspired soft power sources,^7,349,350^ implantable ionic energy harvesters,^49,351,352^ and flexible hydrogel-based power sources for wearable electronics.^25,353,354^

**Figure 20:**
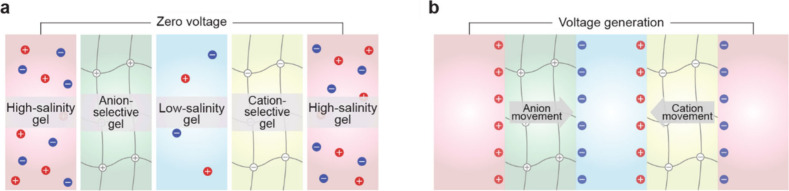
A power source driven by an ion concentration gradient. (a) Representative configuration of a power source based on an ion concentration gradient. (b) When they come into physical contact, the potential is generated by the ion-selective membranes.

Schroeder et al. present the first study on a soft, gel-based ionic power source inspired by the electric eel, utilizing ion concentration gradients to generate electricity (Figure 21a).^7^ The system consists of stacked pAAm hydrogel compartments separated by alternating cation- and anion-selective membranes, mimicking the natural electrocyte arrangement in electric eels (Figure 21b). When mechanical contact is applied, ionic gradients across the selective membranes create electrochemical potential differences, enabling voltage generation through the principle of reverse electrodialysis. This scalable system achieves an open-circuit voltage of 110 V and a power output of 27 mW m^–2^. The hydrogel-based architecture offers key advantages, including flexibility, transparency, and potential biocompatibility, making it suitable for integration into bioelectronic applications (Figure 21c). Additionally, the simple fabrication process allows for scalable stacking or folding strategies, enabling the formation of large-area ionic power sources.

**Figure 21:**
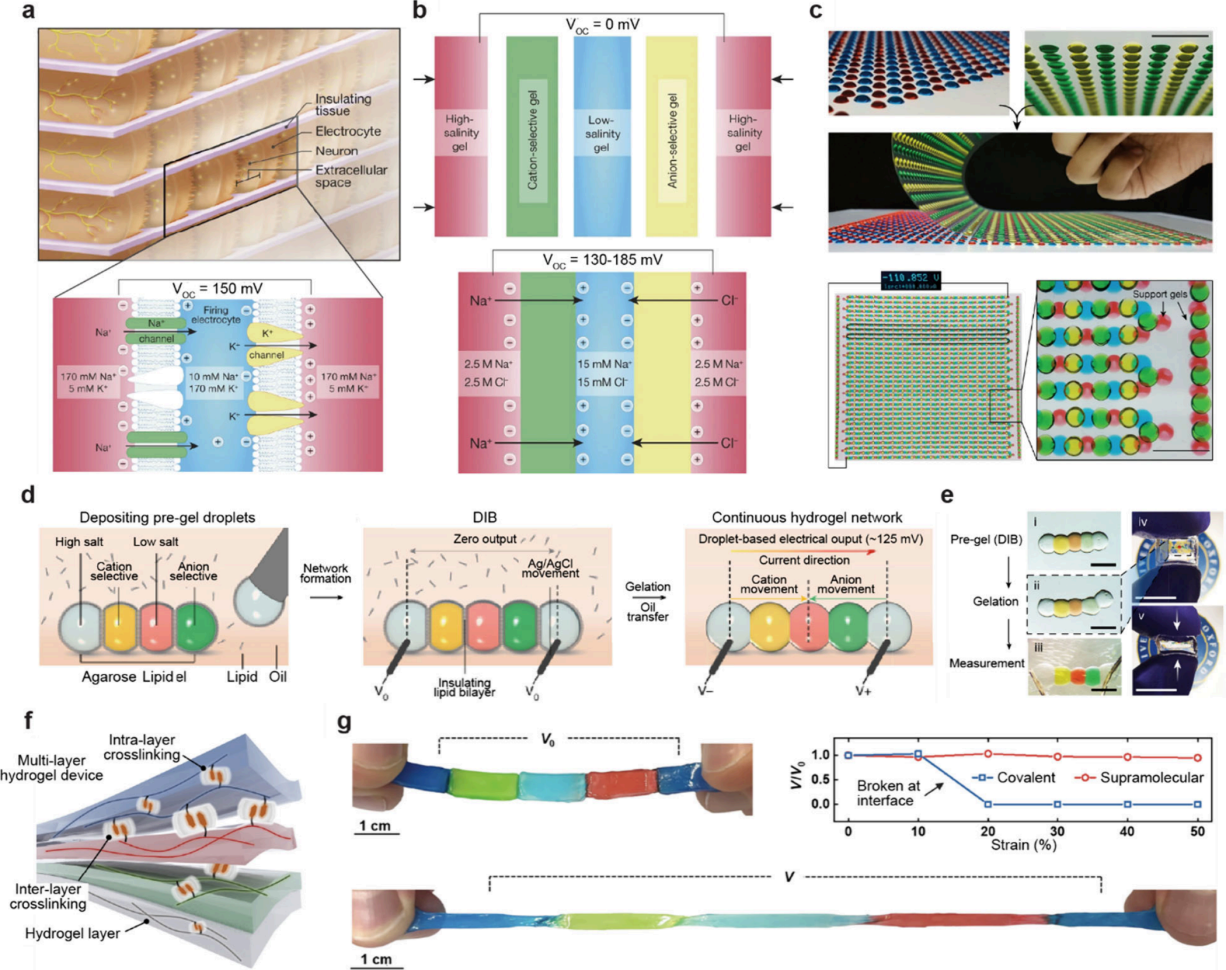
Power sources using ion-selective membranes and gels. (a–c) Hydrogel-based soft power source with an ion-selective membrane inspired by an electric eel. Reproduced with permission from ref 7. Copyright 2017 Springer Nature. (a) Structure of electric organs of electric eel. (b) Artificial design of a hydrogel power source. (c) A hydrogel power source printed over a large area. (d,e) A microscale soft ionic power source. Reproduced with permission from ref 49. Copyright 2023 Springer Nature under CC BY 4.0 http://creativecommons.org/licenses/by/4.0/. (d) A process of power generation using a microscale soft ionic power source. (e) Image of a microscale soft ionic power source. (f,g) Highly stretchable hydrogel power source. Reproduced with permission from ref 25. Copyright 2024 The American Association for the Advancement of Science under CC BY 4.0 http://creativecommons.org/licenses/by/4.0/. (f) Host–guest interaction-based supramolecular cross-links for high stretchability. (g) Stable voltage generation under applied strain using a hydrogel power source.

Microscale soft ionic power sources are essential for biointegrated devices, since conventional options like bulky batteries or wireless power transfer are often inefficient for cellular-scale stimulation. By leveraging ion gradients within a soft, biocompatible hydrogel network, a miniaturized ionic power source can store and deliver energy efficiently, enabling precise neuronal modulation and on-demand operation in biological environments (Figure 21d).^49^ The system consists of hydrogel droplets containing alternating high- and low-salt compartments separated by cation- and anion-selective interfaces. When activated, ions migrate through these selective membranes, creating electrochemical potential differences that enable power generation. This hydrogel-based system offers the advantage of scalability and simple fabrication, enabling the creation of miniaturized, yet efficient, ionic power units (Figure 21e). This microscale platform enables biocompatibility and direct interfacing with biological systems. This system was successfully applied to neuromodulation, where the ionic currents generated by the hydrogel droplets modulated neuronal activity in 3D neural microtissues and ex vivo brain slices.

A highly stretchable hydrogel power source is essential for bioelectronics and soft robotics, where devices must maintain a high ionic conductivity while enduring extreme mechanical deformations. O’Neill et al. developed a highly stretchable gel-based ionic power source by incorporating supramolecular poly­(ionic) networks that provide both high ionic conductivity and exceptional mechanical resilience (Figure 21f).^25^ The dynamic supramolecular cross-links enable stable ionic conduction even under large mechanical deformations (Figure 21g). The supramolecular networks ensure superior interfacial adhesion between layers, enabling a fully stretchable, multilayered hydrogel power source. The supramolecular design allows the power source to maintain consistent electrical performance even when stretched up to 50% strain, unlike covalently cross-linked hydrogels, which suffer from interfacial fracture under mechanical stress. Its combination of high stretchability (>1500%), rapid self-recovery, and ionic conductivity (up to 0.1 S cm^–1^) makes it a promising candidate for bioelectronic devices.

### 4.4 Gel-Based Piezoionic Power Generators

Ions in gel matrices exhibit unique interactions with their surroundings, enabling efficient mechanoionic energy conversion. The gel environment provides a highly tunable platform where ion mobility (Figure 22a),^355,356^ selective migration (Figure 22b),^266,355^ and dynamic trapping-release mechanisms (Figure 22c)^357–360^ can be precisely regulated by the polymer network properties. Piezoionic generation is fundamentally driven by ion movement in response to mechanical force. Some systems rely on a direct ion migration difference, where mobile ions shift within the gel matrix due to pressure gradients, generating an ionic current. Others utilize selective ion migration, where specific cations or anions preferentially migrate through a functionalized polymer network, creating a directional charge flow. Additionally, ion trapping and release strategies enable dynamic ionic responses by mimicking biological mechanoreceptors by storing mechanical energy before it is released as an electrical signal. Gel-based ionic generators demonstrate outstanding flexibility, self-healing properties, and biocompatibility, making them ideal for applications such as self-powered tactile sensors, artificial mechanoreceptors, wearable piezoionic harvesters, and sensory skins for human–machine interfacing.

**Figure 22:**
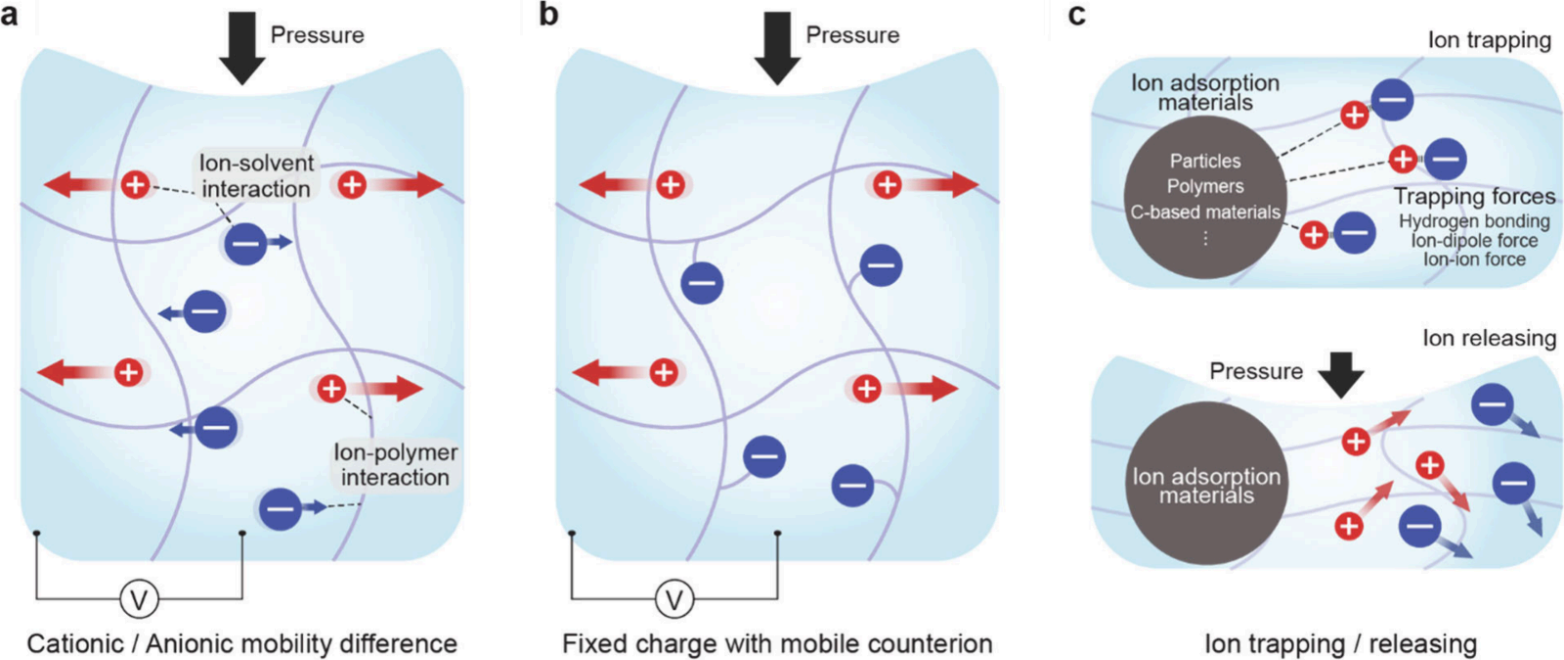
The fundamental mechanisms and various strategies of piezoionics. (a) Potential generation in a neutral gel by an externally induced force. (b) Potential generation with fixed charges and mobile counterions. (c) Methods of ion trapping and releasing through various interactions and materials.

Human tactile perception relies on soft mechanoreceptors that interact seamlessly with neural tissues by using ionic currents to sense and process stimuli. To replicate this, piezoionic mechanoreceptors offer a biocompatible, self-powered alternative to traditional electronic sensors by directly converting mechanical pressure into ionic currents. Dobashi et al. reported piezoionic mechanoreceptors that generate force-induced ionic currents in hydrogels, mimicking biological sensory systems (Figure 23a).^355^ The mechanism involves pressure-driven ion migration, where asymmetric ionic transport occurs through a charged hydrogel matrix. The magnitude and polarity of the generated voltage are determined by cationic and anionic mobility differences, influenced by the polymer content and ionic species. The study shows that polymer networks regulate ion mobility with lower polymer content leading to faster transient responses. Additionally, introducing fixed charges within the hydrogel enhances voltage generation by creating built-in potential differences. This piezoionic approach was applied to artificial mechanoreceptors and neural interfaces, demonstrating direct neuromodulation and muscle excitation.

**Figure 23:**
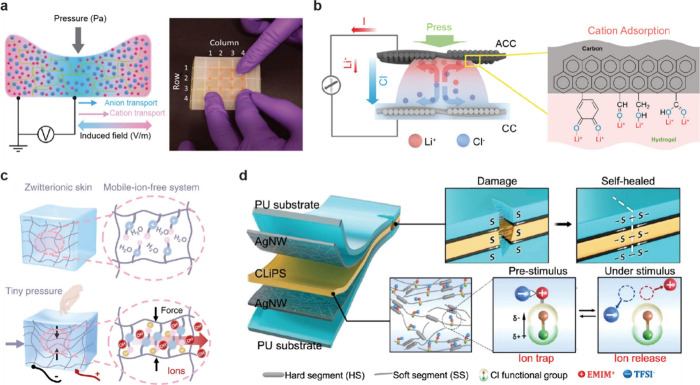
Various piezoionic devices utilizing ion selectivity and gel properties. (a) Piezoionic sensor mimicking the principle of biological tactile perception. Reproduced with permission from ref 355. Copyright 2022 American Association for the Advancement of Science. (b) High-current power source with selective ion adsorption and a pyramid-structured hydrogel. Reproduced with permission from ref 361. Copyright 2024 Springer Nature under CC BY 4.0 http://creativecommons.org/licenses/by/4.0/. (c) Force-induced ion generation sensing in a zwitterionic hydrogel. Reproduced with permission from ref 64. Copyright 2023 Springer Nature under CC BY 4.0 http://creativecommons.org/licenses/by/4.0/. (d) Ion-dipole interaction for ion trapping in a self-healable ionogel. Reproduced with permission from ref 357. Copyright 2022 Springer Nature under CC BY 4.0 http://creativecommons.org/licenses/by/4.0/.

The piezoionic devices rely on the movement of ions under mechanical stress, producing a charge density significantly higher than that of conventional piezoelectric and triboelectric sensors. Building on this advantage, a high-current hydrogel generator was developed to achieve amplified piezoionic electricity generation through engineered structural and chemical asymmetry (Figure 23b).^361^ By the design of a pyramid-structured hydrogel, the system induces a high-strain gradient under mechanical compression. This structure leads to an unbalanced diffusion of cations and anions, generating a net ionic current. This effect is further enhanced by the asymmetric ion adsorption properties of the electrodes, where an activated carbon cloth electrode with abundant oxygen functional groups selectively anchors cations, intensifying charge separation. The hydrogel generator demonstrates a significantly improved output, achieving 4 mA (5.5 A/m^2^) under cyclic compression. These results demonstrate the effectiveness of the asymmetric design. The generator was successfully integrated into a self-powered drug-releasing system, where the generated ionic current triggered the controlled release of an antibiotic from a drug-reservoir layer, demonstrating its potential for biomedical applications.

Zwitterionic hydrogels, with superior ion transport properties compared to nonionic hydrogels, also have been explored for mimicking the human sensory system. The study by Xu et al. introduces a force-induced ion generation mechanism in zwitterionic hydrogels (Figure 23c).^64^ In this system, external pressure reduces the distance between the zwitterionic groups, leading to water dissociation. This effect was confirmed through experimental pH measurements and density functional theory calculations. The results demonstrate that Coulomb interactions between zwitterionic segments facilitate water dissociation into hydroxide ions (OH^–^), thereby increasing ionic conductivity under mechanical stimuli. Compared with traditional ionic systems, the zwitterionic hydrogel inherently enables ion transport without the need for additional ionic species. The zwitterionic polymer chains create continuous migration channels for the dissociated ions, allowing for efficient charge transport. This unique ion transport mechanism enhances signal sensitivity, making the hydrogel five times more responsive than nonionic hydrogels. Furthermore, this zwitterionic hydrogel mimics the mechanoelectrical response of natural skin, exhibiting a rapid response time (∼38 ms), comparable to biological mechanoreceptors.

A new piezoionic mechanism has recently been reported where ions are trapped through various interactions and released in response to mechanical stimuli. Boahen et al. reported a Cl-functionalized iontronic pressure-sensitive material, designed by incorporating Cl-functionalized groups into a polyurethane matrix (Figure 23d).^357^ This system achieves ultrafast, autonomous self-healing and exhibits mechanosensitive piezoionic dynamics. The Cl groups in the backbone chain play a crucial role in trapping and releasing ions through ion–dipole interactions, enabling efficient pressure sensitivity. Under external pressure, the trapped ions are released, forming an EDL and generating a highly responsive piezocapacitive effect. Their iontronic skin demonstrates exceptional self-healing efficiency (91% within 60 min), rapid healing speed (4.3 μm/min), and outstanding elastic recovery (100%). Compared with conventional covalent bonding approaches, the supramolecular interactions in this system ensure consistent performance under mechanical strain. Additionally, the incorporation of an IL enhances self-healing through plasticization effects and contributes to stable ionic conduction. As a practical demonstration, this electronic skin was integrated into a tactile sensing system, exhibiting high sensitivity (7.36 kPa^–1^) in response to pressure variations.

### 4.5 Gel-Based Moisture Electricity Generators (MEGs)

Moisture-electric generators (MEGs), which utilize ionic conduction, have emerged as a promising strategy for harvesting sustainable energy from atmospheric moisture (Figure 24).^362–364^ The fundamental mechanism of MEGs involves three key processes: moisture adsorption, ion dissociation, and ion diffusion. In moisture adsorption, hygroscopic gels capture water molecules from the environment, creating localized hydration layers that facilitate ion transport (Figure 24a). Subsequently, ion dissociation occurs as absorbed water molecules interact with functional groups in the gel matrix, releasing mobile charge carriers, such as protons or other ions (Figure 24b). Finally, ion diffusion drives charge separation and current generation as the asymmetric moisture gradient across the hydrogel induces directional ion migration, forming an electric potential (Figure 24c). Gel-based MEGs offer several advantages over conventional moisture-electric systems. Their high water-retention capacity ensures prolonged operation, while their stretchability and mechanical flexibility make them ideal for wearable and implantable applications.^365–369^ Additionally, hydrogel-based MEGs demonstrate scalable fabrication potential, allowing for large-area integration and improved power density.^370,371^ Recent advancements have demonstrated significant improvements in electrical output, with optimized gel compositions achieving stable voltage generation over extended periods.^372,373^

**Figure 24:**
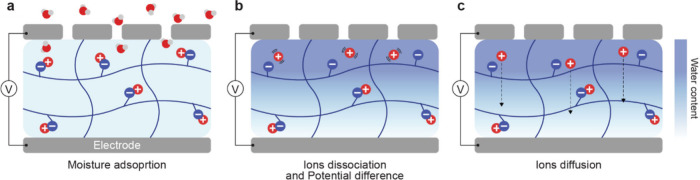
Representative processes of the moisture electricity generator mechanism. (a) Anisotropic water adsorption through a water-permeable electrode. (b) Ion dissociation by adsorbed water molecules. (c) Ion diffusion driven by ion concentration difference.

The working duration of an ionic-system-based energy generator is determined by the maintenance of the ion concentration gradient. Extending this limited duration has been a significant challenge. Recently, MEG that sustains long-term green energy generation has been developed by Duan et al. (Figure 25a).^374^ The system integrates a photocatalytically enhanced hydrovoltaic effect to reconstruct ion concentration gradients, which are crucial for a continuous current output. The moisture-enabled electricity generation layer consists of a pAAm-based hydrogel enriched with hydrophilic functional groups that facilitate efficient moisture adsorption. This process leads to ion dissociation and migration, generating voltage through the hydrovoltaic effect (Figure 25b). Over time, the ion concentration gradient diminishes due to ion migration saturation. To resolve this, a photocatalytic layer is introduced using light energy to drive hydrogen evolution reactions. The photocatalysis depletes prestacked hydrogen ions, re-establishing the ion concentration gradient and allowing the MEG to sustain electrical output over extended periods. This innovative integration of photocatalysis significantly enhances the lifespan of MEGs, achieving continuous operation (over 600 h). Additionally, the gel-based design ensures scalability, ease of fabrication, and the potential for multifunctional integration.

**Figure 25:**
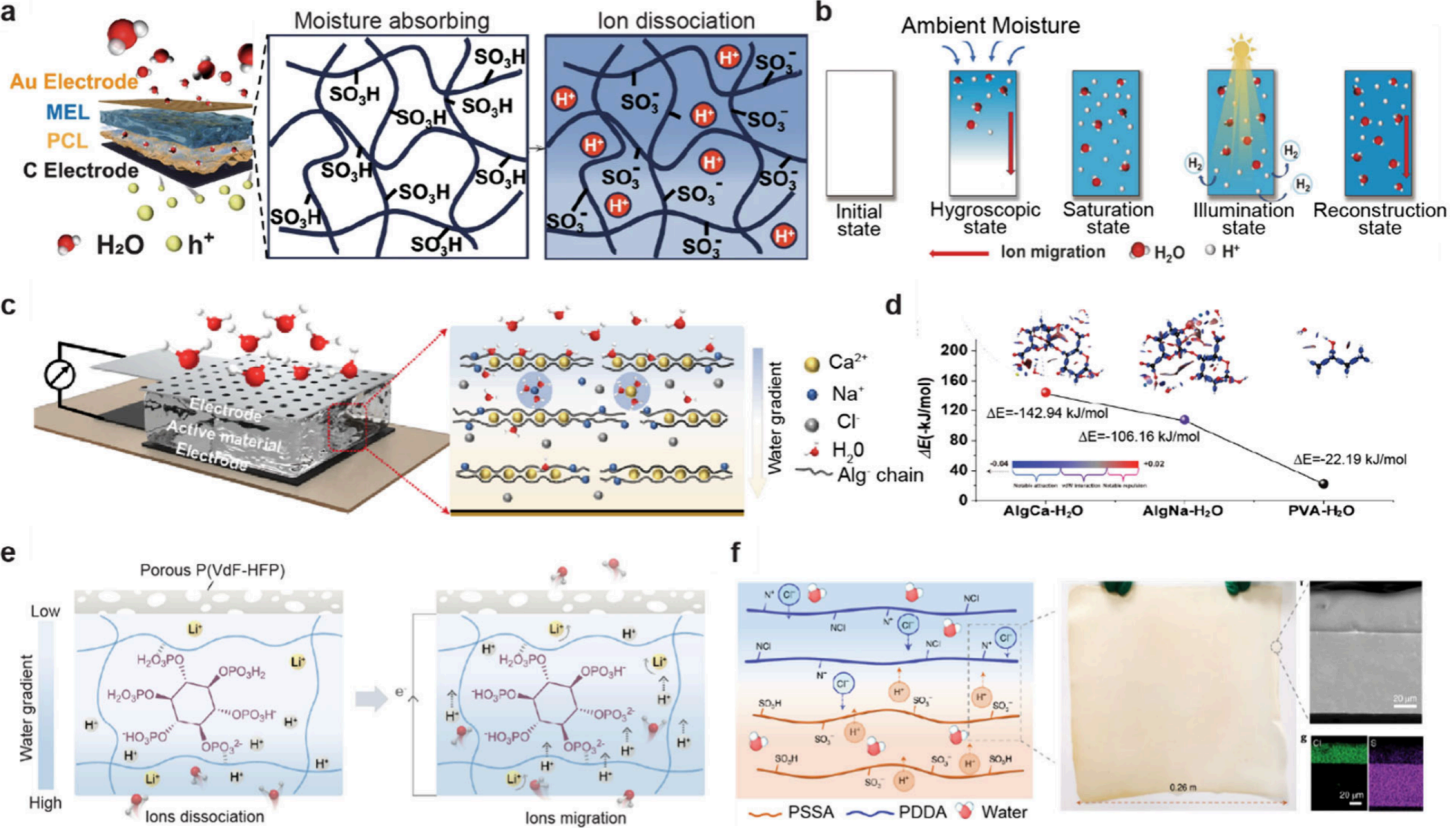
Various strategies for moisture electricity generation. (a,b) Moisture-enabled electric generator using a hygroscopic gel. Reproduced from ref 374. Copyright 2025 Springer Nature under CC BY-NC-ND 4.0 https://creativecommons.org/licenses/by-nc-nd/4.0/. (a) Ion dissociation through moisture absorption in a hygroscopic gel. (b) Process of ion migration in hydrogel with photocatalytic effect. (c,d) Hydrogel based moisture-electric generator. Reproduced with permission from ref 375. Copyright 2024 Springer Nature under CC BY 4.0 http://creativecommons.org/licenses/by/4.0/. (c) Enhanced water absorption through a hygroscopic network. (d) Quantitatively calculated adsorption energy of various polymers and water molecules. (e) Selective ion transportation driven by directed water flow. Reproduced with permission from ref 376. Copyright 2024 Springer Nature under CC BY 4.0 http://creativecommons.org/licenses/by/4.0/. (f) Large-scale bilayer of polyelectrolyte film for moisture-enabled electric generation. Reproduced with permission from ref 371. Copyright 2021 Springer Nature.

The working mechanism of MEGs relies on the interaction between water molecules and the polymer network within a gel. Recent studies have attempted to quantitatively analyze these interactions based on different materials, providing deeper insights into optimizing MEG performance. Yang et al. designed the gel-based MEG by exploiting strong interactions between water molecules and ionic cross-linkers (Ca^2+^/Na^+^) in a PVA–AlgNa supramolecular hydrogel (Figure 25c).^375^ These ionic sites strongly bind water, facilitating rapid moisture uptake and forming ion–water clusters that diffuse at a slower rate. The resulting persistent water gradient drives continuous ion migration, producing a stable DC output with milliampere-level current and volt-scale voltage. Through density functional theory calculations and experimental analyses, the researchers quantitatively verified the generator’s enhanced moisture adsorption and controlled ion transport capabilities, confirming its superior MEG performance (Figure 25d). Consequently, the PVA–AlgNa–CaCl_2_ hydrogel not only functions as an efficient water-adsorption matrix but also serves as a robust medium for ion conduction.

Guo et al. developed a self-sustaining and highly efficient MEG utilizing a bilayer polymer structure for continuous power generation under ambient environmental fluctuations (Figure 25e).^376^ The system employs a radiative cooling-assisted strategy to maintain a dynamic sorption–desorption equilibrium, ensuring stable ion transport and extended power output. The top layer, consisting of a hydrophobic and porous PVDF-*co*-HFP film, minimizes daytime moisture loss by reflecting solar radiation, while enhancing nighttime moisture uptake through radiative cooling. This effect prevents excessive evaporation during the day and accelerates water absorption at night, sustaining a continuous directed water/ion flow. In the hygroscopic layer, LiCl disrupts hydrogen bonding within the hydrogel, increasing the mobility of dissociated ions and facilitating rapid charge separation. Additionally, the strong interaction between phytic acid and Li^+^ enhances ion conductivity by expanding ion transport pathways within the gel matrix. The device demonstrated continuous power generation for over 6 days in outdoor conditions, proving its robustness for real-world applications.

Wang et al. developed a heterogeneous moisture-electric generator utilizing a bilayer of polyelectrolyte films for spontaneous power generation in ambient air (Figure 25f).^371^ The key mechanism involves the asymmetric distribution of mobile ions within the polyelectrolyte layers, leading to continuous ion diffusion and voltage generation. The system, where moisture absorption triggers ion dissociation, consists of a pDADMAC polycation layer and a pSSA polyanion layer. The resulting concentration gradient drives the opposite migration of Cl^–^ and H^+^ ions, inducing an electric potential. This design efficiently harnesses ambient humidity to generate a stable voltage output of ∼0.95 V at 25% relative humidity, which further increases to 1.38 V at 85% relative humidity. By employing a scalable stacking strategy, the researchers successfully integrated multiple units. Moreover, the processing method based on a simple casting and spraying technique, allows a large-area fabrication.

The use of ionic charge carriers in gel-based power sources enables unique energy harvesting mechanisms that are inherently compatible with soft, deformable, and biointegrated systems. These include triboelectric generation, thermoelectric conversion, concentration-driven processes, piezoionic effects, and moisture-induced electricity, each offering operational advantages and design flexibility. A summary of their electrical performance characteristics is provided in Table 2. Gel-based TENGs achieve mechanical-to-electrical conversion via contact electrification and are notable for high output voltage and compatibility with transparent and stretchable designs. Gel-based TEGs, while exhibiting lower power output, offer continuous energy generation driven by temperature gradients and can be stably operated using ionogels in thermally challenging environments. Concentration-driven power generators utilize ion-selective membranes and spontaneous chemical potential gradients, enabling long-term operation without mechanical or thermal stimuli. Piezoionic power generators convert pressure changes into ionic flux and generate current with minimal structural complexity, while MEGs exploit humidity gradients and moisture diffusion, providing battery-free and self-sustaining energy output.

**Table 2:** Key Characteristics and Applications of Gel-Based Ionic Power Sources

Type	Driving mechanism	Power density	Advantages in gel-based system	Representative applications
Triboelectric Nanogenerators	Contact electrification	∼mW/cm^2^	High voltage, transparency, stretchability	Touch sensors, wearables
Thermoelectric Generators	Thermal gradient	∼μW/cm^2^	Continuous operation, ionogel-based thermal resilience	Skin-mounted heat harvesters
**Concentration-Driven Power Generators**	Concentration gradient	∼μW/cm^2^	No external stimuli, long-term passive output	Implantables, environmental sensors
**Piezoionic Power Generators**	Pressure-induced ion flow	∼μW/cm^2^	Simple structure, fast response	Pressure sensors, self-powered tactile sensing
**Moisture Electricity Generators**	Moisture gradient	μW ∼ mW/cm^2^	Ambient energy harvesting, green energy harvesting	Breath/humidity sensors

Compared with conventional electronic energy harvesters, these gel-based ionic systems offer distinct advantages in both material and functional domains. Their intrinsic softness, conformability, and chemical responsiveness allow seamless integration into living tissues, wearable devices, and soft robotic systems, where rigid electronics are impractical. Moreover, ionic conduction mechanisms support multifunctional device architectures, enabling simultaneous sensing and energy harvesting within a single platform. These characteristics collectively expand the design of next-generation power generators. In addition, gel-based systems can be fabricated with low-cost materials, patterned into arbitrary geometries, and reliably coupled with stretchable components.^124,321,377^

## 5 Noncircuit Elements

### 5.1 Electro-osmosis

#### 5.1.1 Mechanism of Electro-osmosis

Electro-osmosis refers to the collective movement of a liquid under an externally applied electric field, based on the interaction between a charged solid surface and ions within the liquid.^378–382^ Typically, the solid surface carries either a negative or a positive charge, causing oppositely charged ions in the fluid to accumulate near the surface and form an EDL. The EDL itself is often described in two parts. The first part, known as the Stern layer (or fixed layer), is a thin region in which ions are strongly bound to the charged surface and remain essentially immobile. Beyond this fixed layer lies the diffuse layer, where ions can move relatively freely. The thickness of this region, commonly called the Debye length, depends on factors, such as electrolyte concentration and temperature.

When an external electric field is applied, ions in the diffuse layer beyond the shear plane experience an electrostatic force and begin to move. Since these ions are hydrated and subject to viscous interactions with surrounding solvent molecules, they drag parts of the liquid with them, resulting in a collective flow of the fluid (Figure 26). Since neutral molecules do not experience a direct electric force and free electrons in an aqueous solution rapidly react with other substances, only the ions within the diffuse layer actually drive electro-osmosis.

**Figure 26:**
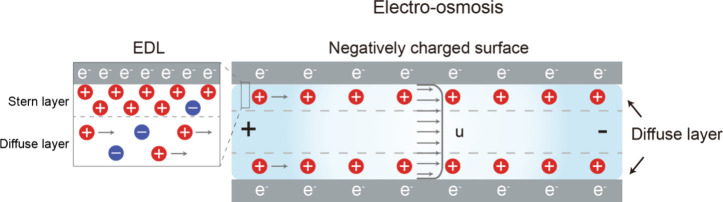
A mechanism of electro-osmosis. Due to the formation of an EDL, counterions are gathered at the diffuse layer. When an electric field is applied, these ions move, causing the surrounding solution to flow in the same direction.

To describe this phenomenon quantitatively, the Smoluchowski equation is widely used. The electro-osmotic flow velocity (*V*_*eo*_) depends on several factors, including the electric field strength (*E*), the zeta potential (ζ), the permittivity (ε) of the medium, and its viscosity (η). Mathematically, this relationship is expressed in the following equation.

7 $$V_{eo}=-\frac{\varepsilon \zeta E}{\eta }$$

The zeta potential, which is the potential difference between the solid surface and the shear plane, plays a critical role: a higher zeta potential produces stronger electrostatic forces and thus faster fluid movement. In addition, the permittivity and viscosity of the liquid significantly influence the flow rate.

Electro-osmosis is an ion-driven transport phenomenon that offers distinct advantages due to the hydration characteristics of ions. Because hydrated ions possess finite mass and migrate collectively in response to an electric field, they exert a significantly greater dragging force on the surrounding fluid compared with the negligible influence of electron motion. As a result, electro-osmosis is particularly effective in inducing bulk fluid flow.^383,384^ This principle is commonly implemented by integrating electrodes on solid substrates or by engineering charge distributions at solid–liquid interfaces. Such mechanisms have been extensively utilized in microfluidic systems, where they serve as efficient and compact pumping strategies for directing fluids toward specific targets.^385–387^

#### 5.1.2 Gel-Based Electro-osmotic Systems with Unique Characteristics of Ions

As electro-osmosis offers a convenient way to electrically control the properties of gels that contain a large amount of solvent, it has been extensively studied in the field of gels. The movement of solvent via electro-osmosis was a significant breakthrough in overcoming the slow swelling of conventional gels. Na et al. used a polyanion hydrogel to form channels through which cations could migrate, thereby inducing electro-osmosis and achieving very rapid swelling (Figure 27a).^388^ By combining this gel with a blocking layer, they successfully developed a gel with an extremely high strength and force output. This approach overcame the weak force and slow speed of conventional gel-based actuators, even demonstrating the capacity of breaking bricks (Figure 27b).

**Figure 27:**
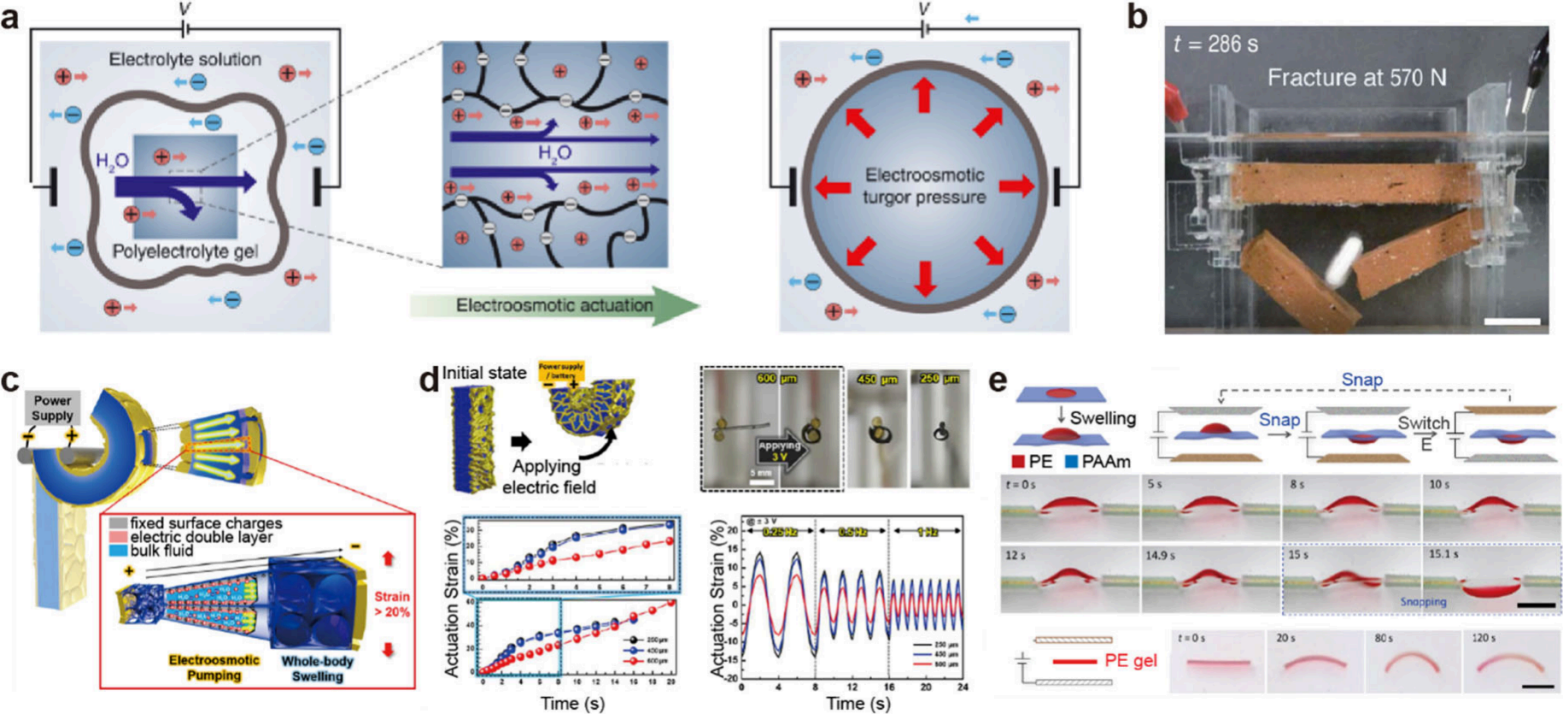
Electro-osmotic systems using gels. (a) Under an electric field, polyelectrolyte gels enable electro-osmosis, resulting in rapid swelling and thereby fast, strong actuation. (b) By utilizing electro-osmotic pressure along with the blocking force of the external layer, an actuation could be sufficiently strong to break bricks. Reproduced with permission from ref 388. Copyright 2022 The American Association for the Advancement of Science. (c) By harnessing the flexibility and shape-retainability of a hydrogel along with cracked electrodes, a high-energy density actuator could achieve diverse motion. Reproduced with permission from ref 389. Copyright 2020 American Chemical Society. (d) Wrinkled electrodes created by the gel’s deswelling process enhance both conductivity and mechanical flexibility, ultimately enabling insect-scale untethered soft aquabots that operate at voltages below 3 V. Reproduced with permission from ref 390. Copyright 2018 The American Association for the Advancement of Science. (e) Swelling mismatch and geometric confinement induce mechanical instability; when an electric field is applied, ions and solvents move rapidly, causing a snapping motion. Reproduced from ref 391. Copyright 2022 The American Association for the Advancement of Science under CC BY-NC 4.0 https://creativecommons.org/licenses/by-nc/4.0/

Electro-osmosis allows control over the degree of water transport by adjusting the electric field. When coupled with a gel’s inherent flexibility and shape-retainability, this mechanism can enable stable actuator performance. In work by Ko et al., cracked electrodes were integrated into a hydrogel, resulting in an actuator with a high energy density of about 1.06 × 10^5^ J/m^3^ and low power consumption of 4 mW/cm^2^ (Figure 27c).^389^ The cracked electrodes, created by assembling metal nanoparticles layer-by-layer in a nonpolar medium and then incorporating them into the hydrogel, enabled rapid electro-osmotic pumping. This system provides a wide range of possible motions. In subsequent research, Ko et al. further leveraged the concept of deswelling in a hydrogel to coat a wrinkled nanomembrane electrode onto the gel (Figure 27d).^390^ By employing electro-osmosis in this setup, they developed insect-scale untethered soft aquabots. This approach improved both the conductivity and mechanical flexibility of conventional hydrogel-based electrodes, allowing very high actuation performance even at voltages below 3 V. Specifically, it achieved a strain of over 50%, an energy density above 7 × 10^5^ J/m^3^, and a power density exceeding 3 × 10^4^ W/m^3^.

There have also been studies aimed at maximizing fluid flow driven by electro-osmosis to generate significant motion. In Figure 27e, for instance, a polyelectrolyte gel was bonded to a pAAm gel and bent upward to create a mechanically unstable state.^391^ Even a small input of energy was sufficient to trigger a snap-through to the next stable state. By applying an electric field, this “snap-through” process can be induced extremely quickly, showcasing another innovative example of rapid actuator behavior driven by electro-osmosis.

### 5.2 Ion–Polymer Metal Composite

#### 5.2.1 Mechanism of Ion–Polymer Metal Composite (IPMC)

Ion–Polymer Metal Composite (IPMC) is a composite material in which a gel network containing ions is combined with metal electrodes, such as platinum, gold, and silver.^392–397^ As a representative ionic electroactive polymer, it can generate large mechanical displacements at voltages below just a few volts. The polymer matrix and solvent form a gel that facilitates ion transport by creating conductive channels. Metal electrodes are typically deposited or coated in thin layers on the polymer surface, ensuring flexibility while minimizing the risk of fracture.

When a voltage is applied to an IPMC for bending actuation, two main mechanisms have been proposed. First, under an electric field, the cations and anions within the polymer become polarized (Figure 28a). During this process, differences in the hydrated radii or the intrinsic size differences between the cations and anions create a volumetric imbalance, which ultimately leads to bending.^398,399^ In the second mechanism, when voltage is applied to a polyanion gel, cations migrate toward the cathode, generating a chemical gradient and thus an osmotic pressure on the cathode side (Figure 28b). The polymer chains near the cathode then swell by taking in more solvent, whereas those near the anode lose water and shrink, leading the entire material to bend in one direction.^400–402^ In either case, the ions and solvent within the polymer are the true drivers of bending, while electrons merely flow near the metal electrodes to connect to the external power source and do not traverse the polymer matrix. This distinguishes IPMC’s actuation principle from the electron-based conduction mechanism in metals or semiconductors. Although it may resemble electro-osmosis, the mechanism of IPMC works fundamentally differently. It relies on changes of ion distribution within the membrane, creating a chemical gradient that induces osmosis rather than directly producing fluid flow through ion migration.

**Figure 28:**
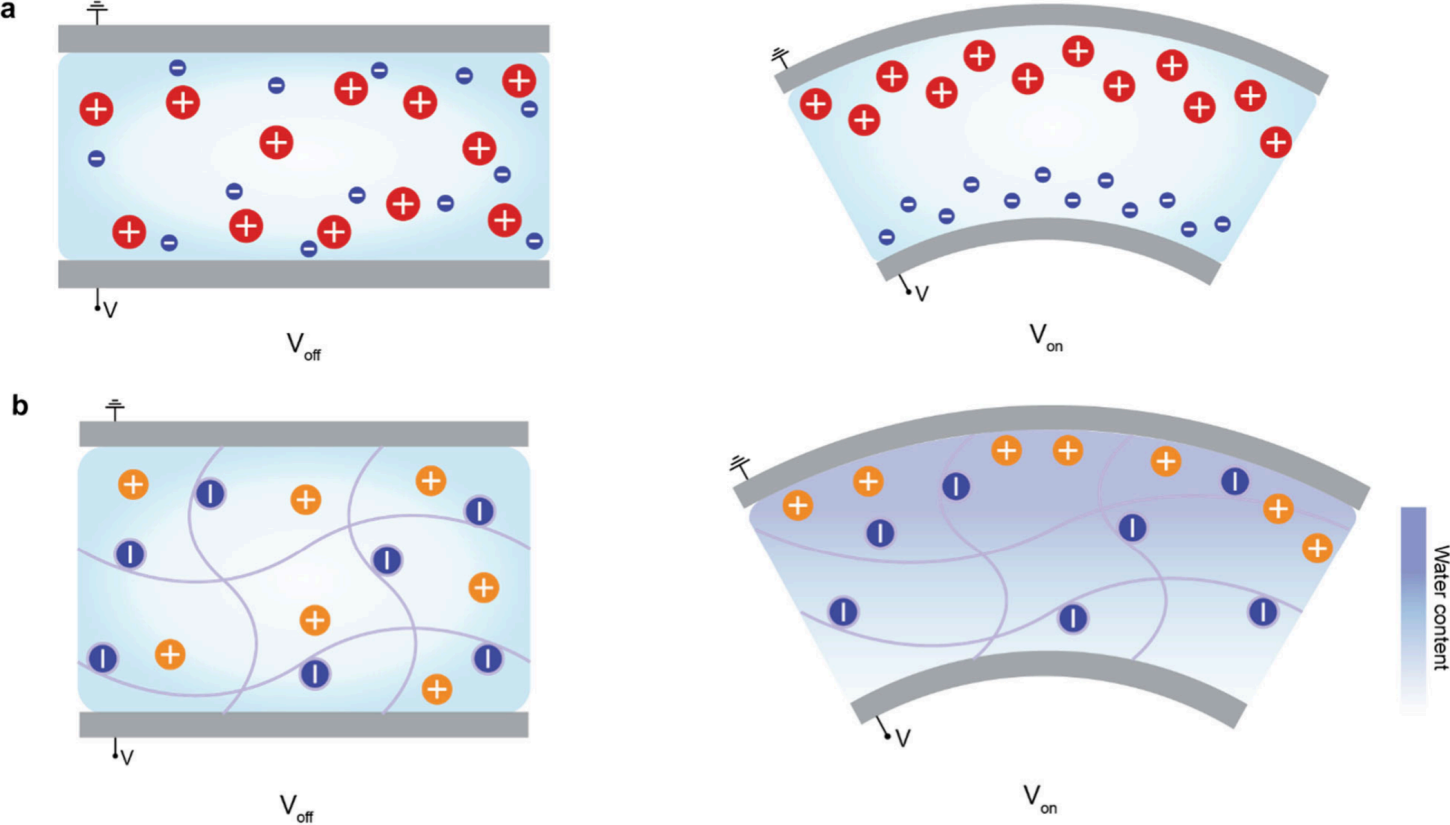
Mechanisms of ion polymer metal composite (IPMC). Two main mechanisms of IMPCs have been proposed: (a) cations and anions polarize toward the anode, causing bending due to size or hydration-radius differences, or (b) cations migrate toward the cathode, inducing an osmotic pressure that swells one side and shrinks the other. In both cases, ions and water in the gel drive the actuation, while electrons remain confined to the metal electrodes.

Because the motion of the solvent is directly induced by ion migration, effective actuation can be achieved under only a few V of applied electric potential. Thanks to its ability, IPMC remains a key candidate for low-voltage actuation systems such as soft actuators^403,404^ and artificial muscles.^405,406^ Additionally, it functions well in wet environments, making it advantageous for underwater robotics^407–409^ and direct contact with biological tissues.^410–412^

#### 5.2.2 Gel-Based IPMCs with Unique Characteristics of Ions

Early IPMCs, which relied on both conventional solvents and ions, often suffered from stability issues such as water evaporation.^395,400^ To address these concerns, Wu et al. integrated an IL into IPMC, thus mitigating solvent-related instability (Figure 29a).^399^ In addition, they employed vertically aligned nickel oxide nanowalls to maximize the electrode’s surface area, creating abundant sites for ion flooding and accumulation. The vertically aligned nanostructure facilitated rapid ion intercalation and deintercalation, leading to faster actuation. By using the enhanced stability conferred by the IL, their IPMC could operate for over 500,000 cycles.

**Figure 29:**
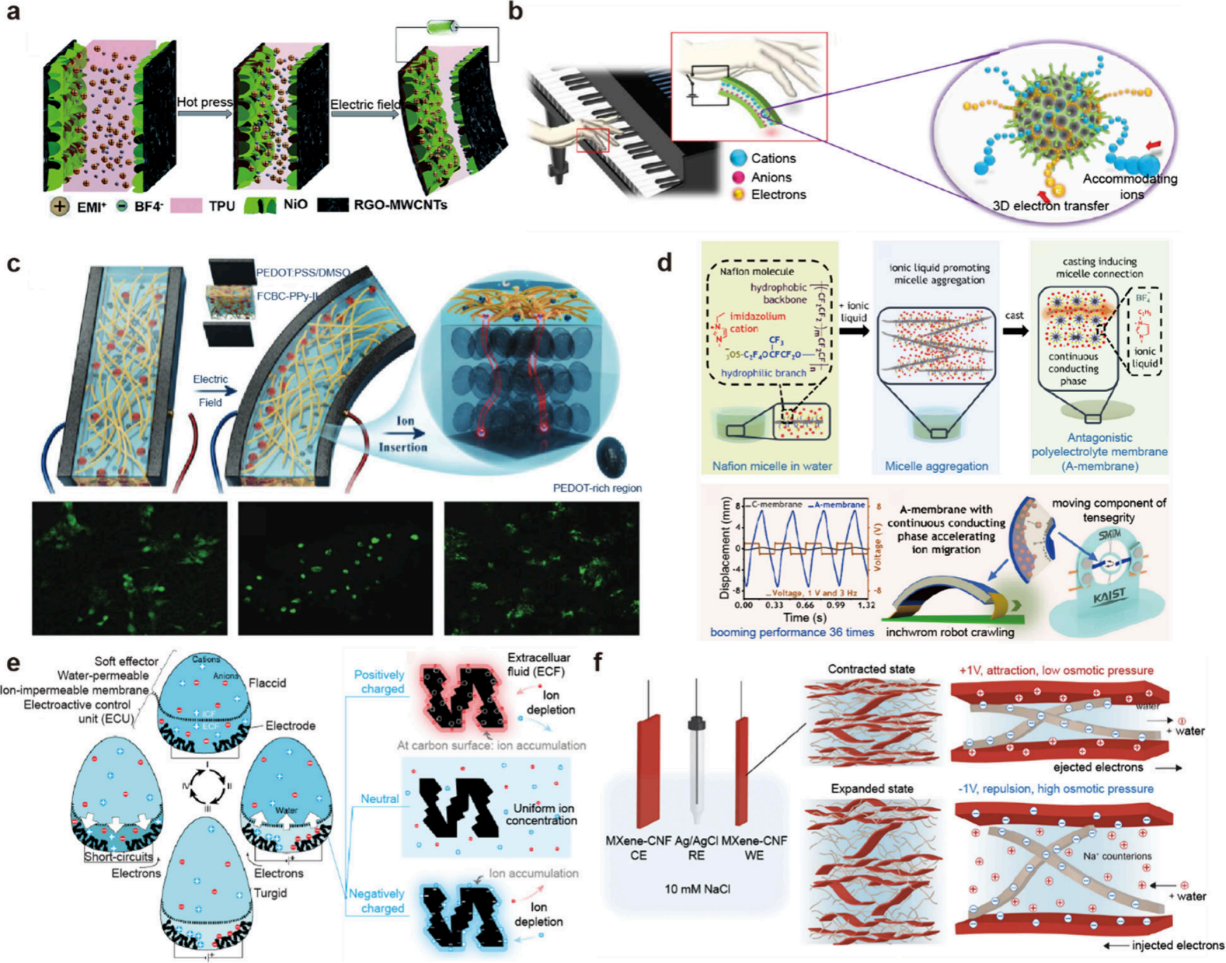
Applications of ion–polymer metal composite with gels. (a) Mitigation of water evaporation in early IPMCs through IL integration and vertically aligned nickel oxide nanowalls, enabling over 500,000 cycles and rapid ion intercalation. Reproduced with permission from ref 399. Copyright 2013 Royal Society of Chemistry. (b) High-deformation electro-ionic soft actuator with [EMIM]­[TFSI] and a covalent triazine framework in PIM-1, achieving 17.0 mm displacement at ±0.5 V and reduced phase delay. Reproduced with permission from ref 415. Copyright 2020 Springer Nature under CC BY 4.0 http://creativecommons.org/licenses/by/4.0/. (c) Biocompatible IPMC bioartificial muscles using functional carboxylated bacterial cellulose, polypyrrole nanoparticles, [EMIM]^+^[BF_4_]^−^, PEDOT:PSS, and DMSO for potential implant applications. Reproduced with permission from ref 416. Copyright 2020 John Wiley and Sons. (d) Micelle-based ion-conducting channel formation via amphiphilic Nafion and IL, resulting in a sub-1 s rise time, a 36-fold increase in bending displacement, and long-term stability. Reproduced with permission from ref 402. Copyright 2024 Springer Nature under CC BY 4.0 http://creativecommons.org/licenses/by/4.0/. (e) By mimicking plant osmotic strategies, reversible electro-osmosis and electrosorption can be harnessed to develop devices with tunable stiffness. Reproduced with permission from ref 417. Copyright 2019 Springer Nature under CC BY 4.0 http://creativecommons.org/licenses/by/4.0/. (f) Layered nanocomposite electrode gel using 2D MXene platelets and 1D cellulose nanofibrils, achieving electrical conductivity over 200 S cm^–1^, ionic conductivity above 0.1 S cm^–1^, and tensile strength near 100 MPa. Reproduced from ref 401. Copyright 2023 John Wiley and Sons under CC BY-NC-ND 4.0. http://creativecommons.org/licenses/by-nc-nd/4.0/.

Moreover, in artificial muscles and soft robotics where flexibility and low-voltage operation are paramount, IPMC shows potential for wearable devices and biocompatible robotic actuators.^413,414^ Mahato et al. developed an electro-ionic soft actuator based on IPMC that demonstrates high bending deformation under ultralow input voltages, making it suitable as a soft robotic touch finger on fragile displays (Figure 29b).^415^ They used the IL [EMIM]^+^[TFSI]^−^ for improved stability and employed a metal-free covalent triazine framework in the intrinsically microporous polymer. This design produced a surface area highly accessible to electrolytes, thereby boosting IPMC performance. The soft touch finger achieved a peak-to-peak displacement of 17.0 mm under a ±0.5 V square wave at 0.1 Hz. Its phase delay in harmonic response was one-fourth that of a pure PEDOT-PSS-based actuator.

Since IPMC actuates via ionic mechanisms, it can achieve relatively large displacements and exhibit flexible motion at voltages of only a few volts. Building on these characteristics, there have been attempts to demonstrate its potential as a biological device by achieving a stable motion using biocompatible gels. Wang et al. fabricated bioartificial muscles by creating an IPMC with functional carboxylated bacterial cellulose and PPy nanoparticles (Figure 29c).^416^ To maximize stability, they introduced the IL [EMIM]^+^[BF_4_]^−^, and they used PEDOT:PSS combined with dimethyl sulfoxide (DMSO) as the electrode materials, ensuring flexibility throughout the device. Cell tests confirmed the biocompatibility of this IPMC, demonstrating its potential as an implantable actuator within the human body.

Nguyen et al. sought to improve IPMC performance by establishing effective ion-conductive channels inside the material (Figure 29d).^402^ They synthesized amphiphilic Nafion molecules with an IL, which self-assembled into micelles that serve as continuous conduction pathways during casting. The hydrophilic–hydrophobic domains of Nafion and the electrostatic equilibrium with the IL allowed for a functionally antagonistic solvent strategy. As a result, they achieved an extremely short rise time of less than 1 s and a 36-fold increase in tip-to-tip bending displacement at 1 V. The device also demonstrated remarkable long-term stability over 42 days and a 110-fold increase in normalized blocking force.

Focusing on osmotic pressure control via ion redistribution under an electric field, Must et al. investigated the modulation of material stiffness (Figure 29e).^417^ By employing porous carbon electrodes to attract ions toward the electrode, they induced a localized ion gradient around a water-permeable, yet ion-impermeable membrane. The resulting osmotic imbalance drove water into the soft actuator, leading to swelling and an increase in modulus. This approach was inspired by the way plants regulate their stiffness through osmotic pressure. They designed a coiled soft actuator capable of anchoring or releasing objects in response to an electric field, thereby showcasing its potential utility in soft robotics.

Research has also investigated the control of electrode volume through electric-field-driven osmotic pressure. Li et al. introduced an electrode gel combined with MXene, adjusting the electrode’s charge to modulate ion concentration and induce swelling within the gel (Figure 29f).^401^ Their approach utilized 2D MXene platelets and 1D cellulose nanofibrils with extremely high aspect ratios to construct an ultrastrong nanocomposite. Through self-assembly in an aqueous medium, they formed an alternating layered structure of ∼100 nm thick dense MXene layers and cellulose nanofibrils-rich swellable sublayers. Consequently, the hydrogel with 20 wt % water achieved an electrical conductivity exceeding 200 S cm^–1^, an ionic conductivity surpassing 0.1 S cm^–1^, and a tensile strength nearing 100 MPa.

### 5.3 Electrochromic Devices

#### 5.3.1 Mechanism of Electrochromic

Electrochromic refers to the reversible change in optical properties of a material, specifically its ability to absorb or reflect light, induced by an applied external electrical signal.^418–424^ In most cases, applying a certain potential to an electrode triggers an electrochemical reaction that inserts or extracts ions (e.g., lithium ions and hydrogen ions) or electrons into or from the material (Figure 30). This process alters the electron levels or coordination environment of material, thereby modifying its intrinsic optical characteristics, such as transmittance, reflectance, and absorbance.

**Figure 30:**
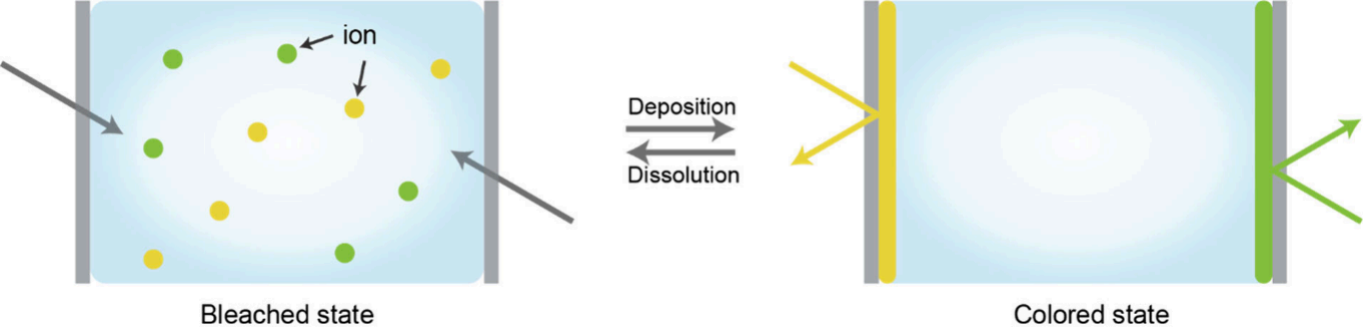
A mechanism of a gel-based electrochromic system. When an electric field is applied, the electrochromic material undergoes a redox reaction, causing it to either deposit or dissolve. By alternating between colored and bleached states, the system generates visible color changes.

A well-known example of electrochromic behavior can be seen in materials like tungsten oxide (WO_3_) or iridium oxide (IrO_2_), which undergo “coloration” and “bleaching” cycles controlled by an external voltage.^425–430^ For instance, in tungsten oxide-based materials, applying a negative potential inserts cations (mainly Li^+^ or H^+^) and electrons simultaneously (reducing W^6+^ to W^5+^), causing the color to shift from a lighter blue to a deeper blue. Conversely, a positive potential drives these ions and electrons out of the material, returning it to its original color during the “bleaching” process. The core principle here is that one can regulate or maintain the material’s color at any given time by controlling the external electrical input. Since this reversible control of oxidation states heavily depends on ion insertion and removal, ions are essential to electrochromic technology.

Such reversible changes in the ionic states enable the dynamic modulation of color in electrochromic systems. Furthermore, the stability of the ionically altered states allows electrochromic displays to retain a selected optical state for an extended duration even after the external power is removed, thereby significantly reducing energy consumption during operation.^431,432^ Due to these advantages, electrochromic materials and techniques have drawn attention across various fields, including energy-saving smart windows,^433–436^ display technologies,^437–440^ and wearable electronic devices.^441–443^ In particular, incorporating electrochromic materials into smart windows allows dynamic control over sunlight transmittance, thereby reducing the energy consumption for heating, cooling, or lighting. Efforts are also focused on scaling up production, enhancing durability, and commercializing electrochromic systems.^432,444^

#### 5.3.2 Gel-Based Electrochromic Systems with Unique Characteristics of Ions

In electrochromic devices, the ion transport layer is an essential component that facilitates the smooth migration of the ions and electrochemical reactions. This layer must contain ions and can be categorized into three types: liquid electrolytes, solid electrolytes, and gel polymer electrolytes.^445–449^ While liquid electrolytes offer high ionic conductivity, they face challenges in long-term operation due to evaporation and leakage issues. Solid electrolytes, on the other hand, exhibit excellent mechanical stability but are limited by high interfacial charge transfer resistance and low ionic conductivity. However, gel-type ion transport layers offer both mechanical stability that arises from their flexibility and stretchability with high ionic conductivity that results from their high solvent and ion content.

Chen et al. reported a high-performance electrochromic device by using an ionically cross-linked gel polymer electrolyte (Figure 31a).^450^ This device consists of fluorine-doped tin oxide (FTO) glass, a Li_*y*_NiO/gel electrolyte/WO_3_ stack, and another FTO glass. During the coloring process, Li ions move to the WO_3_ side to form Li_*z*_WO_3_, resulting in a visible color change. In this structure, the gel electrolyte, which is composed of various polymer chains such as pAA and pAAm, along with a solvent exhibits an ionic conductivity of about 1.33 × 10^–2^ S/cm. This performance surpasses that of a liquid electrolyte, which has an ionic conductivity of 2.47 × 10^–2^ S/cm. As a result, the fabricated device achieved a high optical modulation of 61% at 660 nm and fast response times of 7.5 s for coloring and 8.5 s for bleaching. Additionally, thanks to the inherent mechanical stability of the gel, the device showed excellent durability under periodic operation and long-term use. These findings highlight the advantages of using a gel-based ion transport layer in electrochromic devices.

**Figure 31:**
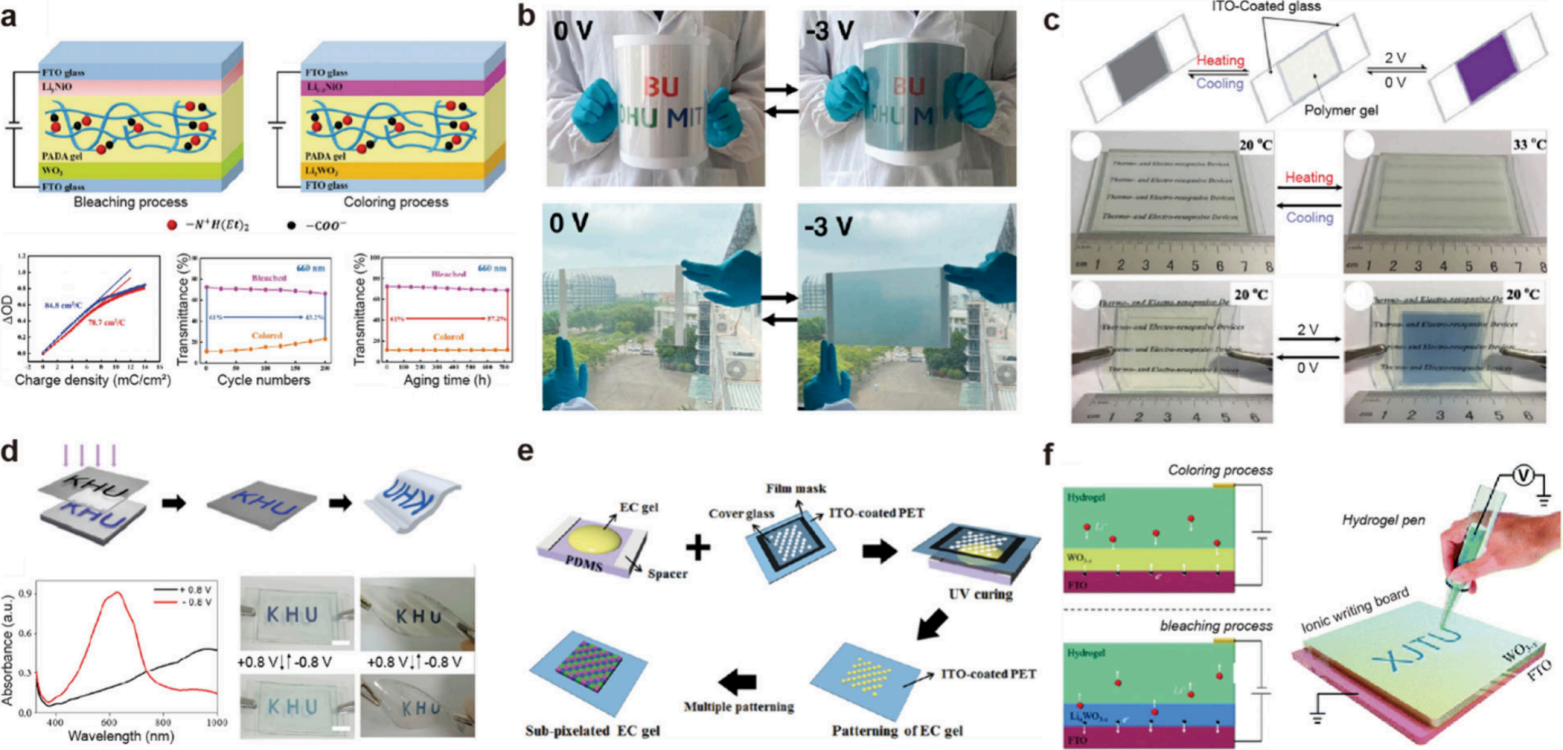
Gel-based electrochromic systems. (a) By using an ion-transport layer composed of an ionically cross-linked gel, a wide range of optical modulation and long-term stability can be achieved. Reproduced with permission from ref 450. Copyright 2013 Royal Society of Chemistry. (b) Thanks to the flexibility of the gel for ion transport, combined with MXene electrodes, the electrochromic device can be made highly flexible. Reproduced with permission from ref 451. Copyright 2021 Springer Nature under CC BY 4.0 http://creativecommons.org/licenses/by/4.0/. (c) Incorporating a thermoresponsive polymer (NIPAAm) enables both thermal and electrical stimuli control of the colored state. Reproduced with permission from ref 452. Copyright 2017 Royal Society of Chemistry. Since gels can be fabricated through various methods, gel-based electrochromic systems can be manufactured via multiple processes, including (d) transfer, (e) photopatterning, and (f) direct-ink writing. Reproduced with permission from ref 459. Copyright 2016 American Chemical Society. Reproduced with permission from ref 460. Copyright 2019 John Wiley and Sons. Reproduced with permission from ref 461. Copyright 2018 Royal Society of Chemistry.

A gel-based ion transport layer not only enhances the performance of electrochromic devices through high ion mobility but also allows for the application of stretchability and flexibility to create devices that can achieve mechanical deformation. Li et al. developed a flexible electrochromic device with fast response and high coloration efficiency by using a self-assembled 2D titanium dioxide (TiO_2_)/Ti_3_C_2_T_*x*_ heterostructure (Figure 31b).^451^ The MXene (Ti_3_C_2_T_*x*_)-based transparent electrode provides both excellent electrical conductivity and flexibility, while the 2D TiO_2_ layer, with its nanostructure, maximizes ion mobility as the electrochromic layer. A poly­(methyl methacrylate) (PMMA)-based gel containing LiClO_4_ and propylene carbonate was used as an electrolyte, enhancing the device’s flexibility. Even after 1,000 bending and releasing cycles, the electrode’s resistance increased by only about 6%, thereby demonstrating the gel electrolyte’s stability and excellent mechanical durability.

Gels, which can be fabricated by using various monomers, offer diverse functionalities through material design, thereby expanding the potential applications of electrochromic devices. Chen et al. proposed a thermo- and electro-responsive smart window by using poly­(*N*-isopropylacrylamide) (pNIPAAM), which is a classic thermoresponsive material, poly­(IL), and water (Figure 31c).^452^ A pNIPAAm typically dissolves well in water due to its hydrophilic amide groups. However, when the temperature exceeds its lower critical solution temperature (LCST), interactions between the hydrophobic isopropyl groups and the polymer backbone lead to phase separation. By exploiting this phase separation within the ion transport layer of the electrochromic device, the smart window’s transparency can be controlled by temperature. Additionally, by employing diallylviologen as the electrochromic layer, the device can change color upon an applied voltage. In short, this dual-responsive smart window system highlights the potential for developing multifunctional devices through gel-based material design.

Gels are polymer networks that contain a solvent and require cross-linking to achieve the desired mechanical properties.^453–458^ There are numerous fabrication methods for gels, and the flexibility of these methods can be directly applied to electrochromic device manufacturing. Kim et al. presented a technique to produce patterned conductive polymers via photolithography (Figure 31d).^459^ They first patterned PEDOT through a sequential solution process and then transferred the patterned PEDOT onto a hydrogel in a second gelation step. The resulting electrochromic device maintained its pattern, while demonstrating stable electrochromic switching. By leveraging gel systems to produce patterned conductive polymers that serve as electrolytes, this research shows expanded process capabilities in electrochromic device fabrication.

Further building upon the processability of gels, Kim et al. developed a multicolored, flexible electrochromic display (Figure 31e).^460^ They fabricated a device by multipatterning electrochromic pixels with a photomask using ionic-liquid-based electrochromic gels containing monoheptyl-viologen, diheptyl viologen, and diphenyl-viologen to achieve magenta, blue, and green colors, respectively. In addition, a PMMA-based ionogel served as both the ion transport layer and mechanical support, exhibiting mechanical robustness and durability through over 1,000 bending tests. This study successfully patterned the gel into subpixels about 200 μm in size, demonstrating the feasibility of a high-resolution electrochromic display.

A hydrogel is a stable solid reservoir that can contain ions and holds a solvent to facilitate ion movement. Fang et al. used hydrogel to develop an extremely simplified electrochromic device and proposed its potential use as an ionic writing board (Figure 31f).^461^ Typically, an electrochromic device comprises at least five layers, including electrodes, an electrolyte, and an ion storage layer. However, they reported a single hydrogel that functions as a transparent electrode, electrolyte, and ion storage layer, drastically simplifying the structure. By molding a pAAm-based hydrogel and a LiCl-containing hydrogel, they created a “hydrogel pen”. When this pen contacts the device’s surface, ions are injected at the point of contact, inducing localized electrochromic switching upon application of a voltage. This allows the user to “write” information on the device, which then showed an optical memory effect that can be retained without an additional power supply. Applying a reverse voltage reverts the color back to its original state, making the system rewritable. This hydrogel-based rewritable electrochromic device demonstrates broad potential for use as an information display or in smart window applications.

### 5.4 Iontophoresis

#### 5.4.1 Mechanism of Iontophoresis

Iontophoresis is a therapeutic technique to deliver ions across the skin membrane under a constant DC.^462,463^ The main advantages of iontophoresis include localized, noninvasive, convenient, and rapid administration of water-soluble, ionized medication into the skin.^464^ Typically, the applied electric current is small (0.5 mA/cm^2^ or less), and it effectively transports charged therapeutic agents such as proteins, peptides, and oligonucleotides, which otherwise require invasive parenteral administration.^465,466^ Since ion penetration correlates directly with the applied current, iontophoresis enables programmable drug delivery, reducing reliance on biological variability.^467^ Gel formulations provide an electrically conductive medium that is easy to apply, maintains optimal skin hydration, low interfacial tension, and supports convective flow, thereby enhancing transdermal drug delivery.^468^ Furthermore, drug releasing rates can be precisely controlled by adjusting the formulation characteristics of gels.^469^

The operation principles of iontophoresis are based on two electrically driven ion movements: electromigration, and electro-osmosis (Figure 32a).^470–472^ Electromigration operates on the fundamental principle of Coulomb force, that like charges repel each other. When electric current flows through the circuit, cations are repelled from the anode, whereas anions are repelled from the cathode. Hence, the ions are electrically released from gels and the electrode, allowing their controlled penetration through the skin to target sites. In electro-osmosis, a solvent flow occurs due to the electric potential difference across the charged, porous skin membrane. The electric field mobilizes free counterions within the skin toward the electrode of opposite charge, dragging water molecules along and generating a convective solvent flow. This solvent flow facilitates the simultaneous transport of neutral and charged molecules through the skin in the same direction. At physiological skin pH (approximately 4–6), the skin has a slightly negative charge, causing electro-osmotic flow to occur typically from the anode to the cathode.^473^

**Figure 32:**
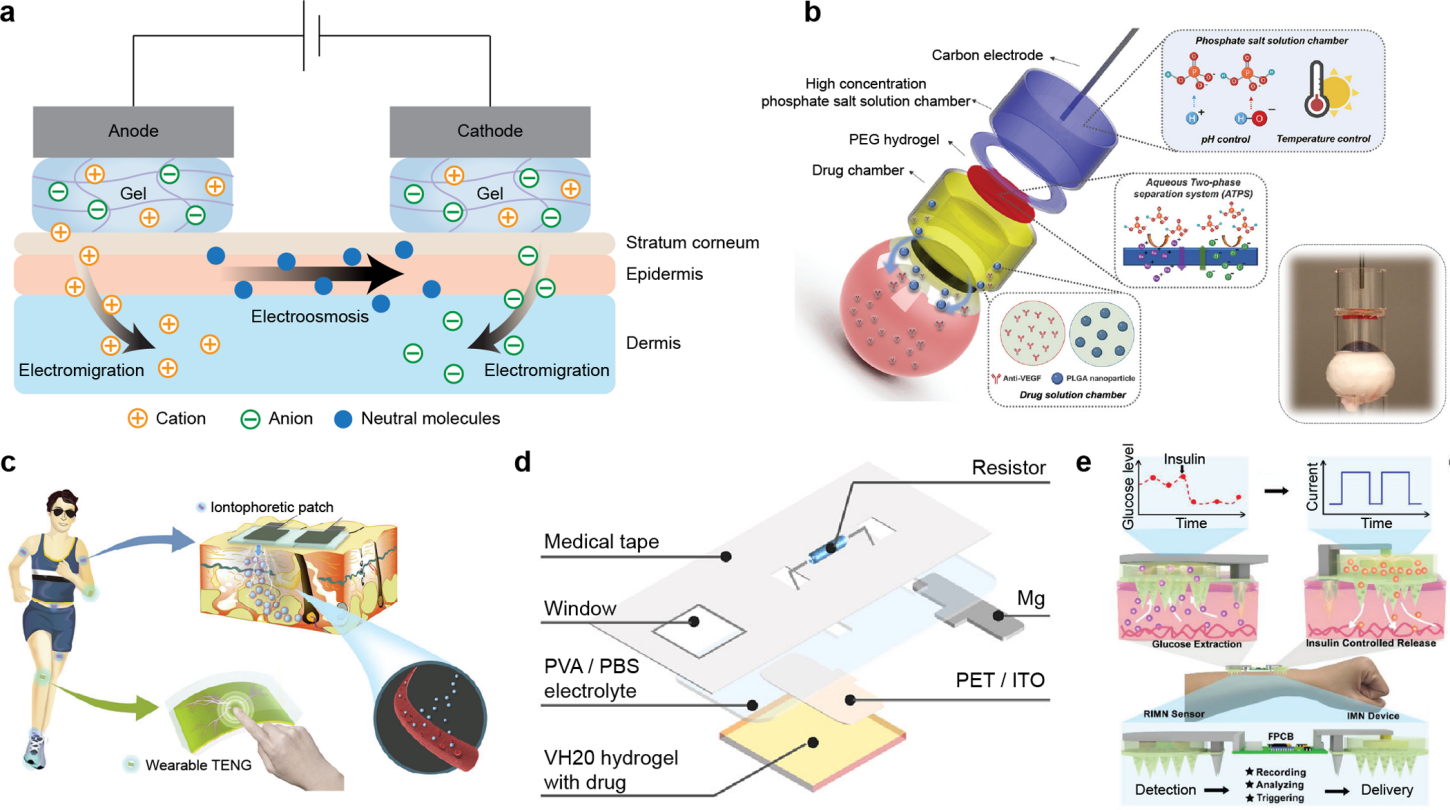
Gel-based iontophoresis. (a) Operating principles of iontophoresis with gel-based substances. (b) High-intensity iontophoresis device for intraocular delivery of macromolecules and nanoparticles, employing the PEG hydrogel for an aqueous two-phase separation. Reproduced with permission from ref 476. Copyright 2021 John Wiley and Sons. Wearable iontophoretic device powered by (c) a TENG, and (d) an integrated Mg battery, eliminating the need for external power sources. Reproduced with permission from ref 477. Copyright 2019 John Wiley and Sons, and from ref 478. Copyright 2023 Springer Nature under CC BY 4.0 http://creativecommons.org/licenses/by/4.0/. (e) A closed-loop diabetes treatment system based on mesoporous microneedle platform made of PEG. Reproduced with permission from ref 479. Copyright 2021 John Wiley and Sons under CC BY 4.0 http://creativecommons.org/licenses/by/4.0/.

Iontophoresis has been widely used in various biomedical fields such as local anesthesia, neuropsychology, muscle skeletal disorders, ophthalmology, and dermatology.^464^ Additionally, recent advancements including integration with flexible and wearable electronics has broadened its application.^474,475^ In the following section, we highlight gel-based iontophoretic applications and discuss their potential for future biomedical therapies.

#### 5.4.2 Gel-Based Iontophoresis Applications with Unique Characteristics of Ions

One of the major challenges in iontophoresis is its dependence on target ions. Despite the promising therapeutic potential of macromolecular- and nanoparticle-based ophthalmic drugs, their large molecular size significantly limits permeation through ocular tissues. Moreover, delivering these larger-sized agents through iontophoresis often requires higher current intensities, which pose risks such as water electrolysis and local Joule heating. To address these issues, Zhao et al. developed a hydrogel ionic circuit-based ocular iontophoresis device for high-efficiency (up to 87 mA cm^–2^) intraocular delivery of macromolecules and nanoparticles (Figure 32b).^476^ They introduced a PEG hydrogel matrix to form an aqueous two-phase separation of saturated phosphate salt solutions. This configuration provided three critical advantages by buffering electrochemical-reaction-induced pH changes, effectively absorbing electrode overpotential-induced heat, and significantly reducing Joule heating through its high ionic conductivity (up to 51.31 mS cm^–1^). Consequently, this approach safely enables intraocular delivery of macromolecules and nanoparticles at therapeutically relevant concentrations within 10–20 min, avoiding significant tissue damage or cellular toxicity.

Recent efforts have extended the scope of ionophoretic applications toward wearable and flexible biomedical platforms. Wu et al. reported a TENG-driven self-powered wearable device that offers on-demand therapeutic delivery without stored-energy power sources (Figure 32c).^477^ The TENG harvests biomechanical energy from diverse body movements, enabling an on-demand therapeutic release by integrated wearable power sources.

Further, Zhou et al. proposed a simplified wearable iontophoresis patch incorporating a built-in Mg battery and employing a cytocompatible viologen-based polyelectrolyte hydrogel as both a drug reservoir and cathode material (Figure 32d).^478^ They utilized hydrogel which is copolymerized from AAm and p-styrene-bipyridine monomers, providing a stable and redox-controlled response to electro-stimuli. This polyelectrolyte hydrogel surpasses conventional conducting polymers by enabling drug release at lower driven potentials, providing antibacterial activity to prevent infections, and maintaining an optimal bioelectrical interface for efficient transdermal delivery. This approach eliminates the need for external power management modules and bulky wires, enhancing practicality and patient compliance.

To expand beyond conventional iontophoresis, the structural design of noninvasive, transdermal drug delivery systems has diversified. In 2021, Li et al. presented a fully integrated closed-loop system for diabetes treatment (Figure 32e).^479^ They introduced a mesoporous microneedle platform capable of extracting glucose in situ and delivering insulin, which are typically too large to permeate the skin membrane. By integrating a flexible printed circuit board for signal recording and processing, feedback control, signal transmission, and wireless communication, this system offers an intelligent and compact solution for diabetes management. In 2024, Qu et al. reported hydrogel-based microneedle electrodes that enable both physiological monitoring and pharmaceutical intervention.^377^ Their system achieved closed-loop antiepileptic treatment with spatiotemporal controlled drug release triggered by a recorded neural signal. In the same year, Zhang et al. reported a self-powered microneedle system utilizing TENGs composed of ITO, PTFE, and PPy.^326^ Their system successfully controlled the release of optogenetically engineered extracellular vesicles. These approaches demonstrate the potential of iontophoresis in precise drug administration and targeted therapy.

Gel-based iontophoresis systems exploit ionic conduction to enable the on-demand transdermal delivery of charged therapeutic agents. Unlike rigid pumps or invasive injections, these systems allow for localized, low-voltage, and minimally invasive transport of ions across biological barriers.^480^ The soft and hydrated nature of gels provides high mechanical conformity and biocompatibility, facilitating intimate contact with dynamic or irregular tissue surfaces.^481^ Moreover, polyelectrolyte gels can be engineered to modulate ion selectivity and enable the directional migration of ions. These characteristics make gel-based iontophoresis suitable for wearable drug delivery patches, neuromodulation interfaces, and electroresponsive therapeutic systems.

## 6 Challenges and Perspectives

Despite the growing interest in gel-based ionic circuits, several challenges need to be addressed to enhance their functionalities and enable practical applications. These challenges primarily stem from the fundamental differences between electrons and ions as well as intrinsic material disparities between semiconductors or metals and gels. To unlock the full potential of gel-based ionic circuits, it is important to understand and respond to these fundamental differences. In this section, we outline several key challenges that arise from such differences and highlight emerging ideas that offer promising paths forward.

The first major distinction between electronic and ionic circuits is the lack of an ionic counterpart to the inductor. In electronic systems, inductors are passive circuit elements that store energy in magnetic fields in response to changes in the electric current. They are commonly used in energy storage, transformers, radio frequency systems, and oscillators. However, in ionic circuits, the heavy mass and low mobility of the ions prevent them from exhibiting conventional inductive behavior. To emulate inductor-like functions, researchers have instead examined the small-signal AC impedance features that emerge from coupling of rapid electronic motion and delayed ionic transport, a so-called chemical inductor.^482^ Another conceptual approach to framing the idea of an ionic inductor employs the hydraulic analogy where electrical current corresponds to fluid flow and voltage to pressure. This suggests that inductive behavior could, in principle, be mimicked by bulk ionic flow through microchannels, where the inertia of the ionic solution resists rapid acceleration or deceleration.^483^ Therefore, further studies on hybrid systems that integrate microfluidic architectures or dynamic gel–fluid composites are needed to realize time-dependent ionic behaviors analogous to inductance, which may enable signal processing or filtering functionalities in gel-based ionic circuits.

Although gel-based ionic circuits offer unique advantages, they still lag behind electronic circuits in several electrical performances due to the intrinsic difference between ions and electrons. First, typically, ionic transport times range from milliseconds to seconds, whereas electronic components operate in the nanosecond to picosecond range. This limits the feasibility of high-speed computing applications for ionic circuits. However, slower response time can be advantageous for memory and neuromorphic functions, where ionic hysteresis enables short-term information retention and synaptic plasticity.^484^ In parallel, strategies such as reducing the gel thickness, incorporating nanoscale channels, or engineering charge carriers with higher mobility may help improve ionic response times for faster signaling.

Second, ion migration generally requires higher operating voltages that exceed the electrochemical window in gel-based ionic circuits, unlike CMOS circuits that function below 1 V.^485^ To address this, researchers have explored strategies to minimize net Faradaic reactions by employing electrochemically stable electrodes and electrolytes, or by driving the system with AC.^108,315^ Furthermore, recent developments in ion-selective gels or fluidic memristors present promising avenues to reduce energy consumption and expand the voltage compatibility of ionic devices.^8,486^

Third, while electronic circuits have achieved transistor densities beyond 10^9^ devices per cm^2^ through planar lithography, ionic circuits remain constrained to the millimeter to micrometer scale. It is largely due to differences in fabrication conditions: electronic circuits are typically manufactured in dry, vacuum-compatible environments, whereas ionic circuits require wet processes involving hydrated gels or liquid electrolytes. Nevertheless, recent advances in fabrication techniques offer novel routes to improve the patterning resolution in ionic circuits. For instance, extrusion-based printing methods allow the direct deposition of ionic gels with microscale precision, enabling flexible circuit layouts without relying on conventional lithography.^487,488^ This approach also facilitates the integration of soft materials with various mechanical properties, which is particularly advantageous for conformal or wearable applications. Likewise, recent photopatterning techniques provide enhanced spatial and chemical control.^489,490^ These approaches are also compatible with multilayer fabrication schemes or 3D printing technologies, supporting the construction of more complex circuit architectures.^79,491,492^

In addition to patterning resolution, the manufacturability remains an open question. Most ionic circuits to date are laboratory-scale prototypes that rely on manual assembly or specialized processing techniques. As ionic circuits are still at the proof-of-concept stage, there are currently no widely adopted examples that extend beyond lab-scale demonstrations. To advance practical implementation, future research focusing on improving the fabrication reliability and long-term stability is needed.

Despite these various challenges, ionic circuits hold a strong potential as a bridge between biological systems and artificial systems. This potential stems from their inherent compatibility with ion-based signaling in biology, enabling direct integration with neural, muscular, and epidermal systems. For instance, ionic systems are already used in bioelectrical applications such as ECG, electroencephalography, and electromyography, where both conformable contact and low skin–electrode impedance are essential.^493^ In this context, gel-based ionic systems have attracted growing interest as biocompatible interfaces owing to their mechanical compliance and ionic conduction.^494,495^

Beyond circuit-level challenges, gel-based ionic systems also face intrinsic material limitations. These stem not only from the dynamic nature of ionic transport but also from the mechanical and electrical weaknesses of gels. For instance, the ionic conductivity of gels (1–100 mS/cm) is significantly lower than the electronic conductivity of metallic conductors (∼10^7^ S/cm).^117,496^ This results in substantial resistive losses and limits the circuit efficiency. To address this, ongoing research focuses on engineering polyelectrolyte networks,^497,498^ incorporating ILs or eutectic solvents,^329,499,500^ and introducing percolating conductive nanomaterials^501^ to enhance ion mobility and reduce internal resistance.

Moreover, gels typically exhibit a lower stiffness than semiconductors or metals, making them susceptible to swelling, deformation, and mechanical fatigue over time. While their inherent softness, stretchability, and mechanical compliance make them suitable for biomedical applications, these issues can hinder long-term reliability.^34^ Recent advances in gel design, such as the incorporation of double network systems,^453,454,502,503^ dynamic or reversible cross-linking,^504–507^ highly entangled polymer networks,^455,508–510^ and hierarchical or tensegrity structures,^511–516^ have increasingly addressed such issues by enhancing the network elasticity and structural robustness. These approaches improve not only the mechanical properties of gels but also the processability, compatibility, and stability, which are key factors for interfacing with microfabricated systems and realizing scalable ionic circuit platforms.

Lastly, unlike solid-state components that operate reliably across diverse environments, gel-based ionic circuits often require strict environmental control to maintain consistent performance. For example, under open-air conditions, solvent evaporation or leakage can significantly impede the practical deployment and long-term reliability of the ionic circuits. In particular, hydrogels are highly susceptible to external stimuli such as temperature, pH, humidity, and changes in ionic concentration.^517,518^ To address these issues, recent efforts have focused on mitigating dehydration and shielding ionic conductors by incorporating elastomeric coatings,^519–523^ or lipid bilayers,^524–526^ and adding extra salts^527,528^ to hydrogel. In parallel, ionic conductors such as organogels^529–532^ and ionogels^159,533–536^ have emerged as promising alternatives, offering comparable ionic conductivity, mechanical compliance, and longevity.

## 7 Conclusion

Gel-based ionic circuits have emerged as a promising platform for soft, flexible, biocompatible, and dynamically tunable circuit elements. Their biggest advantages are based on unique transport properties of ions, such as greater diversity, mass, and local accumulation compared to electrons, combined with the mechanically compliant nature of gels. These studies encompass traditional electronic elements such as resistors, capacitors, memristors, diodes, transistors, and power sources while also enabling synaptic plasticity behaviors.

In this review, we explored the fundamental principles, material strategies, and recent advances in gel-based ionic circuits by categorizing them into passive and active circuit elements, power sources, and noncircuit applications. The noncircuit elements highlighted in this review refer to the systems that do not conform to traditional circuit frameworks. Instead, they introduce entirely new functionalities with distinct working mechanisms, expanding the scope of ionic circuits beyond conventional paradigms.

Despite these advancements, several challenges remain that hinder the widespread adoption of gel-based ionic circuits. The need for an ionic inductor counterpart, slower ionic transport rates, and higher power consumption are key issues compared with traditional semiconductor-based electronics. Furthermore, the mechanical and electrical limitations of gels, such as low conductivity, environmental sensitivity, and swelling, present additional hurdles that must be addressed to ensure long-term stability and scalability.

Looking ahead, gel-based ionic circuits hold immense potential in human–machine interfaces, particularly in biomedical, neuromorphic, and flexible applications. As research in iontronics and bioelectronics continues to evolve, gel-based ionic circuits are poised to play a crucial role in bridging the gap between biological and artificial systems. In this pursuit of innovations, we hope that gel-based ionic circuits will bring us closer to developing more efficient, adaptive, and multifunctional electric systems in the near future.
